# The unreliability of egocentric bias across self–other and memory–belief distinctions in the Sandbox Task

**DOI:** 10.1098/rsos.181355

**Published:** 2018-11-07

**Authors:** Steven Samuel, Edward W. Legg, Robert Lurz, Nicola S. Clayton

**Affiliations:** 1 Department of Psychology, University of Cambridge, Cambridge, UK; 2 Brooklyn College, City University New York, New York, NY, USA

**Keywords:** theory of mind, egocentric bias, Sandbox Task, false belief

## Abstract

Humans are often considered egocentric creatures, particularly (and ironically) when we are supposed to take another person's perspective over our own (i.e. when we use our theory of mind). We investigated the underlying causes of this phenomenon. We gave young adult participants a false belief task (Sandbox Task) in which objects were first hidden at one location by a protagonist and then moved to a second location within the same space but in the protagonist's absence. Participants were asked to indicate either where the protagonist remembered the item to be (reasoning about another's memory), believed it to be (reasoning about another's false belief), or where the protagonist would look for it (action prediction of another based on false belief). The distance away from Location A (the original one) towards Location B (the new location) was our measure of egocentric bias. We found no evidence that egocentric bias varied according to reasoning type, and no evidence that participants actually *were* more biased when reasoning about another person than when simply recalling the first location from memory. We conclude that the Sandbox Task paradigm may not be sensitive enough to draw out consistent effects related to mental state reasoning in young adults.

## 1. Introduction

Humans are egocentric creatures. We imagine that others share our beliefs and preferences [1], our knowledge [2] and even our thirst [3]. Egocentric bias has been studied in great detail in the context of false belief reasoning, particularly in children. It is well-established that children younger than four have difficulty understanding that someone can have a false belief about something that the child herself knows to be true [4], at least when asked explicitly. However, older children have no problems understanding this distinction. Traditionally, research has explored how and why this ‘theory of mind’ (ToM) develops at this age, with relatively little focus on the same ability within adult populations, in part due to the simplicity of the task that children were asked to perform. The ‘classic’ false belief task describes how one person, protagonist A, leaves an item in one location, and how subsequently another person, protagonist B, moves the item to a different location while protagonist A is absent. The critical question involves asking the child where protagonist A believes the item to be, namely in location A or B. Clearly, such a task is too simple for adults, who perform at ceiling [5]. Instead, more sensitive measures of false belief reasoning are required for this age group.

One recent candidate is the Sandbox Task [6–12]. Based on a paradigm originally developed by Huttenlocher *et al.* [13], the Sandbox Task retains the conceptual content of classic false belief tasks but allows for a continuous rather than dichotomous response. Specifically, the item in question is hidden in a trough, or sandbox, which can also represent a variety of different spaces depending on the story (e.g. a bathtub, a garden). The item is later moved to a second location also within the sandbox, but in the absence of protagonist A. Therefore, when participants are asked to indicate where protagonist A believes the item to be, they need to indicate a location in continuous space. Egocentric bias is measured as the distance from the original location (Location A) towards the current location (Location B) of the item that the participant indicates. Adult participants have been found to show egocentric bias on this task, and more crucially still, they show greater egocentric bias when asked to reason about another person's belief about where an object is than when they are asked to simply indicate Location A based on their own memory [6,9–11]. In other words, participants are more biased by their own knowledge when they are asked to indicate where a protagonist thinks the item is (i.e. engage their theory of mind) than when they are simply asked to recall the original location without reasoning about others' mental states.

However, there are reasons to believe that egocentric bias that has previously been attributed to mentalising processes may in fact be the outcome of a more generalized difficulty with reasoning from representations, be they mental or non-mental. Recently, we tested this hypothesis using the Sandbox Task specifically. We found that participants showed greater bias when reasoning about false beliefs than when reasoning from their own memory ([6], experiment 1), but we also found that participants showed equivalent bias on false belief trials and false *film* trials, in which participants were instead instructed to indicate where a film of the moment the item was first hidden shows the object to be ([6], experiment 2). We interpreted these results as supporting the hypothesis that there is greater egocentric intrusion when reasoning from representations *generally* (i.e. not only representations of mental states such as beliefs) compared to when reasoning from one's own knowledge.

Our findings threw up a number of questions concerning the source of egocentric bias. Were participants more biased on false belief trials than memory trials because attributing false beliefs requires us to reason about others' mental states, as opposed to our own? Was it because there is something special about belief states specifically that elicits more bias than memories? Was it because in experiment 1 we asked participants where a protagonist would *look for* an item, introducing action prediction, whereas in experiment 2 we asked them where a protagonist *thought* the item was, which omitted any need to process someone's next move? We discuss briefly each of these possibilities before describing how we sought to shed more light on the source (or sources) of egocentric bias.

One possible account of how reasoning about others might explain bias on false belief trials is that in order to mentalise about another person we must first start from what we know and then, by a process of stages, reach an inference about another [14,15]. This ‘anchoring and adjustment’ process might therefore be the means by which egocentric intrusions occur, since we begin from our own point of view but have to perform an operation to distance ourselves from this that may leave some residual egocentricity in our end state. By asking participants to reason from another person's false belief, we might therefore be tapping into bias occurring through reasoning about other people, and this could explain why memory trials, which only require an assessment of one's own knowledge, elicit less bias. As a result, a more direct test of a potential role of reasoning about others would be to ask a question that does not differ in any dimension except in terms of whether it is about the ‘self’ (the participant) or the ‘other’ (the protagonist). One way to do this is to compare egocentric bias when reasoning from our own memories to bias when reasoning about the memories of other people.

Another possibility concerns whether participants show more bias when reasoning from false beliefs than when reasoning from their own memory because beliefs elicit more bias than memories. It may be, for example, that we consider beliefs to be more subjective and memories more factual, and the effect of this difference is that our own knowledge is more likely to be inhibited when we are thinking about facts. One way to test this is to ask participants to make judgements about both the beliefs and memories of other people, and then compare the degree of egocentric bias shown between these conditions. For example, it could be that reasoning from a protagonist's belief (e.g. ‘where do they *believe x* to be?’) could incur a greater bias than reasoning from the same protagonist's memory (e.g. ‘where does he/she *remember x* to be?’), because beliefs are ‘special’.

A third possibility concerns whether the type of inference that participants are asked to make influences the degree of egocentric bias they display. In our previous study ([6], experiment 1) we asked participants to indicate where a protagonist who had a false belief would ‘look for’ an item. This question type is consistent with the majority of research with adults [16], including the Sandbox Task [7,9,11]. However, ‘think’ questions (i.e. ‘where does the protagonist ‘think’ the item is?’) have been argued to be better suited to tapping mental representations than action predictions [17]. Therefore, one might predict greater bias on ‘think’-type questions than action-type questions. Alternatively, predicting an action predicated on a false belief may elicit the most bias because it requires *two* questions to be answered (where does the protagonist think the item is *and* where will the protagonist look for it?), which may introduce more noise into the decision-making process and thus allow egocentric bias greater scope for intrusion.

As a result of a consideration of these possibilities, we sought to test three hypotheses in a new Sandbox Task experiment:

(1) Some or all egocentric bias on false belief trials is due to reasoning about *others*
(2) Some or all egocentric bias on false belief trials is due to reasoning about *beliefs*
(3) Some or all egocentric bias on false belief trials is due to *predicting actions according to false beliefs*

In order to test these hypotheses, we recruited participants to three different conditions. All participants performed ‘Own Memory’ trials as controls, in which they were asked to indicate the location of an object according to where they (the participant) ‘remembered it to be’. The three groups differed, however, according to the experimental trials that they were asked to perform.

In the ‘Other Memory’ condition, participants were asked to indicate the location of an object according to where the *protagonist* remembered the object to be. The contrast between this trial type and the own memory trial type allows us to test hypothesis 1.

In the ‘Other Belief’ condition, we asked participants to reason from a protagonist's false *belief* (i.e. where does the protagonist *believe* the object to be?). By comparing the results of this condition to the results of the Other Memory condition, we can test whether there is something specific to reasoning about another person's belief rather than another person's memory that might increase egocentric bias relative to Own Memory control trials, and hence test hypothesis 2.

In the ‘Other Action’ condition, we asked participants to indicate where a protagonist who had a false belief would *look for* an item. This action prediction format is used in other studies using the Sandbox Task [6–12]. Since this trial type has rather consistently predicted greater bias than memory trials we predicted that participants would again show significant bias relative to Own Memory controls. However, we were more interested in learning whether this trial type would elicit more bias than reasoning from a protagonist's false belief in the absence of explicit mention of an action (Other Belief condition), providing a test of hypothesis 3.

Together, the results of these three conditions would help to further our knowledge of the underlying sources of egocentric bias.

## 2. Experiment 1

### 2.1. Method

#### 2.1.1. Participants

Participants were recruited online using Prolific Academic (www.prolific.ac) and were required to be between 18 and 40 years of age, English native-speakers and residents of the UK. All provided informed consent and were compensated financially for their time. A power analysis assuming a more conservative two-tailed test was conducted using G*Power and was based on the results of our previous study that found a significant difference in egocentric bias between memory trials and false belief trials using an almost identical procedure ([6], experiment 1). The analysis revealed a required sample size of 50 participants to detect medium effect sizes (*r* correlation between variables = 0.39, alpha = 0.05, power = 0.95^1^). We further calculated that this would require us to recruit just over 200 participants across the three conditions (own memory versus other memory; own memory versus other belief, own memory versus other action) in order to achieve sample sizes of 50 in each group after accounting for the predicted omission of the data of approximately 50 participants due to issues we encountered in our earlier study, such as failing the attention check trial (see below). A total of 212 people took part, of which 19 were eliminated for screen calibration issues,^2^ 4 for taking longer than the allocated 22 min to complete the task, and 1 more for not completing the task. The data from an additional 35 participants were removed for failing to indicate a location at least halfway towards the correct location (Location B) in the attention check trial. Finally, 9 data points were removed for overrunning word searches (more than 24 s) immediately prior to the test screen. Final participant numbers were 153 in total, made up of 49 in the Other Memory condition (by block order: *N* = *26 Other Memory/Own Memory; N* = *23 Own Memory/Other Memory),* 50 in the Other Belief condition (*N* = *26 Other Belief/Own Memory*; *N* = *24 Own Memory/Other Belief)*, and 54 in the Other Action condition (*N* = *23 Own Memory/Other Action; N* = *31 Other Action/Own Memory)*.

#### 2.1.2. Materials

All images^3^ were first created in Powerpoint and then converted into PNG files for presentation on Qualtrics (Qualtrics, Provo, UT, 2015). All images were in landscape (756 × 567 pixels) format, and the container (‘trough’) was 584 pixels in width and 36 pixels in depth, centrally positioned on the horizontal axis, with text (red, bold font) above. Crosses (‘x’, 15 × 15 pixels) on the troughs marked the locations where the object was hidden first (Location A) and hidden the second time (Location B). The distances the object moved between Location A and Location B were identical to those in our earlier study (as well as [11]), in terms of proportions of the total length of the trough. Specifically, the object moved 22.65% the length of the trough on short distance trials, and 45.3% on long distance trials. By including two different distances, we ensured that participants could not adopt a strategy whereby they could simply ‘work back’ from the second location they saw. The direction (left or right) and distance (short or long) of the move was fully counterbalanced (table 1). The specific co-ordinates of Location A were copied again from our earlier study [6], which in turn had been derived from Sommerville *et al*. [9], experiment 2, by converting them into proportions for use with our stimuli. After participants had finished reading the first two slides of each scenario, which set the scene for the critical question on the final slide, they were given a word search puzzle to complete before being asked the test question. The word search task was used, as in Samuel *et al*. [6], Coburn *et al*. [11] and Sommerville *et al*. [9], to prevent participants from using a perceptual strategy to answer the test questions regarding the location of the hidden object. We created slightly easier word searches for the present experiment than those we used previously, since we were keen to keep participants as engaged as possible. The word searches in the present experiment were 7 × 7 letters in size (reduced from 10 × 10), and were created by inserting six randomly-generated 3–6 letter words (created in www.textfixer.com) into a word search puzzle using www.puzzlemaker.com.

**Table 1.** Trial order, type (Own Memory or experimental), direction (Location B to left or right of Location A), distance (short or long), and coordinates of locations.

	trial order		trial type		Location A	Location B
trial	order 1	order 2	direction	distance	(% from left)	(% from left)
1	OwnMem/Exp	Exp/OwnMem	right	short	50.2	72.8
2	OwnMem/Exp	Exp/OwnMem	left	long	80.2	34.9
3	OwnMem/Exp	Exp/OwnMem	left	short	40.1	17.5
4	OwnMem/Exp	Exp/OwnMem	right	long	30.1	75.4
5	Attention check	Attention check	right	long	33.4	78.7
6	Exp/OwnMem	OwnMem/Exp	left	long	70.2	24.9
7	Exp/OwnMem	OwnMem/Exp	right	short	60.1	82.8
8	Exp/OwnMem	OwnMem/Exp	right	long	43.4	88.8
9	Exp/OwnMem	OwnMem/Exp	left	short	73.5	50.9

#### 2.1.3. Procedure

**Figure 1.**
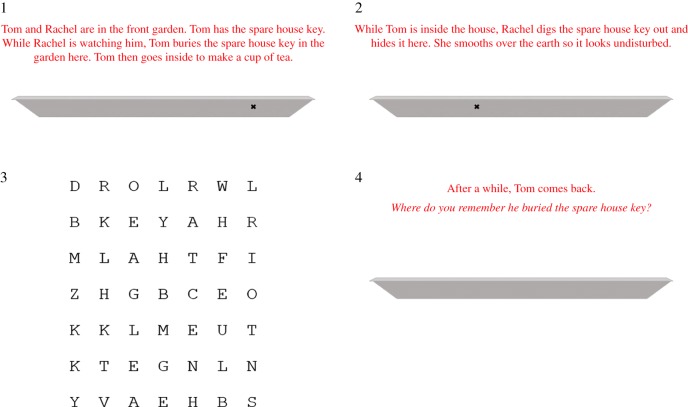
An example long-distance, Own Memory trial. On short-distance trials the area between Locations A and B was always half the area shown here.

Each condition consisted of nine trials; four Own Memory control trials, four experimental trials which varied depending on the condition, and one trial designed to check participants' attention (table 1). Each trial was formed of four slides (figure 1), which with the exception of the third (word search) slide participants could negotiate at their own pace, although they were not allowed to return to slides previously left. An example scenario began as follows: ‘Tom and Rachel are in the front garden. Tom has the spare house key. While Rachel is watching him, Tom buries the spare house key in the garden here. Tom then goes inside to make a cup of tea.’ An ‘x’ on the trough, depicted below the text, illustrates the first location in which the object was hidden (Location A). The second slide continued as follows: ‘While Tom is inside the house, Rachel digs the spare house key out and hides it here. She smooths over the earth so it looks undisturbed.’ An ‘x’ on the trough below the text displays the new location (Location B) of the object. On the third slide, participants were presented with the word search, which remained on screen for a fixed period of 20 s. Participants were instructed to type into a box below the puzzle as many words as they could spot in the allotted time. The fourth and final slide of each scenario (which came after the word search slide) contained a brief preamble: ‘After a while, Tom comes back.’ On the same slide, depending on the condition, participants were asked one of the following critical questions: on Own Memory trials (present in all conditions), ‘Where do you remember he buried the spare house key?’; on Other Memory trials, ‘Where does Tom remember he buried the spare house key?’; on Other Belief trials, ‘Where does Tom believe the spare house key to be?’, and on Other Action trials, ‘Where will Tom look for the spare house key?’. In each case, participants indicated their response by clicking on an empty trough under the text. Crucially, with the exception of this critical question, the scenarios were identical in each condition. Based on a notional 20 s per slide average and 10 min to read the online consent form, instructions and debrief, we excluded from the analyses any data from participants who spent longer than 22 min on the task. A full list of scenarios is provided in the electronic supplementary material.

In each of the four control and four experimental trials the text described how one protagonist saw an object hidden in location A (slide 1) but did not see it being moved and hidden in location B (slide 2). Crucially, the correct response in every Own Memory trial and every experimental trial was always Location A, so any differences between conditions could only be attributable to the wording of the instructions themselves. We recorded where on the screen (in pixels) the participant clicked, and used the difference between the location indicated by the participant and Location A as a measure of bias. Where participants clicked on a location closer to where they knew the object to be, the resulting score would be positive (indicating bias towards the participants' knowledge of the object's true location). If instead it was further, the score would be negative. In the attention check trial the vignette instead described a protagonist who saw an object being hidden in both Location A *and* Location B, and the participant was simply asked where the object is. As in our earlier study, to ensure that participants were paying attention to the task we excluded from the analyses all participants who failed to indicate a location closer to Location B than Location A on this trial. Since this was the only trial type in which Location B was the correct response, any strategy that involved the consistent selection of Location A would therefore lead to failure on this trial and exclusion from the results.

Following previous research [6,9], we organized each condition into two blocks of four divided by the single attention check trial. Participants were randomly assigned to a version with the Own Memory block coming either before or after the experimental block.

#### 2.1.4. Analyses

Bias scores were converted from pixels into percentages of the total length of the trough, with positive scores indicating that the participant gave a location x% closer to the Location B relative to Location A, and negative scores a location in the opposite direction (i.e. further from Location B than Location A). For example, a location between Location A and Location B in the example given in figure 1 would indicate positive bias. A location to the right of Location A in this example would indicate negative bias. As in our previous study [6] the response data were not normally distributed, so we opted for non-parametric tests. Any exceptions are clearly stated in the text alongside the analyses in question.

### 2.2. Results

We began by testing whether the order in which the conditions were performed (Own Memory trials first or experimental trials first) influenced performance. Independent-samples Mann–Whitney *U* tests revealed no significant order-related effects for any experimental trial type (Other Memory *p* = 0.733; Other Belief *p* = 0.741; Other Action *p* = 0.882), or for Own Memory trials in the Other Belief (*p* = 0.437) or Other Memory (*p* = 0.437) conditions. However, there was a slight trend towards greater bias on Own Memory trials when they came after Other Action trials compared to when they came before them (*Mdn* = 0.6% and *Mdn* = 0.1% respectively, *Z* = 1.732, *p* = 0.083). Given that this result did not reach significance, we analysed these data collapsed over experiment order, but also report separate order-based analyses for those participants in the Other Action condition.^4^

As an initial test, we investigated whether participants were biased towards Location B each time they had to indicate Location A, via whatever instruction. Collapsed over distance, and in the case of Own Memory trials also collapsed over the three conditions (since every participant did own memory trials), participants were biased towards Location B on Own Memory trials (*Mdn* = 2.4%, *W*(153) = 9203.5, *Z* = 6.035, *p* < 0.001, *r* = 0.34), on Other Memory trials (*Mdn* = 2.3%, *W*(49) = 870, *Z* = 2.561, *p* = 0.010, *r* = 0.26), on Other Belief trials (*Mdn* = 2.4%, *W*(50) = 983, *Z* = 3.335, *p* = 0.001, *r* = 0.33) and on Other Action trials (*Mdn* = 2.0%, *W*(54) = 1089, *Z* = 2.983, *p* = 0.003, *r* = 0.29), in each case indicated by one-sample Wilcoxon signed-rank tests. In sum, participants were biased towards the true location of the object regardless of whether the trial instructed them to respond according to their own memory, another's memory, another's false belief, or to predict another's action.

#### 2.2.1. Results by condition

**Figure 2.**
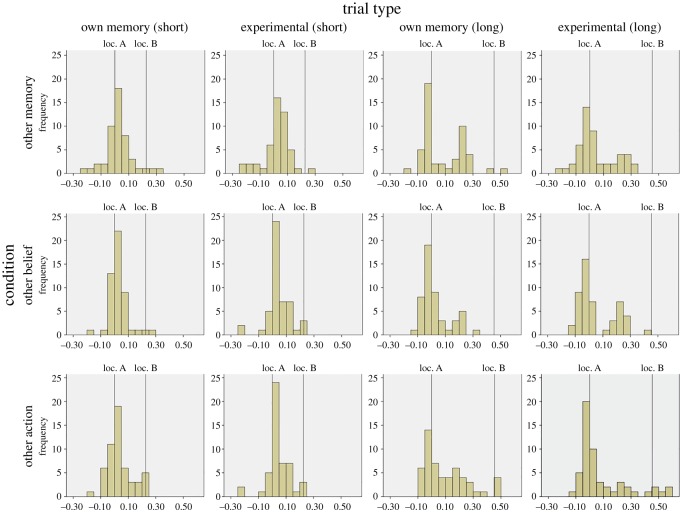
Experiment 1. Frequency distribution (in number of participants) of responses for each condition. The *x*-axis represents the % of total trough length towards (+ve) or away from (−ve) the object's true location.

As expected, the distribution of responses was not normal (figure 2), though they were also not particularly bimodal. As a result, we used non-parametric analyses on these data.

##### 2.2.1.1. Other Memory

**Figure 3.**
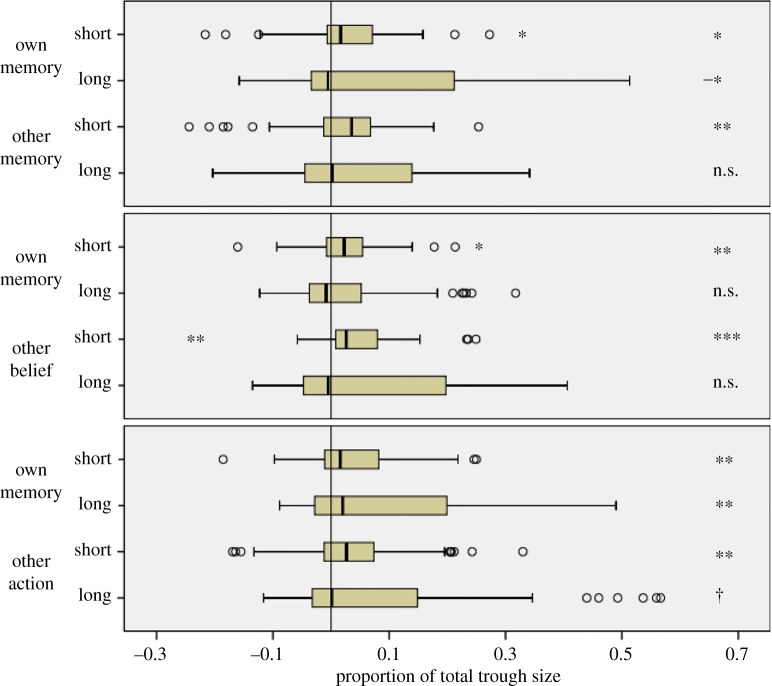
Experiment 1. Bias as a proportion of the total trough size. Outliers beyond 1.5 × IQ and 3 × IQ indicated by circles and stars respectively. From a baseline of zero, † = bias marginally significant (*p* < 0.1); * = bias significant at *p* < 0.05 level (with a minus sign, this indicates *negative* bias); ** = bias significant at *p* < 0.01 level; *** = bias significant at *p* < 0.001 level.

Figure 3 (top panel) displays the results of the Other Memory condition, split by short- and long-distance trials. In this condition, and collapsed over distance, one-sample Wilcoxon signed-rank tests found that participants were biased on both Own Memory trials (*Mdn* = 2.0%, *W*(49) = 943, *Z* = 3.288, *p* = 0.001, *r* = 0.33) and Other Memory trials (*Mdn* = 2.3%, *W*(49) = 870, *Z* = 2.561, *p* = 0.010, *r* = 0.26). However, matched-pair tests found that bias did not differ between these two trial types, *W*(49) = 465, *Z* = 1.467, *p* = 0.142, *r* = 0.15. Splitting the data into short- and long-distance trials, we found that participants were biased on short-distance Own Memory trials (*Mdn* = 1.7%, *W*(49) = 856, *Z* = 2.422, *p* = 0.015, *r* = 0.24), but showed significant *negative* bias on long-distance Own Memory trials (*Mdn* = −0.5%, *W*(49) = 858, *Z* = 2.442, *p* = 0.015, *r* = 0.25). Participants were biased on short Other Memory trials (*Mdn* = 3.5%, *W*(49) = 855, *Z* = 2.412, *p* = 0.016, *r* = 0.24), but not on long-distance Other Memory trials (*Mdn* = 0.2%, *W*(49) = 736, *Z* = 1.228, *p* = 0.219, *r* = 0.12). In line with the result of the analysis collapsed over distance, no difference between bias on Own Memory trials and Other Memory trials was found on either short-distance trials, *W*(49) = 614, *Z* = 0.015, *p* = 0.988, *r* = 0.02, or long-distance trials, *W*(49) = 503, *Z* = 1.089, *p* = 0.276, *r* = 0.11. In sum, the results failed to support hypothesis 1; egocentric bias is not the result of reasoning about others.

##### 2.2.1.2. Other Belief

Figure 3 (middle panel) displays the results of the Other Belief condition, split by short- and long-distance trials. In this condition, and collapsed over distance, participants were biased on both Own Memory trials (*Mdn* = 1.7%, *W*(50) = 946, *Z* = 2.978, *p* = 0.003, *r* = 0.30) and Other Belief trials (*Mdn* = 2.4%, *W*(50) = 983, *Z* = 3.335, *p* = 0.001, *r* = 0.33). However, bias did not differ between these two trial types, *W*(50) = 758, *Z* = 1.163, *p* = 0.245, *r* = 0.12. Splitting the data into short- and long-distance trials, we found that participants were biased on short-distance Own Memory trials (*Mdn* = 2.3%, *W*(50) = 946, *Z* = 2.978, *p* = 0.003, *r* = 0.30), but not on long-distance Own Memory trials (*Mdn* = −0.9%, *W*(50) = 674, *Z* = 0.352, *p* = 0.725, *r* = 0.04). There was also significant bias on short Other Belief trials (*Mdn* = 2.6%, *W*(50) = 1090, *Z* = 4.368, *p* < 0.001, *r* = 0.44), but not on long-distance Other Belief trials (*Mdn* = −0.5%, *W*(50) = 740, *Z* = 0.989, *p* = 0.322, *r* = 0.10). In line with the result of the analysis collapsed over distance, no difference between bias on Own Memory trials and Other Belief trials was found on either short-distance trials, *W*(50) = 790.5, *Z* = 1.477, *p* = 0.140, *r* = 0.15, or long-distance trials, *W*(50) = 731.5, *Z* = 0.907, *p* = 0.364, *r* = 0.09.

##### 2.2.1.3. Other Action

Figure 3 (bottom panel) displays the results of the Other Action condition, split by short- and long-distance trials. In this condition, and collapsed over distance, participants were biased on both Own Memory trials (*Mdn* = 4.3%, *W*(54) = 1225, *Z* = 4.154, *p* < 0.001, *r* = 0.40) and Other Action trials (*Mdn* = 2.0%, *W*(54) = 1089, *Z* = 2.983, *p* = 0.003, *r* = 0.29). However, bias did not differ between these two trial types, *W*(54) = 653, *Z* = 0.771, *p* = 0.441, *r* = 0.07. Given the marginally significant order effect we reported above, we repeated this analysis after dividing this group of participants into those who performed Own Memory trials before or after other action trials. Regardless of whether participants did Own Memory trials first, *W*(23) = 163, *Z* = 0.760, *p* = 0.447, *r* = 0.11, or Other Action trials first, *W*(31) = 168, *Z* = 1.568, *p* = 0.117, *r* = 0.23, neither showed any difference in the degree of bias between Own Memory trials and Other Action trials. Splitting the data into short- and long-distance trials (now collapsed over order), we found that participants were biased on short-distance Own Memory trials (*Mdn* = 1.5%, *W*(54) = 1080, *Z* = 2.906, *p* = 0.004, *r* = 0.28), as well as on long-distance Own Memory trials (*Mdn* = 3.7%, *W*(54) = 1135, *Z* = 3.380, *p* = 0.001, *r* = 0.33). Participants showed significant bias on short Other Action trials (*Mdn* = 2.7%, *W*(54) = 1001, *Z* = 2.527, *p* = 0.011, *r* = 0.25), but only a tendency to be biased on long-distance Other Action trials (*Mdn* = 0.02%, *W*(54) = 953, *Z* = 1.812, *p* = 0.070, *r* = 0.17). Consistent with the result of the analysis collapsed over distance, no difference between bias on Own Memory trials and Other Action trials was found on either short-distance trials, *W*(53) = 719, *Z* = 0.031, *p* = 0.975, *r* = 0.00, or long-distance trials, *W*(54) = 594, *Z* = 1.279, *p* = 0.201, *r* = 0.12.

#### 2.2.2. Comparing conditions

The preceding analyses surprisingly revealed no evidence of greater bias on experimental trials than when reasoning from one's own memory. Nevertheless, it may still be the case that the degree of bias showed varied according to condition. Recall that our second hypothesis contends that some or all egocentric bias is attributable to reasoning about beliefs, and this can be tested by measuring the degree of bias in the Other Belief condition to the degree of bias in the Other Memory condition. Additionally, our third hypothesis contends that some or all of egocentric bias is due to predicting actions predicated on false beliefs, which can be calculated by comparing bias in the Other Action condition to bias in the Other Belief condition.

We calculated difference scores that measured bias on experimental trial types (Other Memory, Other Belief, or Other Action) against a baseline of bias on Own Memory trials. Since these scores were normally distributed, we conducted a one-way ANOVA on Group (Other Memory versus Other Belief versus Other Action). This revealed an overall tendency towards a main effect of this factor, *F*_2,150_ = 2.421, *p* = 0.092, and a significant quadratic interaction, *F*_1,150_ = 4.566, *p* = 0.034. Although follow-up pairwise comparisons between all three conditions using the Bonferroni correction revealed no significant differences (Other Memory *M* = −2.3%, 95% CI [−5.5, +0.8]; Other Belief *M* = +2.0%, 95% CI [−0.7, +4.8]; Other Action *M* = −1.2%, 95% CI [−4.0, +1.7]; all *p*s > 0.1), the interaction appeared to be driven by *negative* bias (locations further *away* from Location B than even Location A) in the groups that performed Other Memory trials and Other Action trials compared to positive bias in the group that performed Other Belief trials.

Given the unexpected pattern of results, which favoured null results and even negative bias scores, we capitalized on the ability to use parametric tests on these normally-distributed difference scores in order to conduct separate one-sample Bayesian *t*-tests for each condition, asking the question of whether the data supported the null that no difference existed between bias on Own Memory trials and bias on experimental trials. We adopted the convention of a BF_10_ value of 0.33 or smaller, suggesting the data are at least three times as likely under the null hypothesis than the alternative, as a benchmark of a null result of sufficient sensitivity [18]. Recall that in all these analyses we are using the measure of bias on experimental trials with bias on Own Memory trials subtracted from this figure.

The result for the Other Memory trials was a BF_10_ value of 0.066 on a non-significant *t*-test, *t*(48) = 1.515, *p* = 0.136, indicating that the data were 15 times more likely under the null hypothesis (indeed, recall that bias was negative on this trial type). For the Other Belief condition the result was a BF_10_ value of 0.808 on a non-significant *t*-test, *t*(49) = 1.498, *p* = 0.141, suggesting that the data were 1.2 times more likely under the null. For Other Action trials, the result was a BF_10_ value of 0.087 on a non-significant *t*-test, *t*(53) = 0.825, *p* = 0.413, suggesting that the data were 11 times more likely under the null. Bias in this condition was also negative. In sum, in all three cases the null result was supported, and in the cases of Other Memory and Other Action trials this support met the criterion for useful evidence for a null.

We next applied Bayesian tests to the data in terms of hypotheses involving contrasts *between* conditions. Given that negative bias was found on both Other Memory trials and Other Action trials, and that bias did not significantly differ from zero even on Other Belief trials, these comparisons are provided merely for completeness, but may also be misleading in that they compare positive bias values to negative bias values. Concerning our second hypothesis, the analyses found that the data were three times more probable under the alternative that bias was greater when reasoning from another's false beliefs than from another's memories (BF_10_ = 3.021). Concerning our third hypothesis, the data were 11 times more likely under the null than under the alternative hypothesis that bias was greater on Other Action trials than Other Belief trials (BF_10_ = 0.085).

We performed one final test of hypothesis 1, based on our prediction that bias should be greater on experimental trials than Own Memory trials if reasoning about others is enough to create a difference. We collapsed over all three conditions in order to achieve the highest statistical power possible before testing this with a Bayesian *t*-test. Average bias was in fact *negative* (*M* = −0.5%), and the Bayesian analysis revealed a BF_10_ value of 0.060 on a non-significant *t*-test, *t*(152) = 0.595, *p* = 0.553, suggesting that the data were 17 times more probable under the null hypothesis.^5^

### 2.3. Discussion

Participants showed evidence of bias towards the true location of the object in all three experimental conditions. However, we found no evidence that participants were more biased on these trials than Own Memory control trials. In sum, we found no evidence that participants were more biased towards where they knew an object to be when they were reasoning about other people rather than about their own memories (hypothesis 1). We also found no evidence that bias was different when reasoning about others' beliefs and others' memories (hypothesis 2), or when reasoning about others' actions and others' beliefs (hypothesis 3). Additionally, whereas in our previous study [6] we found a median of only 0.6% bias on memory control trials, which was not significant (*p* = 0.143, *r* = 0.11), Own Memory trials in each of the three conditions in the present study always reached significance, and effect sizes ranged from *r* = 0.26–0.40.

We explored whether a subtle difference in the wording of memory control trials in the present study might provide an explanation for the unexpected presence of significant bias on these trials. Specifically, in the present version, the Own Memory trial asked: ‘Where do you remember x buried object y?’. In our previous study, the question was instead: ‘Where did x bury y before x left?’. One possibility is that the invocation of memory in the verb ‘remember’, as well as the presence of the word ‘you’, led to participants engaging a more subjective cognitive process, perhaps allowing for a greater degree of bias than those participants in our previous study for whom memory questions were perhaps more ‘factual’. This may in turn have compressed the difference between these memory control trials and the experimental trials.

In order to test this possibility, we recruited new participants to compare performance on both of these trial types. Specifically, if the Own Memory questions engage a different process—one that might elicit greater bias than the memory trials in our previous study—then participants should show greater bias on the Own Memory questions using ‘Where do you remember x buried y?’ than what we now term Fact Recall trials using ‘Where did x bury y?’. They may also fail to show any bias on Fact Recall trials at all, much as in our previous research [6]. We removed the time clause ‘before x left’ in order to better match the two sentence types.

## 3. Experiment 2

### 3.1. Methods

We recruited 72 new participants to a new task in which four questions were Own Memory trials which were identical to those used in the previous experiments in this paper, and four questions were Fact Recall trials in which participants were simply asked: ‘Where did x bury y?’. As before, the order in which question types were presented was counterbalanced. The data from 11 participants were removed for failed calibration, 5 for failing the attention check trial, and a further 2 did not complete the task. Of the 54 remaining participants, 24 participants performed the Fact Recall trials first and 30 the Own Memory trials first. In all other ways, the procedure was identical to those described earlier.

### 3.2. Results

**Figure 4.**
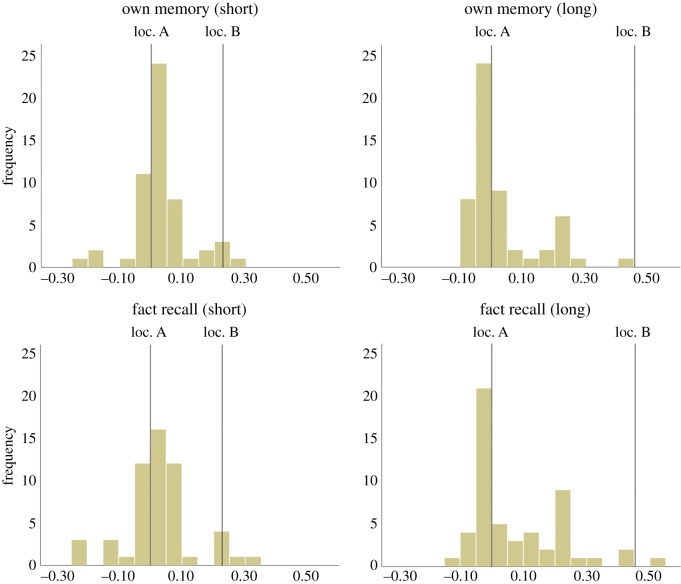
Experiment 2. Frequency distribution (in number of participants) of responses for each condition. The *x*-axis represents the % of total trough length towards (+ve) or away from (−ve) the object's true location.

Again, the distribution of responses was not normal (figure 4), and we used non-parametric analyses on these data. Mann–Whitney *U* tests revealed no evidence of any effect order (Own Memory: first *Mdn* = 0.7%; second *Mdn* = 0.7%, *U*(54) = 336, *Z* = 0.418, *p* = 0.676, *r* = 0.060; Fact Recall: first *Mdn* = 1.6%; second *Mdn* = 4.5%, *U*(54) = 429, *Z* = 1.201, *p* = 0.230, *r* = 0.160), so we collapsed over this factor.

**Figure 5.**
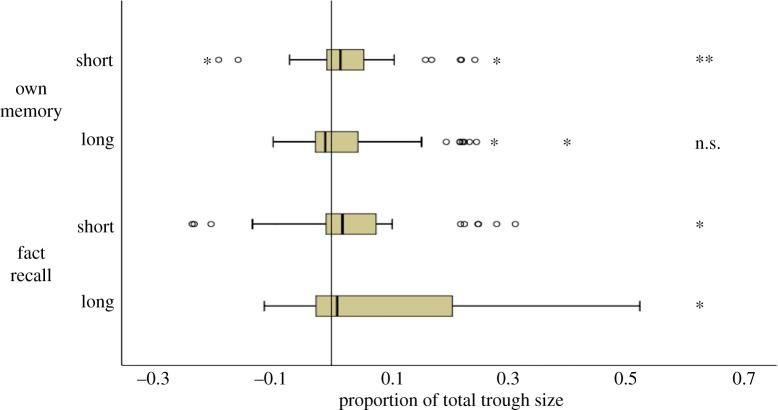
Experiment 2. Bias as a proportion of the total trough size. Outliers beyond 1.5 × IQ and 3 × IQ indicated by circles and stars respectively. From a baseline of zero, * = bias significant at *p* < 0.05 level (with a minus sign, this indicates *negative* bias); ** = bias significant at *p* < 0.01 level.

Figure 5 displays the results split by short- and long-distance trials. Collapsed over distance, one-sample Wilcoxon signed-rank tests found that participants were biased on both Own Memory trials (*Mdn* = 1.0%, *W*(54) = 1016, *Z* = 2.355, *p* = 0.019, *r* = 0.23) *and* Fact Recall trials (*Mdn* = 2.2%, *W*(54) = 1176, *Z* = 3.733, *p* < 0.001, *r* = 0.36). A matched-pair test found that bias was in fact marginally larger on Fact Recall than Own Memory trials, *W*(54) = 949, *Z* = 1.778, *p* = 0.075, *r* = 0.17. Splitting the data into short- and long-distance trials, we found that participants were biased on short-distance Own Memory trials (*Mdn* = 1.5%, *W*(54) = 1093, *Z* = 3.018, *p* = 0.003, *r* = 0.29), but not on long-distance Own Memory trials, where bias was also numerically negative (*Mdn* = −1.1%, *W*(54) = 756, *Z* = 0.116, *p* = 0.907, *r* = 0.01). Participants were biased on short Fact Recall trials (*Mdn* = 1.9%, *W*(54) = 1035, *Z* = 2.518, *p* = 0.012, *r* = 0.24), and on long-distance Fact Recall trials (*Mdn* = 1.0%, *W*(54) = 1032, *Z* = 2.493, *p* = 0.013, *r* = 0.24). No difference between bias on Own Memory trials and Fact Recall trials was found on short-distance trials, *W*(54) = 861, *Z* = 1.020, *p* = 0.308, *r* = 0.10, but on long-distance trials greater bias was found on Fact Recall trials, *W*(54) = 1008.5, *Z* = 2.290, *p* = 0.022, *r* = 0.22.

The distribution of difference scores (Fact recall – Own Memory) was normal, with a mean of 2.4% greater bias on Fact Recall trials. We ran Bayesian tests of the null hypothesis on a one-sample *t*-test, *t*(53) = 1.855, *p* = 0.069, finding the data was 1.4 times more likely under the alternative than null hypothesis. Note that this analysis thus demonstrates only very weak support, but in favour of greater bias on *Fact Recall* trials. In sum, we found no evidence that Own Memory trials elicit greater bias than Fact Recall trials, and hence the hypothesis that the reason we found significant bias in Experiment 1 in this study but not in our previous work might be because the new wording might increase bias relative to the old was not supported. In fact, there was a tendency towards the contrary; participants in this experiment showed slightly more bias on Fact Recall trials than on Own Memory trials.

## 4. General discussion

In our first experiment, we gave young adult participants one of three different versions of the Sandbox Task. Although each condition included the same control trials, in which participants were instructed to make inferences from their own memory, the three conditions varied as to whether judgements were also to be made about another's memory, another's false belief, or another's action based on a false belief. Despite adhering closely to previous versions of the task that *have* found differences in egocentric bias as a function of the type of inference participants are asked to make, we found no evidence that participants were more biased when making any of these three forms of judgements about the protagonist relative to simply extracting the equivalent information from the participant's own experience. In a second experiment, we also tested whether the wording of control trials in these experiments may somehow have elicited greater bias than the wording used in our previous study ([6], experiment 1). Contrary to the hypothesis, we found the wording used in our previous research actually elicited somewhat greater bias than the wording in the present study. As a result, the pattern of results cannot be explained by a change in the wording of own memory trials.

Although these results appear surprising in the context of our previous findings, as well as those of other studies with young adults ([9] (experiments 1 and 2), [10,11] (experiments 1 and 2)), an absence of a difference between control and false belief trials is not unheard of in the literature. The study by Bernstein *et al*. [7], though not reporting an analysis of the two trial types at the within-group level, also appears to have found no difference between memory trials and false belief trials (as suggested by fig. 1 in that study). Our results seem to be consistent with the results of that study, and extend them to other conditions, namely other action and other memory.

Not only did we find no important differences in bias between (and indeed within) conditions, but Bayesian analyses supported the null result (i.e. within conventional assessments of a useful null) for both Other Memory and Other Action trials relative to Own Memory controls, and also offered support, albeit weaker, for the null on Other Belief trials. Taken together with the overall *negative* bias scores for Other Memory and Other Action trials, any comparisons in the degrees of bias between conditions is essentially redundant. In any case, there was no instance of one condition eliciting more bias than another, even when comparing negative bias to positive bias. In fact, it is somewhat misleading to term negative bias ‘bias’ at all, since there is no theoretical framework (certainly within the theory-of-mind literature) to explain why participants should provide locations further from the true location than even the distance to the first location.

Interestingly, however, negative bias too is not unheard of on the Sandbox Task. For example, Mahy *et al*. [8] tested young children (3–7-year-olds) on a single-trial Sandbox Task and found some instances of negative bias (i.e. indicating a location further from the Location B than even Location A) in each age group (experiment 1). Since their experiment began with Location A almost at the very edge of the trough, and Location B in the middle, it is plausible that the study may have revealed *greater* negative bias had there been more room between the first location and the closest edge. This possibility is also suggested by instances, particularly in the youngest children in that study, of locations being indicated which ‘overshoot’ even the second location by up to as much as the distance between the first and second location. Such variance might be expected in young children, and on a single-trial task with no baseline measure of participant memory or response accuracy more generally. However, our negative bias results were particularly interesting in that they were calculated from a within-subjects baseline of bias on Own Memory trials, and in young adults. That is, whereas negative bias on a single trial could perhaps be explained by blunt inaccuracy or simple forgetting, negative bias measured from this baseline would suggest such ‘noise’ is taken into account.

Overall, it appears unlikely that some factor in the design of the present experiment led it to differ from those which *have* found differences between memory trials and false belief trials in the past, including the research that we have previously conducted. For example, we based our sample size on a power analysis founded on our previous study. Moreover, by adding two further conditions, the present study consisted in effect of three failed replications of that paradigm (or two if we limit these instances to those which clearly refer to false beliefs or actions predicated upon them). Secondly, we included an attention check trial, and removed the data from participants who did not indicate a location at least halfway towards the second location on this trial type, as we did in our previous work. This is important because it provides evidence that the participants in our final sample not only paid attention to subtle changes in wording between scenarios, but also that they were less likely to ignore the otherwise functionally redundant second location. Thus, we might reasonably expect *greater* bias in our experiments than in other studies where participants potentially began to filter out the second location and attend exclusively to the first.^6^ Thirdly, we chose the appropriate analysis type for the data that we obtained, using non-parametric analysis except where tests found the distribution of data did not deviate from normality. Given that our experience is that the data from this paradigm are almost always positively skewed, it may be that performing parametric analyses may inflate the size of bias. Relatedly, deviation from the mean or median also tends to be particularly large, even for groups of adults (figure 2), but especially so in children, where they can be many times higher even than the mean bias scores they correspond to [8].

Why then did we find no evidence of bias on any experimental trial type relative to own memory controls? Firstly, let us consider what we can rule out as potential explanations. Firstly, our study was modelled on previous versions of the Sandbox Task. For example, our Other Action condition used ‘look for’ questions of the type previously utilised in multiple previous iterations of the task. Crucially, if egocentric bias is stronger when reasoning from representations, or about other people, we should not expect results to occur only when using a specific set of stories, or even when using particular question wordings, as previous studies and meta-analyses have shown false belief tasks to produce consistent results despite procedural variations ([19–21], see [22] and [4] for a meta-analysis including such tasks). Our blocked design of four trials of each type with a true belief trial at the mid-point is also consistent with previous research [6,9]. Relatedly, we found no evidence that order of presentation influenced bias on experimental trials at all, and our analysis of performance on first trials alone also found no evidence of greater bias on experimental trials than control trials. Where our version of the task differed from previous ones, we feel that these differences served to improve upon existing designs. For example, previous experiments have sometimes employed fixed orders [9,11], employed only one false-belief trial [8,12] and sometimes have not used control trials [8]. Our attention check procedure improves the chances that those participants whose data were included in the analyses were paying attention to the scenarios.

So, given what is *unlikely* to explain our results, what might actually account for them? The first candidate for why participants were biased on all trial types concerns the possibility that participants are biased at least in part by features inherent to the shape of the trough. Indeed, it was such questions that motivated in part the original Sandbox study by Huttenlocher and colleagues [13]. It is a common finding that if participants, including young adults, are presented with a dot within a defined space and are then asked to reproduce the location of the dot within the same (now empty) space, they will tend to indicate a location that is biased towards the central point of a self-generated (i.e. not visible in the stimulus) categorical organization of that space, based on its symmetrical properties and edges. For example, if a participant is shown a dot very close to the upper left edge of a circle, participants are more likely to indicate a location consistent with a subdivision of the circle into four quadrants, with the dot now being located closer to the centre of the circle if it was nearer the edge (or conversely, further from the centre if it was nearer the centre, in line with a more ‘central’ location within that quadrant) [23]. Similar findings are reported with troughs, with children erring towards the midline between the ages of 2 and 6 years, but away from the midline and away from the edges towards prototypical mid-points of each ‘half’ of the rectangle after around 10 years [13]. There is also evidence that this effect of category centring is more pronounced for stimuli presented in the right visual field, further implicating categorization processes, which are lateralized to the left hemisphere [24]. It is therefore plausible that participant bias on the Sandbox Task is not entirely related to mental state reasoning or indeed reasoning from representations but is at least in part the result of self-generated categorical subdivisions that lead participants to err towards the centre of one of these categories. For a number of reasons, the potential for spatial categorization to influence responses is difficult to assess using the present design. We based our location coordinates on those used in previous research in order to better equate our design to a previous study that had found significant bias reportedly specific to mental state reasoning in adults. These locations vary seemingly at random in terms of their initial locations, and hence we did not control either for distances from category centre points in the first location or second locations, or indeed how many such category boundaries a move to a second location would cross. Indeed, the slight tendency for there to be more reliable evidence of bias on short- than long-distance trials may be related to the idiosyncrasies of the locations A and B relative to such subdivisions of the space, though this is admittedly speculative. Additionally, our counterbalancing procedure meant that the same locations would come under all different trial types (Other Belief in one, Own Memory in another, etc.), and we did not obtain measures of our participants' spatial working memory capacity, more of which is related to decreased spatial category biases [25].

A similar possibility is that participants are biased towards the second location not because it represents a mental state but because it is a competitor location within the same response space (i.e. the trough/sandbox). This would be consistent with early research into spatial biases in both adults and children. For example, Kosslyn *et al.* [26] found that participants in both age groups judged the distance between two objects to be smaller if the objects were perceived to be within the same space compared to when they were divided by an opaque barrier. In every trial type in the present experiment, participants were more biased towards the second ‘true’ location than the first, even on control trials, a result consistent with findings by Sommerville *et al*. ([9], experiment 2), who reported bias on control trials in children that indicated responses on average half way between the first and second locations, and Begeer *et al*. [12], also with children, who reported bias on memory trials larger even than the difference between bias on false belief trials and memory trials. In adults too, there are suggestions in the raw data of bias towards the true location even on control trials ([7,9] (experiment 1), [10,11]). This bias is not merely greater dispersion of responses around a point; it is directional (i.e. towards the true location).

Taken together these two findings in the literature, the subdivision of space into categories with a ‘pull’ towards their centres, and the subjective experience of distances between two objects being ‘shorter’ if they are not separated by a barrier, offer potential alternative explanations, either separately or in combination, for why participants seem to show bias regardless of instruction type. In some instances, such as single-trial versions of the task where the first location is very close to the edge of the trough and the second location quite central, spatial biases such as these might indeed be a serious confound ([8], experiment 1). However, even if this is the case, it can only be a *partial* account of bias in participants' responses, as in each case bias in young adults is usually found to be *greater* on false belief than control trials in young adults (with the study by Bernstein *et al*. [7] the possible exception), and therefore any effects related to spatial categories cannot account for the entirety of the difference between trial types in these studies.

The third candidate that we must consider is that our *previous* results may have been in some respect anomalous, and that in fact young adult participants do *not* generate more egocentric bias from representations than they do when working from their own memory. This is because we found no evidence of bias on experimental trials versus Own Memory trials in *all three* different conditions in the present experiment. As mentioned previously, it also seems possible that Bernstein *et al*. [7] also found no difference between false belief and memory control in their study with young adults. It therefore appears that the most parsimonious conclusion, given that there are now approximately four studies that show no increase in bias on trials involving mental representations (the present three conditions, and potentially the study by Bernstein and colleagues [7]) compared to six^7^ that do ([6] (experiment 1), [9] (two experiments), [11] (two experiments)), and one study showing equivalent bias on false belief trials and false film trials ([6], experiment 2), is that it is not yet clear that the Sandbox Task is a sensitive enough measure to *consistently* tease out subtle processing differences that young adults may display when mentalising as compared to reasoning in other ways. One possibility is that publication biases, whereby studies that report statistically significant findings are more likely to be published than studies which do not (e.g. [27]), have unintentionally inflated the perceived reliability of the task. Another possibility is that people *do* show greater bias on false belief trials, but the true effect size has been overestimated, perhaps in part due to the previous point.

## 5. Conclusion

Given the pattern of results across Sandbox Task studies in the literature, it would appear that there is evidence that the paradigm is sensitive enough to detect bias when reasoning from representations as compared to reasoning from one's own memory or some other non-representational control. Nevertheless, the data from young adult participants is more equivocal. As we have shown, participants in this age group show similar bias when reasoning from false films as when reasoning from false beliefs [6], and now it appears that the difference between reasoning from any form of representation and reasoning from one's own memory is not a robust finding. More research is required to understand why some groups of young adults show the expected difference on the task, and others not.

## Supplementary Material

SOM 1 - Materials

## Supplementary Material

SOM 2 - data for experiment 1

## Supplementary Material

SOM 3 - data for experiment 2

## Footnotes

1 For completeness, mean bias on memory trials was 1.4% (s.d. = 5.5), on false belief trials 5.1% (s.d. = 6.8).

2 Prior to the first experimental trial, participants were requested to click in the centre of 6 crosses. The crosses represented the end points of the full width of the trough, and as such were precisely 584 pixels apart. Any participants who showed an average of more than 4 pixels away from this length did not have their data included in the analyses. In this way, we excluded those participants whose computer display may have warped the visual stimuli, or whose response mechanism may have been too imprecise to allow for an accurate measure of ‘true’ performance.

3 All original materials for both this experiment and experiment 2 can be found in the electronic supplementary material.

4 Following the suggestion of one anonymous reviewer, we also conducted a comparison of performance on Own Memory trials (*n* = 70) and experimental trials (*n* = 81) based exclusively on performance on the *first trial of the task*, capturing performance prior to any attenuating effects of practice on the size of bias. A Mann–Whitney *U* test found no difference between bias on Own Memory trials (*Mdn* = −1%) and bias on experimental trials (*Mdn* = 0%), *U*(151) = 2445.5, *Z* = 1.454, *p* = 0.146, *r* = 0.12. Bias on Own Memory first trials differed significantly from zero, but in the opposite direction (i.e. bias was *negative*), *W*_70_ = 732, *Z* = 2.543, *p* = 0.011, *r* = 0.30. Bias on experimental first trials did not differ significantly from zero, *W*(81) = 1376.5, *Z* = 0.817, *p* = 0.414, *r* = 0.091. Overall, the analysis of first trials found no support for greater bias on experimental than Own Memory first trials, which corroborates the pattern of results taking into account all the trials participants performed.

5 We also tested whether those 35 participants who were *not* included in the analysis for failing the attention check trial would show any difference from this main group. We found no difference between bias on experimental trials and bias on Own Memory trials in this group, *t*(34) = 1.264, *p* = 0.215. Moreover, and consistent with the main analysis, bias was again numerically negative (*M* = −2.4%).

6 One reviewer suggested that our attention check trial would not filter out participants who were responding randomly and made a ‘false hit’, or who were responding strategically and opted to indicate points on the trough close to Location A except when they perceived some change in the wording of the critical question (without necessarily comprehending the question). However, random responding would have been picked up across performance on experimental trials, and would be indexed by an average location approximately halfway between Locations A and B, but this was not the case. As for strategic responding, we see no reason to believe that participants opted to base their responses on changes in the perceptual patterns of words on the screen rather than simply reading the instructions. We would also like to highlight that our analysis of first trials corroborates our overall pattern of results. Ultimately, our attention check procedure means our results are less susceptible to such criticisms than those from studies which included participants' data regardless of their performance on the attention check trial.

7 The descriptive results of the study by Bernstein and colleagues [10] also suggested that bias was greater on false belief than memory trials, and in all age groups tested, but statistical tests of the difference were not provided. We analysed the data, which was made available with the publication. We first found that the data were not normally distributed, so we performed non-parametric tests. These confirmed that participants showed more bias on False Belief trials than Memory trials in the age groups 1–3 year-olds, 9–10 year-olds, younger adults and older adults, but not in 5 year-olds and 11–12 year-olds. However, when we exclude those participants who indicated a location closer to the original than final location of the hidden object on *true belief* trials (i.e. those who would have been excluded by our procedures), we find first that the number of exclusions renders statistical analyses almost impossible in 9–10 year-olds (*N* reduced from 26 to 6) and 11–12 year-olds (*N* reduced from 15 to 4). In pre-schoolers, only 4 of 43 even gave a location within Location A and Location B on the true belief trial. In younger and older adults, the *N* was cut by half (from 111 to 55 and from 70 to 36 respectively). Young adults were still more biased on false belief than control trials (effect size 0.094).

## Data accessibility

Complete materials for all experiment are contained in the electronic supplementary material, SOM 1. We are unable to make the raw set-up of the experiments publicly available as they are hosted within the Qualtrics environment, but readers with a Qualtrics account can contact us (ss2391@cam.ac.uk) if they would like us to share this set-up with them. The data for Experiment 1 and Experiment 2 are in the electronic supplementary material, SOM 2 and SOM 3, respectively.

## References

[1] Ross L, Greene D, House P 1977 The ‘false consensus effect’: an egocentric bias in social perception and attribution processes. J. Exp. Soc. Psychol. 13, 279–301. (10.1016/0022-1031(77)90049-X)

[2] Bernstein DM, Atance C, Loftus GR, Meltzoff A 2014 We saw it all along: visual hindsight bias in children and adults. Psychol. Sci. 15, 264–267. (10.1111/j.0963-7214.2004.00663.x) PMC3640979 15043645

[3] Van Boven L, Loewenstein G 2003 Social projection of transient drive states. Pers. Soc. Psychol. Bull. 29, 1159–1168. (10.1177/0146167203254597) 15189611

[4] Wellman HM, Cross D, Watson J 2001 Meta-analysis of theory-of-mind development: the truth about false belief. Child Dev. 72, 655–684. (10.1111/1467-8624.00304) 11405571

[5] Zaitchik D, Koff E, Brownell H, Winner E, Albert M 2006 Inference of beliefs and emotions in patients with Alzheimer's disease. Neuropsychology 20, 11–20. (10.1037/0894-4105.20.1.11) 16460218

[6] Samuel S, Legg EW, Lurz R, Clayton NS In press. Egocentric bias across mental and non-mental representations in the Sandbox Task. Q. J. Exp. Psychol. (10.1177/1747021817742367) 30362406

[7] Bernstein DM, Thornton WL, Sommerville JA 2011 Theory of mind through the ages: older and middle-aged adults exhibit more errors than do younger adults on a continuous false belief task. Exp. Aging Res. 37, 481–502. (10.1080/0361073X.2011.619466) 22091578

[8] Mahy CE, Bernstein DM, Gerrard LD, Atance CM 2017 Testing the validity of a continuous false belief task in 3- to 7-year-old children. J. Exp. Child Psychol. 160, 50–66. (10.1016/j.jecp.2017.03.010) 28426950

[9] Sommerville JA, Bernstein DM, Meltzoff AN 2013 Measuring beliefs in centimeters: private knowledge biases preschoolers’ and adults’ representation of others’ beliefs. Child Dev. 84, 1846–1854. (10.1111/cdev.12110) 23581849

[10] Bernstein DM, Coolin A, Fischer AL, Thornton WL, Sommerville JA 2017 False-belief reasoning from 3 to 92 years of age. PLoS ONE 12, e0185345 (10.1371/journal.pone.0185345) 28957366 PMC5619768

[11] Coburn PI, Bernstein DM, Begeer S 2015 A new paper and pencil task reveals adult false belief reasoning bias. Psychol. Res. 79, 739–749. (10.1007/s00426-014-0606-0) 25183385

[12] Begeer S, Bernstein DM, Aßfalg A, Azdad H, Glasbergen T, Wierda M, Koot HM 2016 Equal egocentric bias in school-aged children with and without autism spectrum disorders. J. Exp. Child Psychol. 144, 15–26. (10.1016/j.jecp.2016.05.017) 26687336

[13] Huttenlocher J, Newcombe N, Sandberg EH 1994 The coding of spatial location in young children. Cogn. Psychol. 27, 115–147. (10.1006/cogp.1994.1014) 7956105

[14] Tamir DI, Mitchell JP 2010 Neural correlates of anchoring-and-adjustment during mentalizing. Proc. Natl Acad. Sci. USA 107, 10 827–10 832. (10.1073/pnas.1003242107) PMC2890763 20534459

[15] Epley N, Keysar B, Van Boven L, Gilovich T 2004 Perspective taking as egocentric anchoring and adjustment. J. Pers. Soc. Psychol. 87, 327–339. (10.1037/0022-3514.87.3.327) 15382983

[16] Rubio-Fernández P, Glucksberg S 2012 Reasoning about other people's beliefs: bilinguals have an advantage. J. Exp. Psychol. Learn. Mem. Cogn. 38, 211–217. (10.1037/a0025162) 21875251

[17] Leslie AM, Thaiss L 1992 Domain specificity in conceptual development: neuropsychological evidence from autism. Cognition 43, 225–251. (10.1016/0010-0277(92)90013-8) 1643814

[18] Dienes Z 2014 Using Bayes to get the most out of non-significant results. Front. Psychol. 5, 781 (10.3389/fpsyg.2014.00781) 25120503 PMC4114196

[19] Cohen AS, Sasaki JY, German TC 2015 Specialized mechanisms for theory of mind: are mental representations special because they are mental or because they are representations? Cognition 136, 49–63. (10.1016/j.cognition.2014.11.016) 25490129

[20] Cohen AS, German TC 2010 A reaction time advantage for calculating beliefs over public representations signals domain specificity for ‘theory of mind’. Cognition 115, 417–425. (10.1016/j.cognition.2010.03.001) 20350721

[21] Perner J, Aichhorn M, Kronbichler M, Staffen W, Ladurner G 2006 Thinking of mental and other representations: the roles of left and right temporo-parietal junction. Soc. Neurosci. 1, 245–258. (10.1080/17470910600989896) 18633791

[22] Schurz M, Radua J, Aichhorn M, Richlan F, Perner J 2014 Fractionating theory of mind: a meta-analysis of functional brain imaging studies. Neurosci. Biobehav. Rev. 42, 9–34. (10.1016/j.neubiorev.2014.01.009) 24486722

[23] Huttenlocher J, Hedges LV, Corrigan B, Crawford LE 2004 Spatial categories and the estimation of location. Cognition 93, 75–97. (10.1016/j.cognition.2003.10.006) 15147930

[24] Laeng B, Peters M, McCabe B 1998 Memory for locations within regions: spatial biases and visual hemifield differences. Mem. Cognit. 26, 97–107. (10.3758/BF03211373) 9519700

[25] Crawford LE, Landy D, Salthouse TA 2016 Spatial working memory capacity predicts bias in estimates of location. J. Exp. Psychol. Learn. Mem. Cogn. 42, 1434–1447. (10.1037/xlm0000228) 26900708 PMC4993700

[26] Kosslyn SM, Pick HL Jr, Fariello GR 1974 Cognitive maps in children and men. Child Dev. 45, 707–716. (10.2307/1127837) 4143827

[27] De Bruin A, Treccani B, Della Sala S 2015 Cognitive advantage in bilingualism: an example of publication bias? Psychol. Sci. 26, 99–107. (10.1177/0956797614557866) 25475825

