# Supplementary material for: The unreliability of egocentric bias across self–other and memory–belief distinctions in the Sandbox Task
Source: R Soc Open Sci. 2018 Nov 7;5(11):181355. doi: 10.1098/rsos.181355 (PMC6281948; doi:10.1098/rsos.181355)
Supplement: SOM 1 - Materials [file rsos181355supp1.pdf]

Samuel, Legg, Lurz, & Clayton: Castles Made of Sand (Supplementary Online Materials 1)

Please note that access to the Qualtrics experiments themselves can be obtained from the first author(ss2391@cam.ac.uk) if you have a Qualtrics account.

Word searches are included at the bottom of this document as they were not printed automatically by the Qualtrics exporter.

Contents

Exp. 1
Own Memory – Other Memory (2-40)
Other Memory – Own Memory (41-79)
Own Memory – Other Belief (80-118)
Other Belief – Own Memory (119-157)
Own Memory – Other Action (158-196)
Other Action – Own Memory (197-235)

Exp 2.
Own Memory – Fact Recall (236-274)
Fact Recall – Own Memory (275-313)

Wordsearches listed by names of protagonists in the trial that they accompany (314-322)

NEW sandbox: ownmem-othermem

Start of Block: Prolific Info

Q65 Before you start, please:

– maximize your browser window;
– switch off phone/e-mail/music & anything else distracting
- and please enter your Prolific ID [it can be found at the top of this webpage or when going to your account info]:\\\

[To continue to the next question at any point in the survey please use the arrow button at the bottom right of the page]

____

End of Block: Prolific Info

Start of Block: Participant Info

Q54 Before you decide to take part in this study it is important for you to understand why the research is being done and what it will involve. Please take time to read the following information carefully and discuss it with others if you wish. A member of the team can be contacted if there is anything that is not clear or if you would like more information. Take time to decide whether or not you wish to take part.

Page Break

Q55 This study investigates how adults reason about what others want, believe and act. The study fits in with a larger research theme of how children and adults reason about other individuals' mental states and how both children and adults attribute beliefs and desires to other people.

Page Break

Q56 During the study you will be asked to make judgments about what others can see, hear or do. You may also be asked to predict how another person would act based on what they have seen or heard. The experiment will take approximately 10 minutes to complete.

Page Break

Q57 Participation in the study is entirely voluntary and you can withdraw at any point. The results of the study may be written up or presented at conferences. Your participation will remain confidential and if individual data is presented there will be no means of identifying the individuals involved.

Page Break

Q58 If you would like to know more about the study or have any questions you can contact Steven Samuel (ss2391@cam.ac.uk) for further information.

The project has received ethical approval from the Psychology Ethics Committee of the University of Cambridge.

Page Break

End of Block: Participant Info

Start of Block: Consent Page 1/6

Q81 I confirm that I have read and understand the participant information.

- ○ Yes (1)
- ○ No (2)

Q93 I have had the opportunity to ask questions and had them answered.

- ○ Yes (1)
- ○ No (2)

Q105 I understand that all personal information will remain confidential and that all efforts will be made to ensure that I cannot be identified (except as might be required by law).

- ○ Yes (1)
- ○ No (2)

Q117 I agree that data gathered in this study may be stored anonymously and securely, and may be used for future research.

- ○ Yes (1)
- ○ No (2)

Q129 I understand that my participation is voluntary and that I am free to withdraw at any time without giving a reason.

- ○ Yes (1)
- ○ No (2)

Q141 I agree to take part in this study.

- ○ Yes (1)
- ○ No (2)

End of Block: Consent Page 1/6

Start of Block: Consent Page 2/6

Q66 There are two parts to the study.

First you will be asked to click on various locations on your screen (this helps us with calibration).

Second (the main part of the study) you will read some short stories about objects being hidden. Please pay careful attention to the story and the accompanying visual information. After each story you will have 20 seconds to find words in a wordsearch. After each wordsearch you will be asked a question about the story that you have just read. Total experiment time is approximately 10 minutes.

The calibration process is next...

End of Block: Consent Page 2/6

Start of Block: Consent Page 3/6

Start of Block: Consent Page 4/6

Start of Block: Consent Page 5/6

Start of Block: Consent Page 6/6

Start of Block: Start

Q17
Please click right in the centre of the left cross (first) and right in the centre of the right cross (second). Try to be as accurate as possible. If the mouse-controlled cross does not align perfectly with the crosses on the screen (for example it always has to be a fraction to the left or right), please do the same for each cross. Make sure a marker is left on each cross before moving on to the next page. You can try as many times as you like.

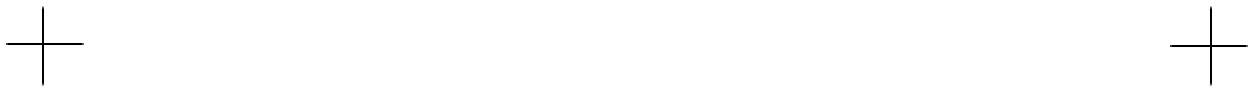

Page Break

Q48
Now please click right in the centre of this left cross and right in the centre of this right cross. Try to be as accurate as possible, and again if the mouse cannot fall exactly in the centre please ensure you do the same for each click. You can try as many times as you like.

Page Break

Q49

And finally, please do the same for the two crosses on this page.

Page Break

Q47 Browser Meta Info
Browser (1)
Version (2)
Operating System (3)
Screen Resolution (4)
Flash Version (5)
Java Support (6)
User Agent (7)

End of Block: Start

Start of Block: Block1

Q1

Sally and Jack are outside playing in the sandpit. Sally has a red toy dog. While Jack is watching her, Sally buries the red toy dog in the sand here. Sally then goes inside to get a drink of water.

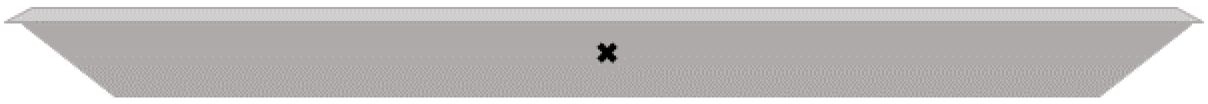

Page Break

Q2

While Sally is inside the house, Jack digs the red toy dog out and hides it here. He smooths over the sand so it looks undisturbed.

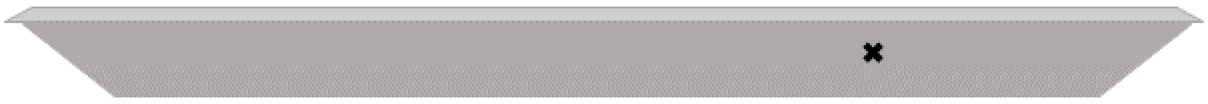

Page Break

Q3
Now try to find as many words as you can in the puzzle below and type them in the field at the bottom of the page. You have 20 seconds!

____

____

____

____

____

Q5 Timing
First Click (1)
Last Click (2)
Page Submit (3)
Click Count (4)

Page Break

Q4
Please click on the image to answer.

After a while, Sally comes back.

Where do you remember she buried the red toy dog?

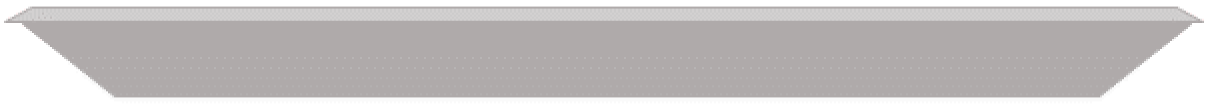

End of Block: Block1

Start of Block: Block2

Q12

Tom and Rachel are in the front garden. Tom has the spare house key. While Rachel is watching him, Tom buries the spare house key in the garden here. Tom then goes inside to make a cup of tea.

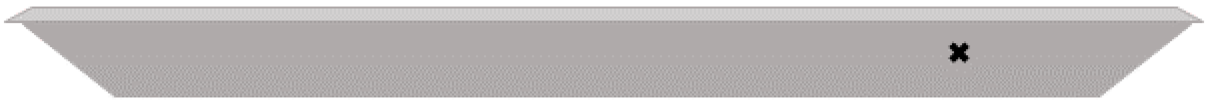

Page Break

Q13

While Tom is inside the house, Rachel digs the spare house key out and hides it here. She smooths over the earth so it looks undisturbed.

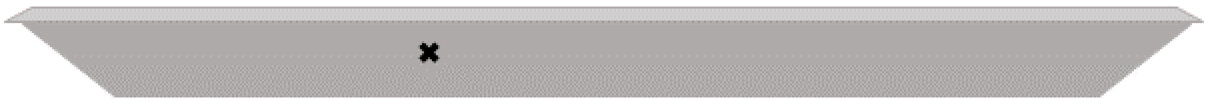

Page Break

Q14
Now try to find as many words as you can in the puzzle below and type them in the field at the bottom of the page. You have 20 seconds!

____

____

____

____

____

Q15 Timing
First Click (1)
Last Click (2)
Page Submit (3)
Click Count (4)

Page Break

Q16
Please click on the image to answer.

After a while, Tom comes back.

Where do you remember he buried the spare house key?

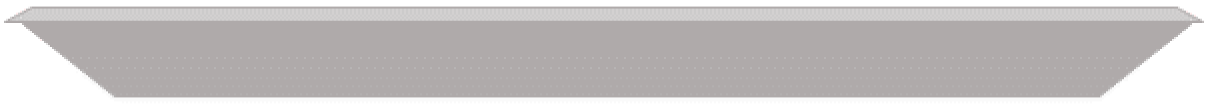

End of Block: Block2

Start of Block: Block3

Q17

Max and Audrey are putting toys in the big toy trunk in the living room. Max has a tennis ball. While Audrey is watching him, Max buries the tennis ball in the trunk here. Max then goes to the kitchen to get a cookie.

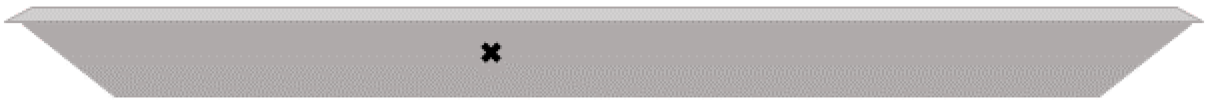

Page Break

Q18

While Max is in the kitchen, Audrey digs the tennis ball out and hides it here. She puts everything else in the trunk back where it was so it looks undisturbed.

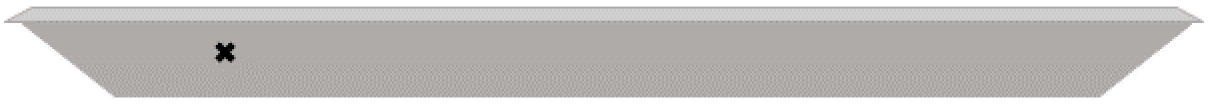

Page Break

Q19
Now try to find as many words as you can in the puzzle below and type them in the field at the bottom of the page. You have 20 seconds!

____

____

____

____

____

Q20 Timing
First Click (1)
Last Click (2)
Page Submit (3)
Click Count (4)

Page Break

Q21
Please click on the image to answer.

After a while, Max comes back.

Where do you remember he buried the tennis ball?

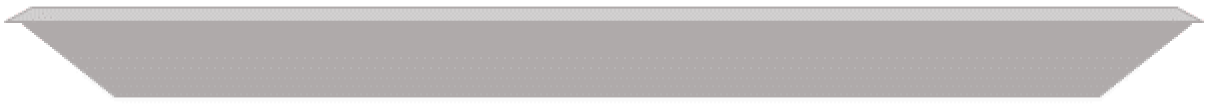

End of Block: Block3

Start of Block: Block4

Q26

Rebecca and Steve are in the restaurant kitchen. Rebecca has the tips jar. While Steve is watching her, Rebecca buries the tips jar in the freezer here. Rebecca then goes outside to smoke a cigarette.

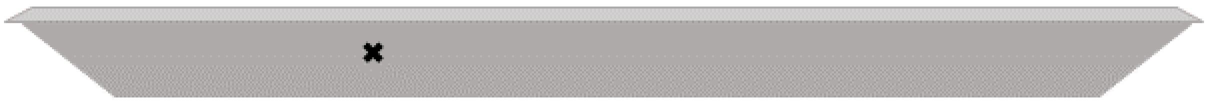

Page Break

Q27

While Rebecca is outside, Steve digs the tips jar out and hides it here. He puts everything else in the freezer back where it was so it looks undisturbed.

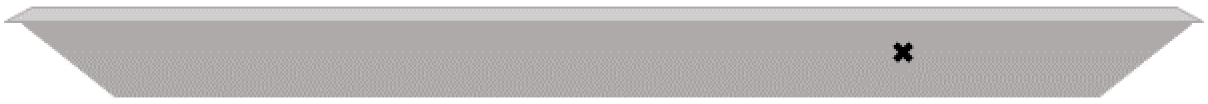

Page Break

Q28
Now try to find as many words as you can in the puzzle below and type them in the field at the bottom of the page. You have 20 seconds!

____

____

____

____

____

Q29 Timing
First Click (1)
Last Click (2)
Page Submit (3)
Click Count (4)

Page Break

Q30
Please click on the image to answer.

After a while, Rebecca comes back.

Where do you remember she buried the tips jar?

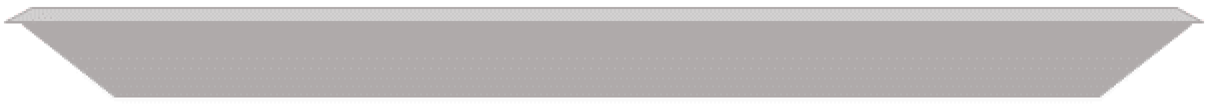

End of Block: Block4

Start of Block: Block5

Q31

Astrid and Luke are in the ball pit. Astrid has a chocolate egg. While Luke is watching her, Astrid buries the chocolate egg in the ball pit here.

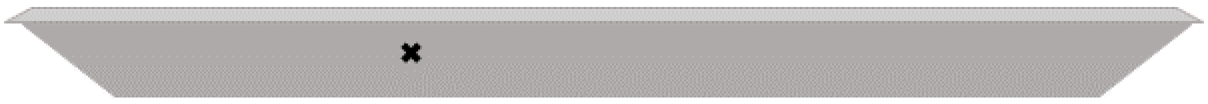

Page Break

Q32

While Luke is still watching her, Astrid digs the chocolate egg out and hides it here. She smooths over the balls so they look undisturbed. Astrid then goes inside to get a drink.

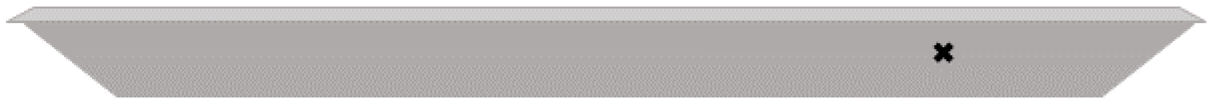

Page Break

Q33
Now try to find as many words as you can in the puzzle below and type them in the field at the bottom of the page. You have 20 seconds!

____

____

____

____

____

Q34 Timing
First Click (1)
Last Click (2)
Page Submit (3)
Click Count (4)

Page Break

Q35
Please click on the image to answer.

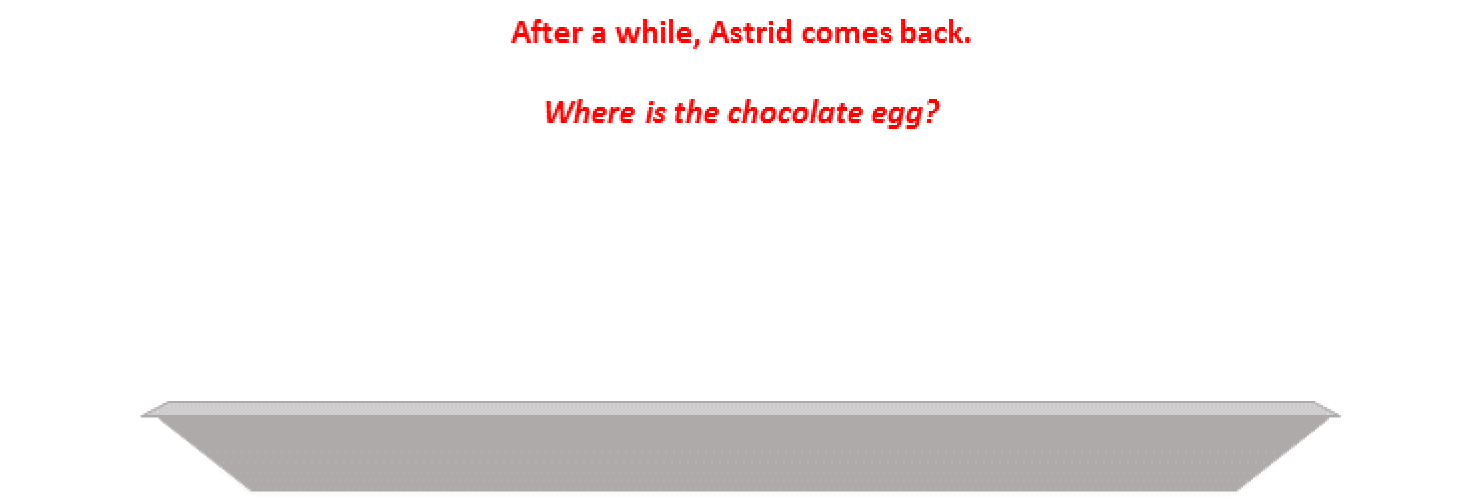

End of Block: Block5

Start of Block: Block6

Q36

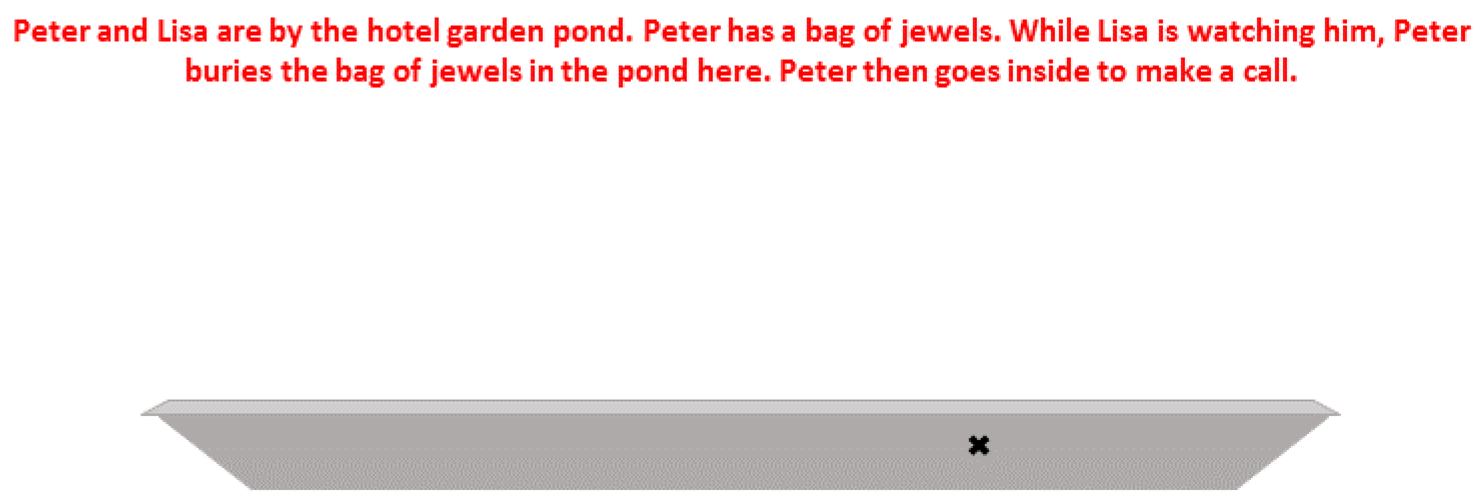

Page Break

Q37

While Peter is inside, Lisa digs the bag of jewels out and hides it here. She watches the surface of the pond until it looks undisturbed.

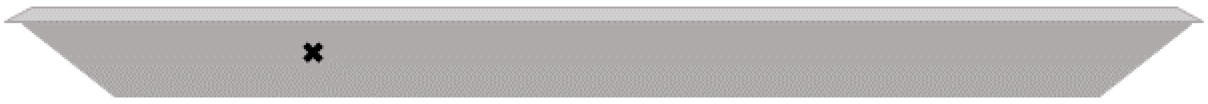

Page Break

Q38
Now try to find as many words as you can in the puzzle below and type them in the field at the bottom of the page. You have 20 seconds!

____

____

____

____

____

Q39 Timing
First Click (1)
Last Click (2)
Page Submit (3)
Click Count (4)

Page Break

Q40

Please click on the image to answer.

After a while, Peter comes back.

Where does Peter remember he buried the bag of jewels?

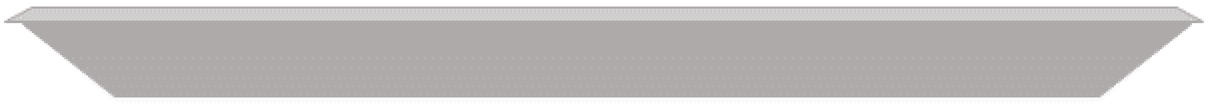

End of Block: Block6

Start of Block: Block7

Q41

Chloe and James are hiking in the snow. Chloe has a bottle of beer. While James is watching her, Chloe buries the bottle of beer in the snow here. Chloe then goes into their tent to check her emails.

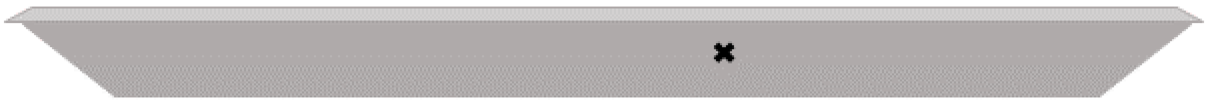

Page Break

Q42

While Chloe is away, James digs the bottle of beer out and hides it here. He smooths over the snow so it looks undisturbed.

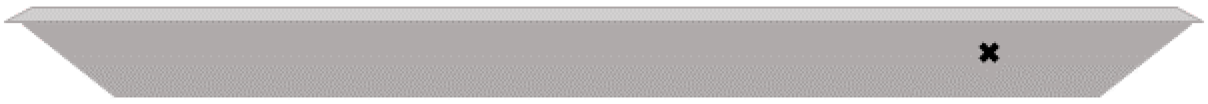

Page Break

Q43
Now try to find as many words as you can in the puzzle below and type them in the field at the bottom of the page. You have 20 seconds!

____

____

____

____

____

Q44 Timing
First Click (1)
Last Click (2)
Page Submit (3)
Click Count (4)

Page Break

Q45
Please click on the image to answer.

After a while, Chloe comes back.

Where does Chloe remember she buried the bottle of beer?

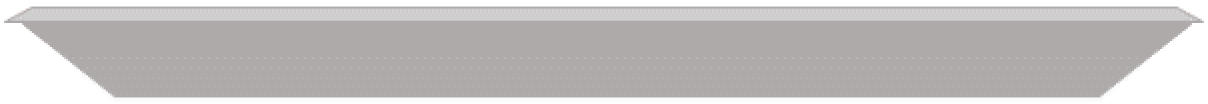

End of Block: Block7

Start of Block: Block8

Q46

Sarah and Alan are on the beach. Sarah has their passports. While Alan is watching her, Sarah buries the passports in the sand here. Sarah then goes to a shop to get some ice cream.

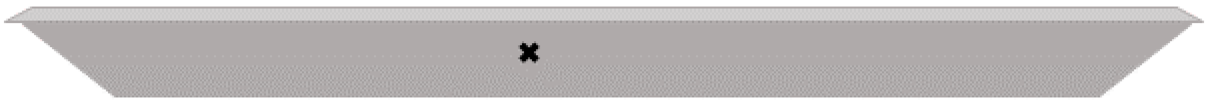

Page Break

Q47

While Sarah is away, Alan digs the passports out and hides them here. He smooths over the sand so it looks undisturbed.

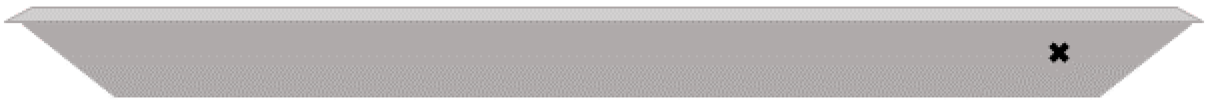

Page Break

Q48
Now try to find as many words as you can in the puzzle below and type them in the field at the bottom of the page. You have 20 seconds!

____

____

____

____

____

Q49 Timing
First Click (1)
Last Click (2)
Page Submit (3)
Click Count (4)

Page Break

Q50
Please click on the image to answer.

After a while, Sarah comes back.

Where does Sarah remember she buried the passports?

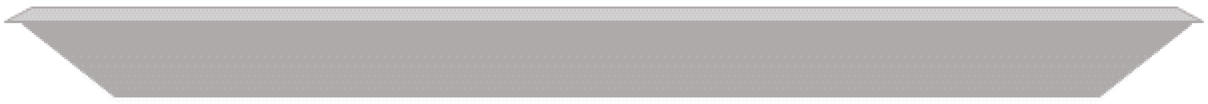

End of Block: Block8

Start of Block: Block9

Q51

John and Alice are in the garden with a planter. John has a flower bulb. While Alice is watching him, John buries the flower bulb in the planter here. John then goes inside to get some water.

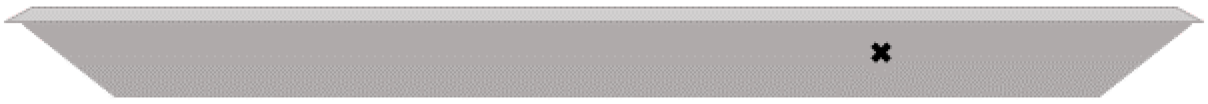

Page Break

Q52

While John is away, Alice digs the flower bulb out and hides it here. She smooths over the soil so it looks undisturbed.

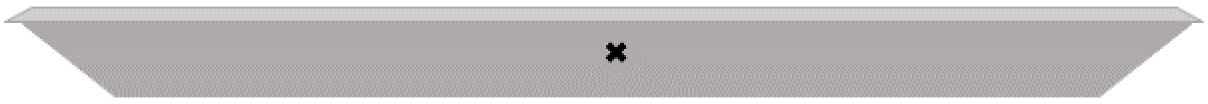

Page Break

Q53
Now try to find as many words as you can in the puzzle below and type them in the field at the bottom of the page. You have 20 seconds!

____

____

____

____

____

Q54 Timing
First Click (1)
Last Click (2)
Page Submit (3)
Click Count (4)

Page Break

Q55
Please click on the image to answer.

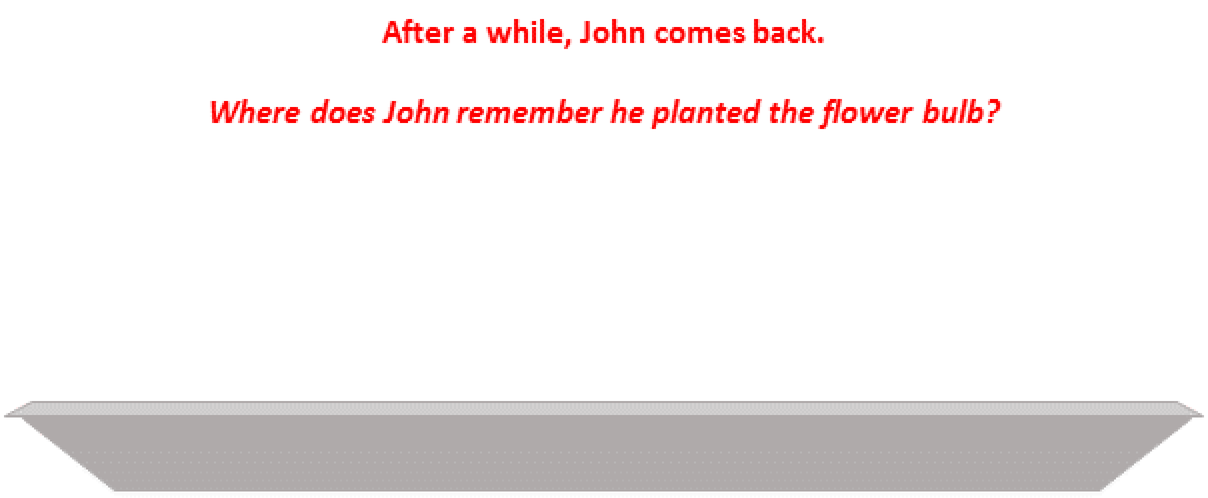

End of Block: Block9

Start of Block: Debrief

Q62 **Participant Debriefing Sheet:**

Thank you for taking part in our study.
What was the study about?
The experiment you took part in is part of a larger study designed to test how people use their own knowledge when having to take into account what others remember or believe. We know that adults tend to expect that other adults know what they themselves know. The critical component of this study was to see whether you were influenced by where you knew an object to be when you had to imagine where someone else remembered or believed it to be.

Page Break

Q62 ***Further Information*** *You are welcome to ask the researchers about the study.*

If you would like to know more about the study you can contact Dr. Steven Samuel (ss2391@cam.ac.uk) for further information.

Page Break

End of Block: Debrief

NEW sandbox: othermem-ownmem

Start of Block: Prolific Info

Q65 Before you start, please:

– maximize your browser window;
– switch off phone/e-mail/music & anything else distracting
- and please enter your Prolific ID [it can be found at the top of this webpage or when going to your account info]:\\\

[To continue to the next question at any point in the survey please use the arrow button at the bottom right of the page]

____

End of Block: Prolific Info

Start of Block: Participant Info

Q54 Before you decide to take part in this study it is important for you to understand why the research is being done and what it will involve. Please take time to read the following information carefully and discuss it with others if you wish. A member of the team can be contacted if there is anything that is not clear or if you would like more information. Take time to decide whether or not you wish to take part.

Page Break

Q55 This study investigates how adults reason about what others want, believe and act. The study fits in with a larger research theme of how children and adults reason about other individuals' mental states and how both children and adults attribute beliefs and desires to other people.

Page Break

Q56 During the study you will be asked to make judgments about what others can see, hear or do. You may also be asked to predict how another person would act based on what they have seen or heard. The experiment will take approximately 10 minutes to complete.

Page Break

Q57 Participation in the study is entirely voluntary and you can withdraw at any point. The results of the study may be written up or presented at conferences. Your participation will remain confidential and if individual data is presented there will be no means of identifying the individuals involved.

Page Break

Q58 If you would like to know more about the study or have any questions you can contact Steven Samuel (ss2391@cam.ac.uk) for further information.

The project has received ethical approval from the Psychology Ethics Committee of the University of Cambridge.

Page Break

End of Block: Participant Info

Start of Block: Consent Page 1/6

Q81 I confirm that I have read and understand the participant information.

◯ Yes (1)

◯ No (2)

Q93 I have had the opportunity to ask questions and had them answered.

◯ Yes (1)

◯ No (2)

Q105 I understand that all personal information will remain confidential and that all efforts will be made to ensure that I cannot be identified (except as might be required by law).

◯ Yes (1)

◯ No (2)

Q117 I agree that data gathered in this study may be stored anonymously and securely, and may be used for future research.

◯ Yes (1)

◯ No (2)

Q129 I understand that my participation is voluntary and that I am free to withdraw at any time without giving a reason.

◯ Yes (1)

◯ No (2)

Q141 I agree to take part in this study.

◯ Yes (1)

◯ No (2)

End of Block: Consent Page 1/6

Start of Block: Consent Page 2/6

Q66 There are two parts to the study.

First you will be asked to click on various locations on your screen (this helps us with calibration).

Second (the main part of the study) you will read some short stories about objects being hidden. Please pay careful attention to the story and the accompanying visual information. After each story you will have 20 seconds to find words in a wordsearch. After each wordsearch you will be asked a question about the story that you have just read. Total experiment time is approximately 10 minutes.

The calibration process is next...

End of Block: Consent Page 2/6

Start of Block: Consent Page 3/6

Start of Block: Consent Page 4/6

Start of Block: Consent Page 5/6

Start of Block: Consent Page 6/6

Start of Block: Start

Q17
Please click right in the centre of the left cross (first) and right in the centre of the right cross (second). Try to be as accurate as possible. If the mouse-controlled cross does not align perfectly with the crosses on the screen (for example it always has to be a fraction to the left or right), please do the same for each cross. Make sure a marker is left on each cross before moving on to the next page. You can try as many times as you like.

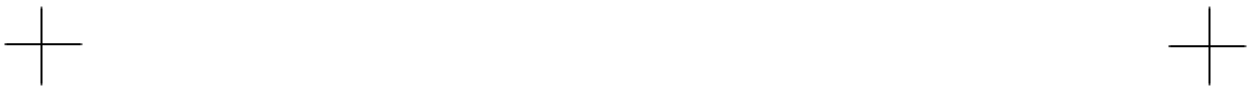

Page Break

Q48

Now please click right in the centre of this left cross and right in the centre of this right cross. Try to be as accurate as possible, and again if the mouse cannot fall exactly in the centre please ensure you do the same for each click. You can try as many times as you like.

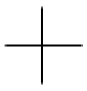

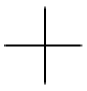

Page Break

Q49
And finally, please do the same for the two crosses on this page.

Page Break

Q47 Browser Meta Info
Browser (1)
Version (2)
Operating System (3)
Screen Resolution (4)
Flash Version (5)
Java Support (6)
User Agent (7)

End of Block: Start

Start of Block: Block1

Q1

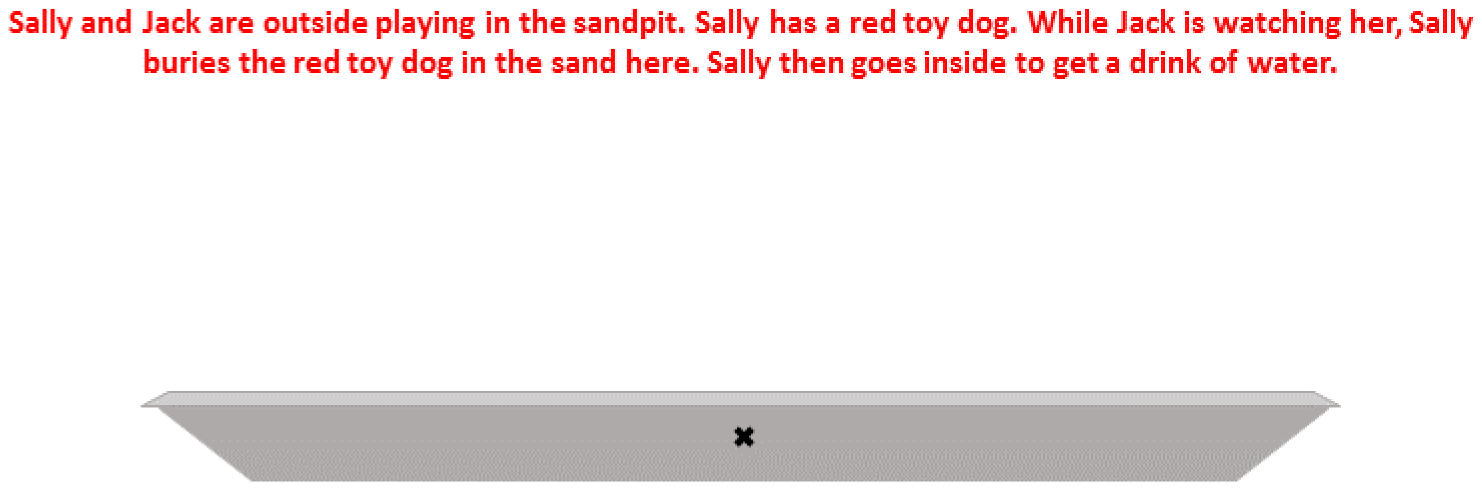

Page Break

Q2

While Sally is inside the house, Jack digs the red toy dog out and hides it here. He smooths over the sand so it looks undisturbed.

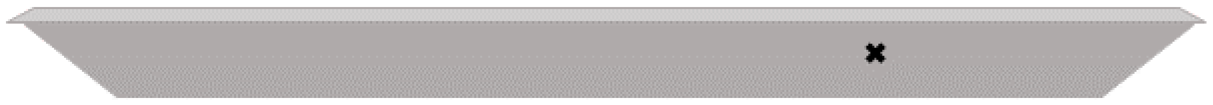

Page Break

Q3
Now try to find as many words as you can in the puzzle below and type them in the field at the bottom of the page. You have 20 seconds!

____

____

____

____

____

Q5 Timing
First Click (1)
Last Click (2)
Page Submit (3)
Click Count (4)

Page Break

Q4
Please click on the image to answer.

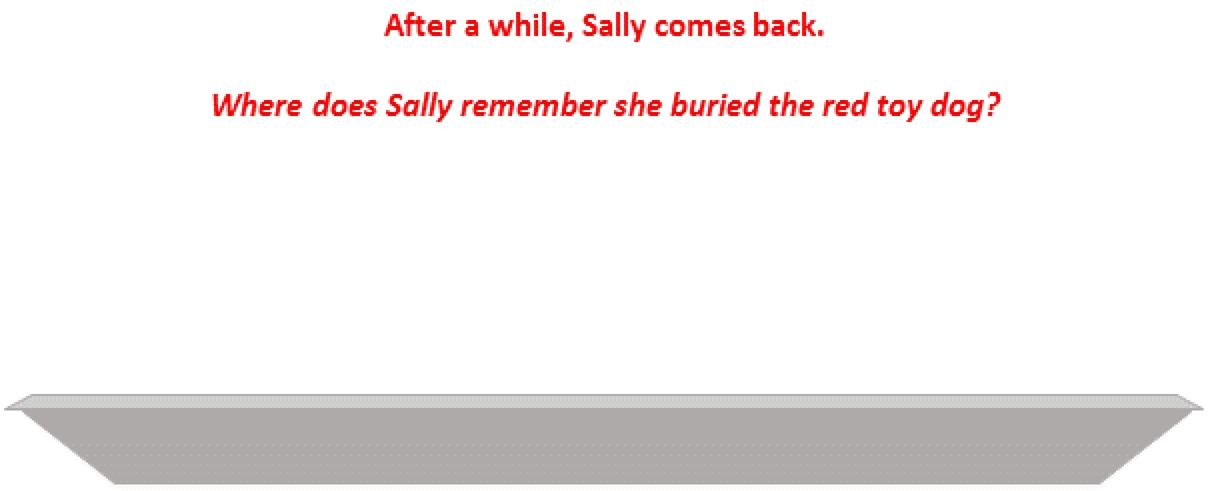

End of Block: Block1

Start of Block: Block2

Q12

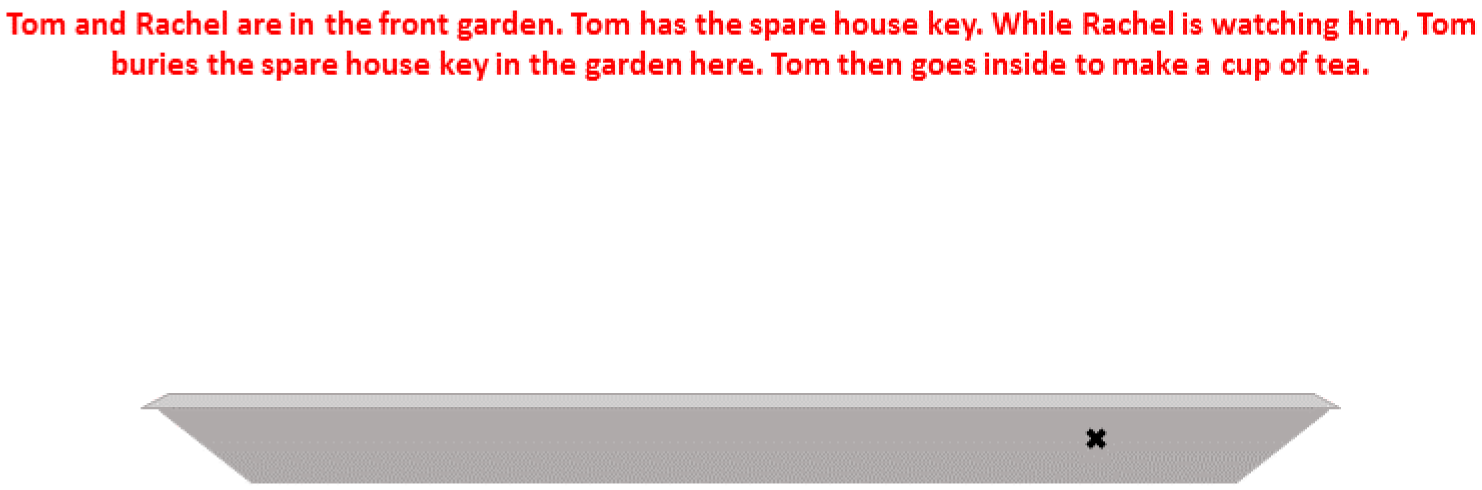

Page Break

Q13

While Tom is inside the house, Rachel digs the spare house key out and hides it here. She smooths over the earth so it looks undisturbed.

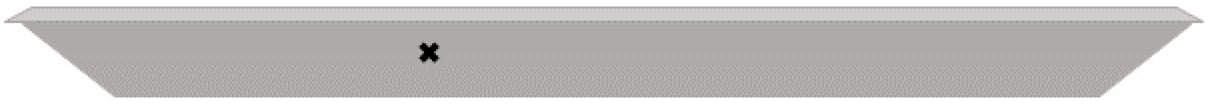

Page Break

Q14
Now try to find as many words as you can in the puzzle below and type them in the field at the bottom of the page. You have 20 seconds!

____

____

____

____

____

Q15 Timing
First Click (1)
Last Click (2)
Page Submit (3)
Click Count (4)

Page Break

Q16
Please click on the image to answer.

After a while, Tom comes back.

Where does Tom remember he buried the spare house key?

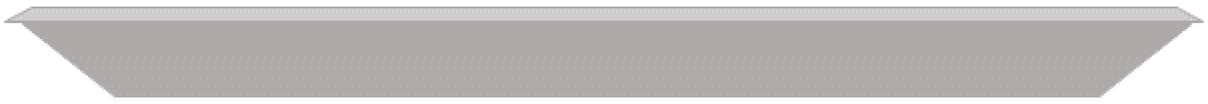

End of Block: Block2

Start of Block: Block3

Q17

Max and Audrey are putting toys in the big toy trunk in the living room. Max has a tennis ball. While Audrey is watching him, Max buries the tennis ball in the trunk here. Max then goes to the kitchen to get a cookie.

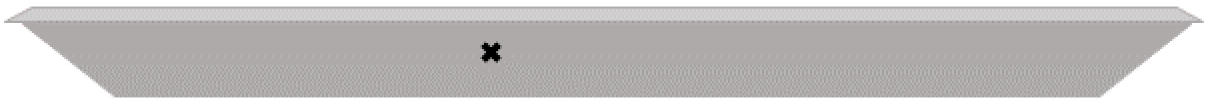

Page Break

Q18

While Max is in the kitchen, Audrey digs the tennis ball out and hides it here. She puts everything else in the trunk back where it was so it looks undisturbed.

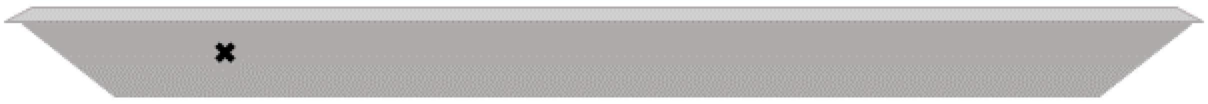

Page Break

Q19
Now try to find as many words as you can in the puzzle below and type them in the field at the bottom of the page. You have 20 seconds!

____

____

____

____

____

Q20 Timing
First Click (1)
Last Click (2)
Page Submit (3)
Click Count (4)

Page Break

Q21
Please click on the image to answer.

After a while, Max comes back.

Where does Max remember he buried the tennis ball?

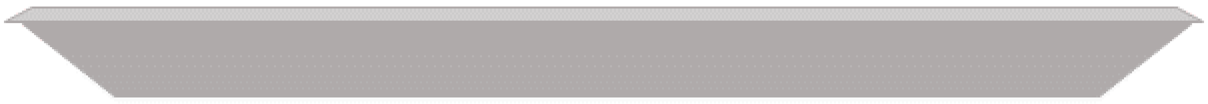

End of Block: Block3

Start of Block: Block4

Q26

Rebecca and Steve are in the restaurant kitchen. Rebecca has the tips jar. While Steve is watching her, Rebecca buries the tips jar in the freezer here. Rebecca then goes outside to smoke a cigarette.

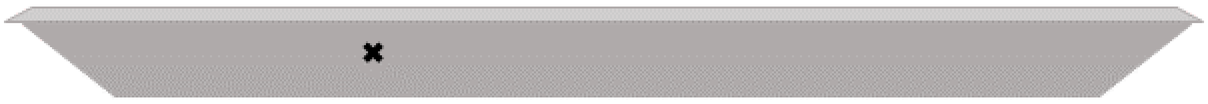

Page Break

Q27

While Rebecca is outside, Steve digs the tips jar out and hides it here. He puts everything else in the freezer back where it was so it looks undisturbed.

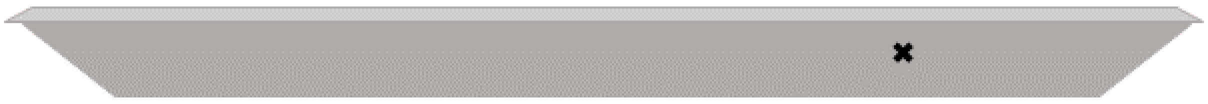

Page Break

Q28
Now try to find as many words as you can in the puzzle below and type them in the field at the bottom of the page. You have 20 seconds!

____

____

____

____

____

Q29 Timing
First Click (1)
Last Click (2)
Page Submit (3)
Click Count (4)

Page Break

Q30
Please click on the image to answer.

After a while, Rebecca comes back.

Where does Rebecca remember she buried the tips jar?

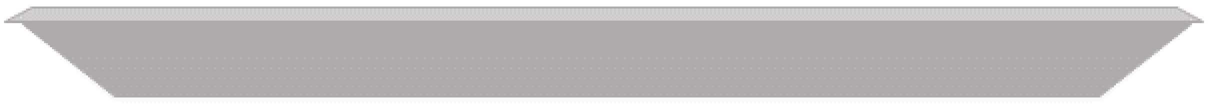

End of Block: Block4

Start of Block: Block5

Q31

Astrid and Luke are in the ball pit. Astrid has a chocolate egg. While Luke is watching her, Astrid buries the chocolate egg in the ball pit here.

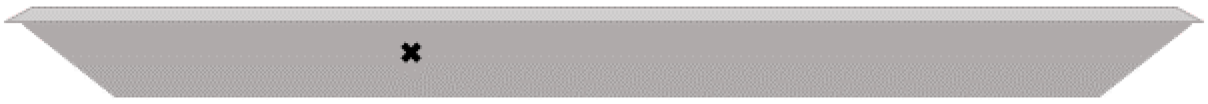

Page Break

Q32

While Luke is still watching her, Astrid digs the chocolate egg out and hides it here. She smooths over the balls so they look undisturbed. Astrid then goes inside to get a drink.

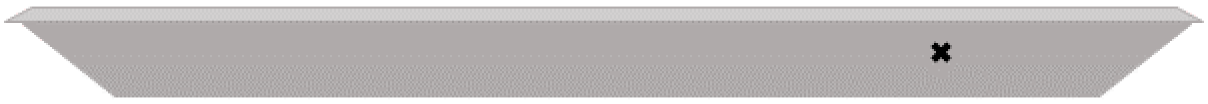

Page Break

Q33
Now try to find as many words as you can in the puzzle below and type them in the field at the bottom of the page. You have 20 seconds!

____

____

____

____

____

Q34 Timing
First Click (1)
Last Click (2)
Page Submit (3)
Click Count (4)

Page Break

Q35
Please click on the image to answer.

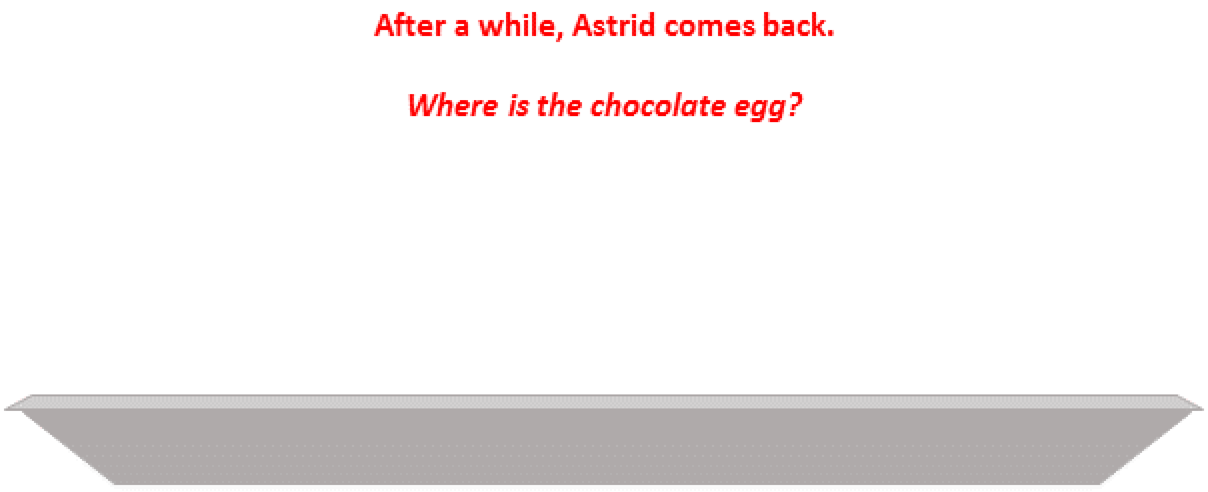

End of Block: Block5

Start of Block: Block6

Q36

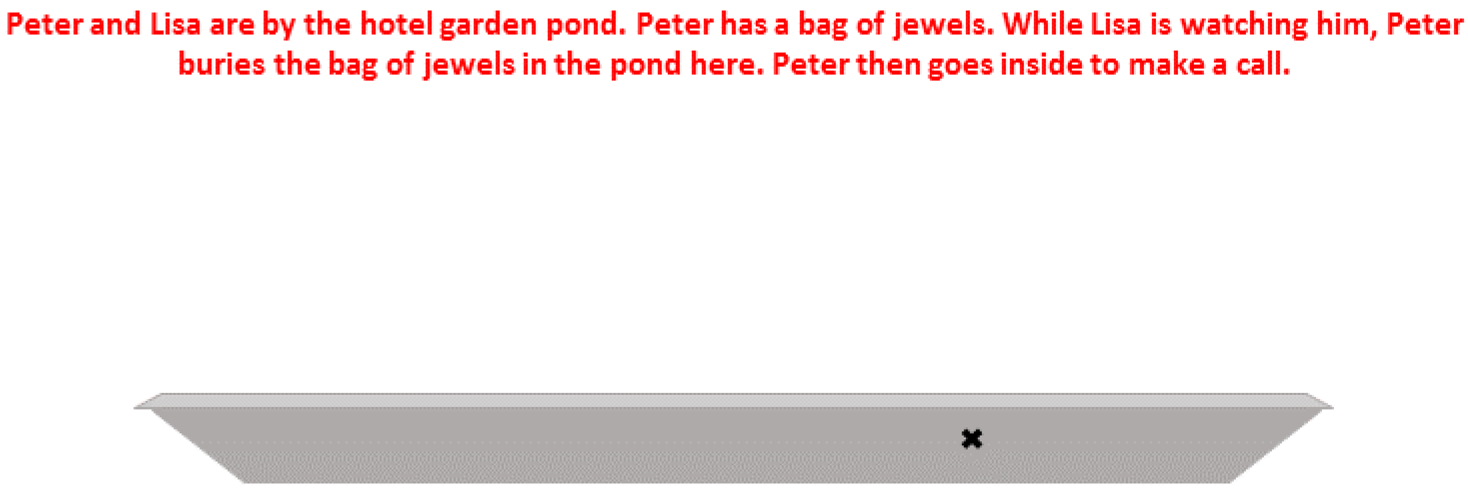

Page Break

Q37

While Peter is inside, Lisa digs the bag of jewels out and hides it here. She watches the surface of the pond until it looks undisturbed.

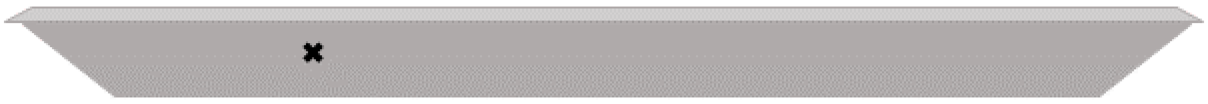

Page Break

Q38
Now try to find as many words as you can in the puzzle below and type them in the field at the bottom of the page. You have 20 seconds!

____

____

____

____

____

Q39 Timing
First Click (1)
Last Click (2)
Page Submit (3)
Click Count (4)

Page Break

Q40
Please click on the image to answer.

After a while, Peter comes back.

Where do you remember he buried the bag of jewels?

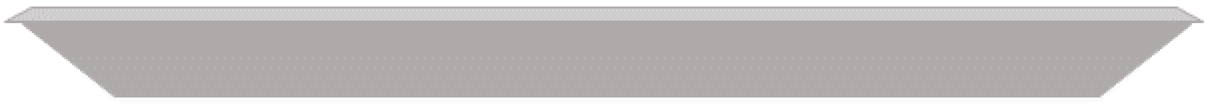

End of Block: Block6

Start of Block: Block7

Q41

Chloe and James are hiking in the snow. Chloe has a bottle of beer. While James is watching her, Chloe buries the bottle of beer in the snow here. Chloe then goes into their tent to check her emails.

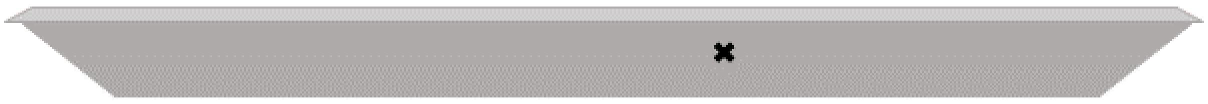

Page Break

Q42

While Chloe is away, James digs the bottle of beer out and hides it here. He smooths over the snow so it looks undisturbed.

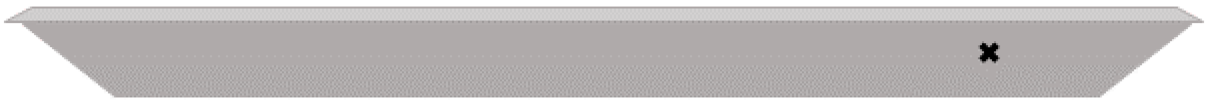

Page Break

Q43
Now try to find as many words as you can in the puzzle below and type them in the field at the bottom of the page. You have 20 seconds!

____

____

____

____

____

Q44 Timing
First Click (1)
Last Click (2)
Page Submit (3)
Click Count (4)

Page Break

Q45
Please click on the image to answer.

After a while, Chloe comes back.

Where do you remember she buried the bottle of beer?

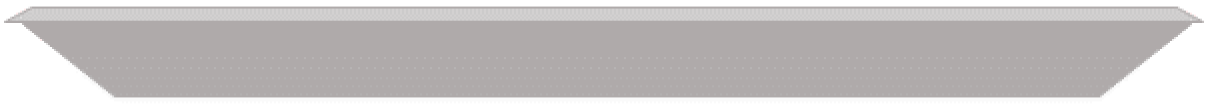

End of Block: Block7

Start of Block: Block8

Q46

Sarah and Alan are on the beach. Sarah has their passports. While Alan is watching her, Sarah buries the passports in the sand here. Sarah then goes to a shop to get some ice cream.

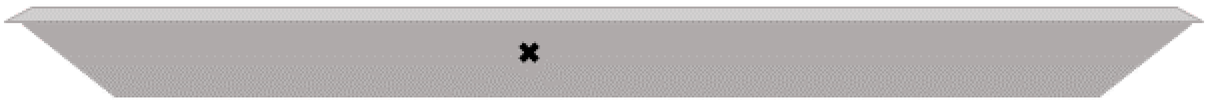

Page Break

Q47

While Sarah is away, Alan digs the passports out and hides them here. He smooths over the sand so it looks undisturbed.

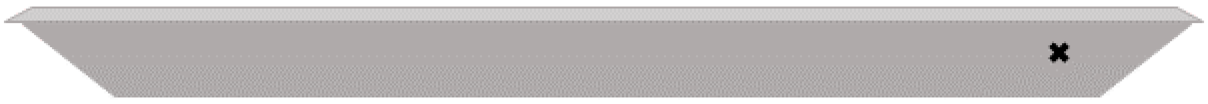

Page Break

Q48
Now try to find as many words as you can in the puzzle below and type them in the field at the bottom of the page. You have 20 seconds!

____

____

____

____

____

Q49 Timing
First Click (1)
Last Click (2)
Page Submit (3)
Click Count (4)

Page Break

Q50
Please click on the image to answer.

After a while, Sarah comes back.

Where do you remember she buried the passports?

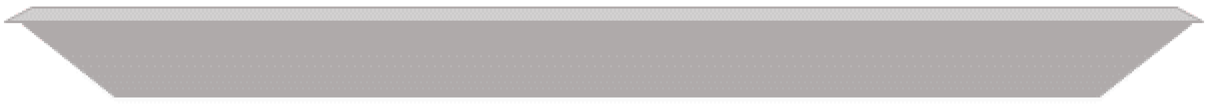

End of Block: Block8

Start of Block: Block9

Q51

John and Alice are in the garden with a planter. John has a flower bulb. While Alice is watching him, John buries the flower bulb in the planter here. John then goes inside to get some water.

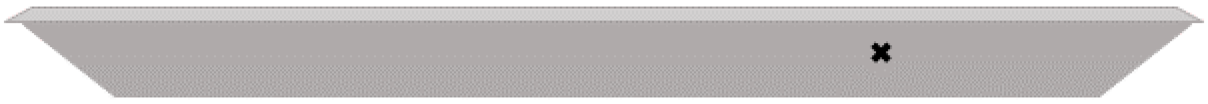

Page Break

Q52

While John is away, Alice digs the flower bulb out and hides it here. She smooths over the soil so it looks undisturbed.

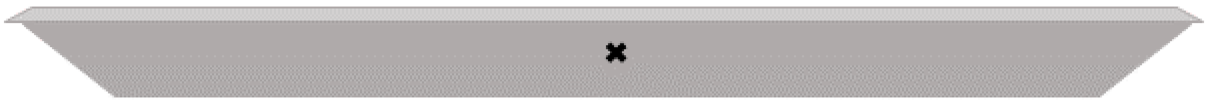

Page Break

Q53
Now try to find as many words as you can in the puzzle below and type them in the field at the bottom of the page. You have 20 seconds!

____

____

____

____

____

Q54 Timing
First Click (1)
Last Click (2)
Page Submit (3)
Click Count (4)

Page Break

Q55
Please click on the image to answer.

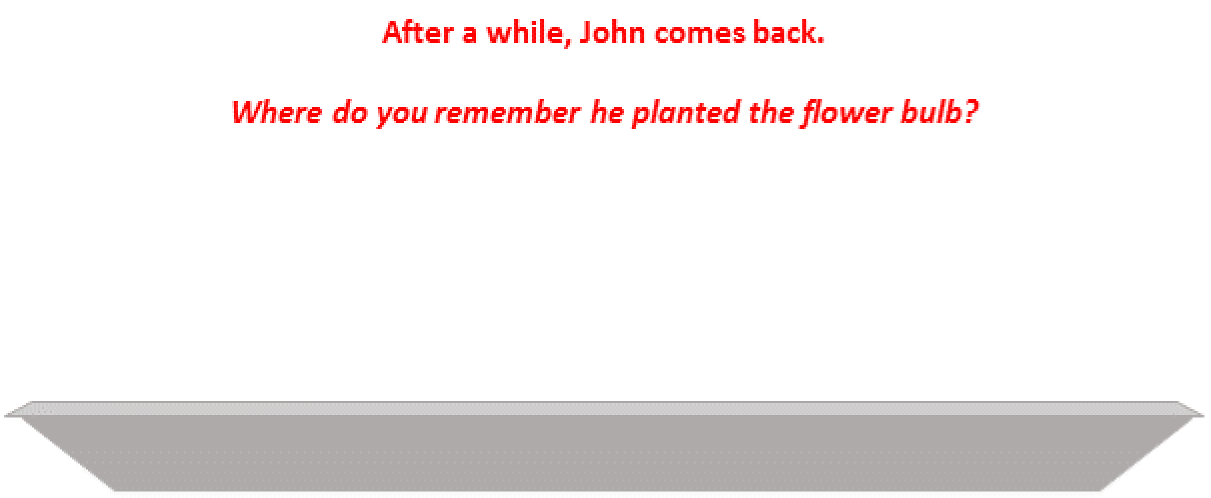

End of Block: Block9

Start of Block: Debrief

Q62 **Participant Debriefing Sheet:**

Thank you for taking part in our study.
What was the study about?
The experiment you took part in is part of a larger study designed to test how people use their own knowledge when having to take into account what others remember or believe. We know that adults tend to expect that other adults know what they themselves know. The critical component of this study was to see whether you were influenced by where you knew an object to be when you had to imagine where someone else remembered or believed it to be.

Page Break

Q62 ***Further Information*** *You are welcome to ask the researchers about the study.*

If you would like to know more about the study you can contact Dr. Steven Samuel (ss2391@cam.ac.uk) for further information.

Page Break

End of Block: Debrief

NEW sandbox: ownmem-otherbelief

Start of Block: Prolific Info

Q65 Before you start, please:

– maximize your browser window;
– switch off phone/e-mail/music & anything else distracting
- and please enter your Prolific ID [it can be found at the top of this webpage or when going to your account info]:\\\

[To continue to the next question at any point in the survey please use the arrow button at the bottom right of the page]

____

End of Block: Prolific Info

Start of Block: Participant Info

Q54 Before you decide to take part in this study it is important for you to understand why the research is being done and what it will involve. Please take time to read the following information carefully and discuss it with others if you wish. A member of the team can be contacted if there is anything that is not clear or if you would like more information. Take time to decide whether or not you wish to take part.

Page Break

Q55 This study investigates how adults reason about what others want, believe and act. The study fits in with a larger research theme of how children and adults reason about other individuals' mental states and how both children and adults attribute beliefs and desires to other people.

Page Break

Q56 During the study you will be asked to make judgments about what others can see, hear or do. You may also be asked to predict how another person would act based on what they have seen or heard. The experiment will take approximately 10 minutes to complete.

Page Break

Q57 Participation in the study is entirely voluntary and you can withdraw at any point. The results of the study may be written up or presented at conferences. Your participation will remain confidential and if individual data is presented there will be no means of identifying the individuals involved.

Page Break

Q58 If you would like to know more about the study or have any questions you can contact Steven Samuel (ss2391@cam.ac.uk) for further information.

The project has received ethical approval from the Psychology Ethics Committee of the University of Cambridge.

Page Break

End of Block: Participant Info

Start of Block: Consent Page 1/6

Q81 I confirm that I have read and understand the participant information.

- Yes (1)
- No (2)

Q93 I have had the opportunity to ask questions and had them answered.

- Yes (1)
- No (2)

Q105 I understand that all personal information will remain confidential and that all efforts will be made to ensure that I cannot be identified (except as might be required by law).

- Yes (1)
- No (2)

Q117 I agree that data gathered in this study may be stored anonymously and securely, and may be used for future research.

- Yes (1)
- No (2)

Q129 I understand that my participation is voluntary and that I am free to withdraw at any time without giving a reason.

- Yes (1)
- No (2)

Q141 I agree to take part in this study.

- Yes (1)
- No (2)

End of Block: Consent Page 1/6

Start of Block: Consent Page 2/6

Q66 There are two parts to the study.

First you will be asked to click on various locations on your screen (this helps us with calibration).

Second (the main part of the study) you will read some short stories about objects being hidden. Please pay careful attention to the story and the accompanying visual information. After each story you will have 20 seconds to find words in a wordsearch. After each wordsearch you will be asked a question about the story that you have just read. Total experiment time is approximately 10 minutes.

The calibration process is next...

End of Block: Consent Page 2/6

Start of Block: Consent Page 3/6

Start of Block: Consent Page 4/6

Start of Block: Consent Page 5/6

Start of Block: Consent Page 6/6

Start of Block: Start

Q17
Please click right in the centre of the left cross (first) and right in the centre of the right cross (second). Try to be as accurate as possible. If the mouse-controlled cross does not align perfectly with the crosses on the screen (for example it always has to be a fraction to the left or right), please do the same for each cross. Make sure a marker is left on each cross before moving on to the next page. You can try as many times as you like.

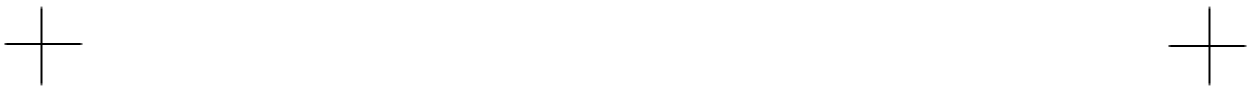

Page Break

Q48
Now please click right in the centre of this left cross and right in the centre of this right cross. Try to be as accurate as possible, and again if the mouse cannot fall exactly in the centre please ensure you do the same for each click. You can try as many times as you like.

Page Break

Q49
And finally, please do the same for the two crosses on this page.

Page Break

Q47 Browser Meta Info
Browser (1)
Version (2)
Operating System (3)
Screen Resolution (4)
Flash Version (5)
Java Support (6)
User Agent (7)

End of Block: Start

Start of Block: Block1

Q1

Sally and Jack are outside playing in the sandpit. Sally has a red toy dog. While Jack is watching her, Sally buries the red toy dog in the sand here. Sally then goes inside to get a drink of water.

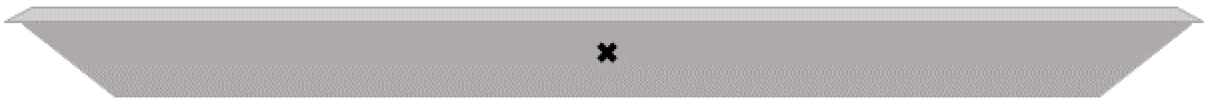

Page Break

Q2

While Sally is inside the house, Jack digs the red toy dog out and hides it here. He smooths over the sand so it looks undisturbed.

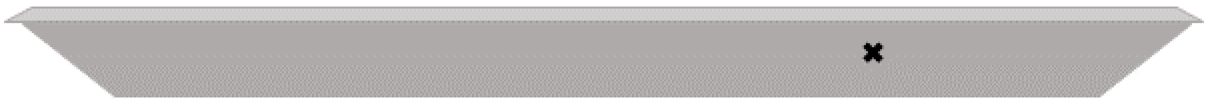

Page Break

Q3
Now try to find as many words as you can in the puzzle below and type them in the field at the bottom of the page. You have 20 seconds!

____

____

____

____

____

Q5 Timing
First Click (1)
Last Click (2)
Page Submit (3)
Click Count (4)

Page Break

Q4
Please click on the image to answer.

After a while, Sally comes back.

Where do you remember she buried the red toy dog?

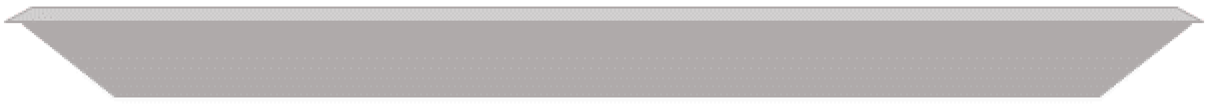

End of Block: Block1

Start of Block: Block2

Q12

Tom and Rachel are in the front garden. Tom has the spare house key. While Rachel is watching him, Tom buries the spare house key in the garden here. Tom then goes inside to make a cup of tea.

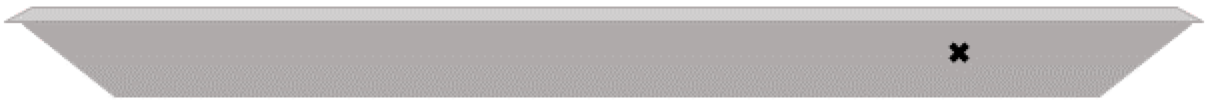

Page Break

Q13

While Tom is inside the house, Rachel digs the spare house key out and hides it here. She smooths over the earth so it looks undisturbed.

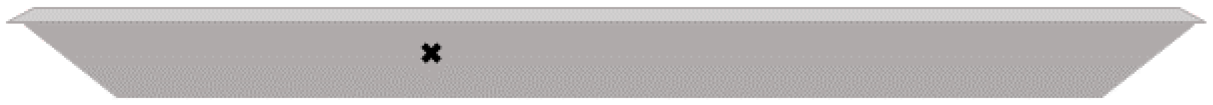

Page Break

Q14
Now try to find as many words as you can in the puzzle below and type them in the field at the bottom of the page. You have 20 seconds!

____

____

____

____

____

Q15 Timing
First Click (1)
Last Click (2)
Page Submit (3)
Click Count (4)

Page Break

Q16
Please click on the image to answer.

After a while, Tom comes back.

Where do you remember he buried the spare house key?

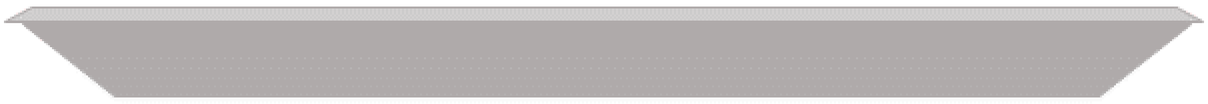

End of Block: Block2

Start of Block: Block3

Q17

Max and Audrey are putting toys in the big toy trunk in the living room. Max has a tennis ball. While Audrey is watching him, Max buries the tennis ball in the trunk here. Max then goes to the kitchen to get a cookie.

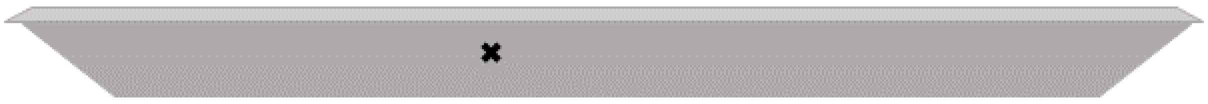

Page Break

Q18

While Max is in the kitchen, Audrey digs the tennis ball out and hides it here. She puts everything else in the trunk back where it was so it looks undisturbed.

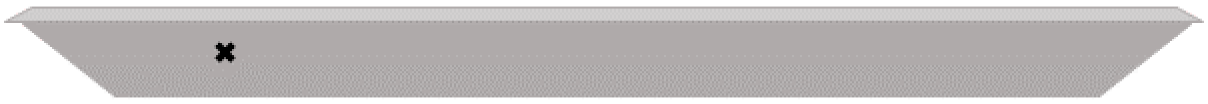

Page Break

Q19
Now try to find as many words as you can in the puzzle below and type them in the field at the bottom of the page. You have 20 seconds!

____

____

____

____

____

Q20 Timing
First Click (1)
Last Click (2)
Page Submit (3)
Click Count (4)

Page Break

Q21
Please click on the image to answer.

After a while, Max comes back.

Where do you remember he buried the tennis ball?

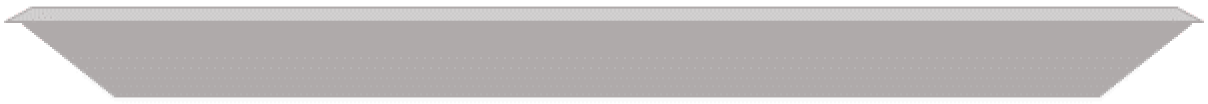

End of Block: Block3

Start of Block: Block4

Q26

Rebecca and Steve are in the restaurant kitchen. Rebecca has the tips jar. While Steve is watching her, Rebecca buries the tips jar in the freezer here. Rebecca then goes outside to smoke a cigarette.

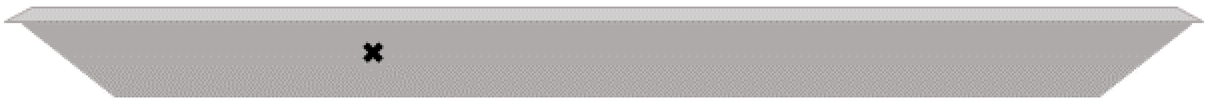

Page Break

Q27

While Rebecca is outside, Steve digs the tips jar out and hides it here. He puts everything else in the freezer back where it was so it looks undisturbed.

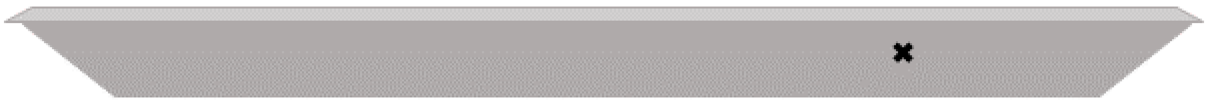

Page Break

Q28
Now try to find as many words as you can in the puzzle below and type them in the field at the bottom of the page. You have 20 seconds!

____

____

____

____

____

Q29 Timing
First Click (1)
Last Click (2)
Page Submit (3)
Click Count (4)

Page Break

Q30
Please click on the image to answer.

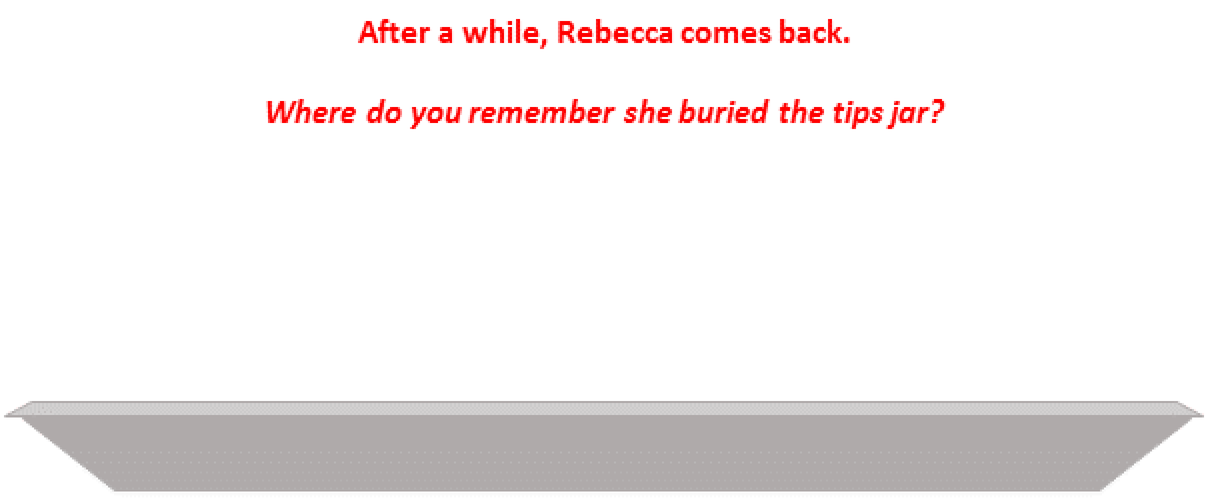

End of Block: Block4

Start of Block: Block5

Q31

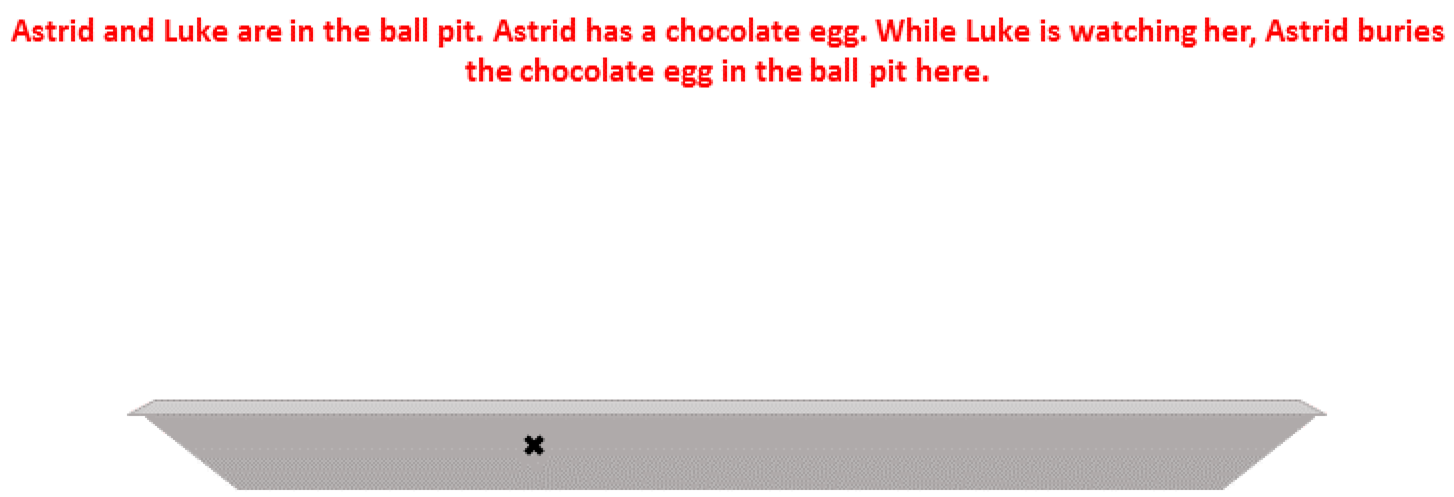

Page Break

Q32

While Luke is still watching her, Astrid digs the chocolate egg out and hides it here. She smooths over the balls so they look undisturbed. Astrid then goes inside to get a drink.

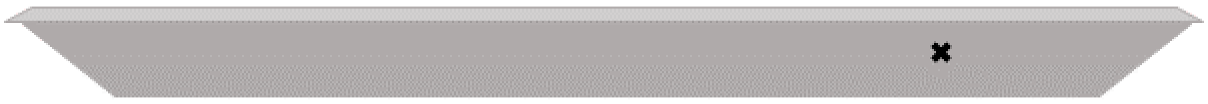

Page Break

Q33
Now try to find as many words as you can in the puzzle below and type them in the field at the bottom of the page. You have 20 seconds!

____

____

____

____

____

Q34 Timing
First Click (1)
Last Click (2)
Page Submit (3)
Click Count (4)

Page Break

Q35
Please click on the image to answer.

After a while, Astrid comes back.

Where is the chocolate egg?

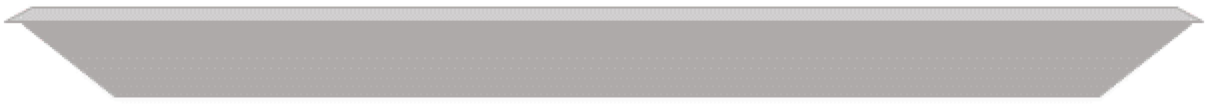

End of Block: Block5

Start of Block: Block6

Q36

Peter and Lisa are by the hotel garden pond. Peter has a bag of jewels. While Lisa is watching him, Peter buries the bag of jewels in the pond here. Peter then goes inside to make a call.

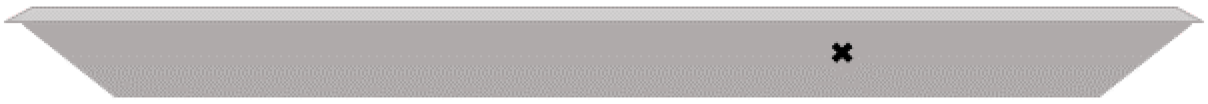

Page Break

Q37

While Peter is inside, Lisa digs the bag of jewels out and hides it here. She watches the surface of the pond until it looks undisturbed.

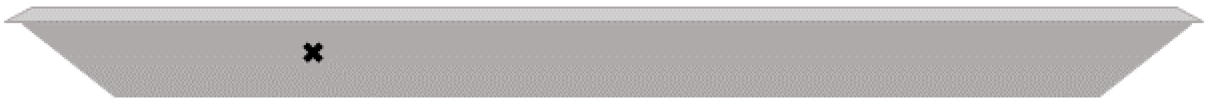

Page Break

Q38
Now try to find as many words as you can in the puzzle below and type them in the field at the bottom of the page. You have 20 seconds!

____

____

____

____

____

Q39 Timing
First Click (1)
Last Click (2)
Page Submit (3)
Click Count (4)

Page Break

Q40
Please click on the image to answer.

After a while, Peter comes back.

Where does Peter believe the bag of jewels to be?

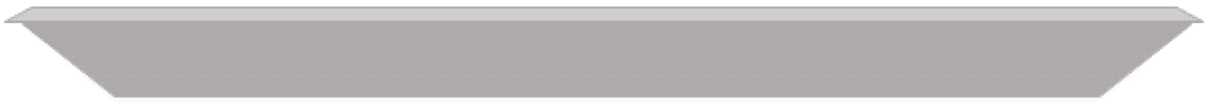

End of Block: Block6

Start of Block: Block7

Q41

Chloe and James are hiking in the snow. Chloe has a bottle of beer. While James is watching her, Chloe buries the bottle of beer in the snow here. Chloe then goes into their tent to check her emails.

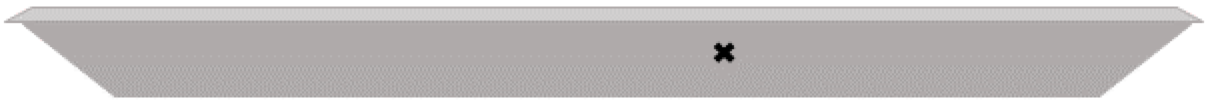

Page Break

Q42

While Chloe is away, James digs the bottle of beer out and hides it here. He smooths over the snow so it looks undisturbed.

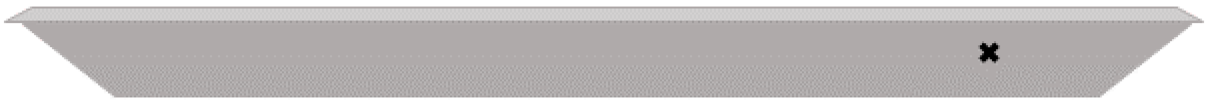

Page Break

Q43
Now try to find as many words as you can in the puzzle below and type them in the field at the bottom of the page. You have 20 seconds!

____

____

____

____

____

Q44 Timing
First Click (1)
Last Click (2)
Page Submit (3)
Click Count (4)

Page Break

Q45
Please click on the image to answer.

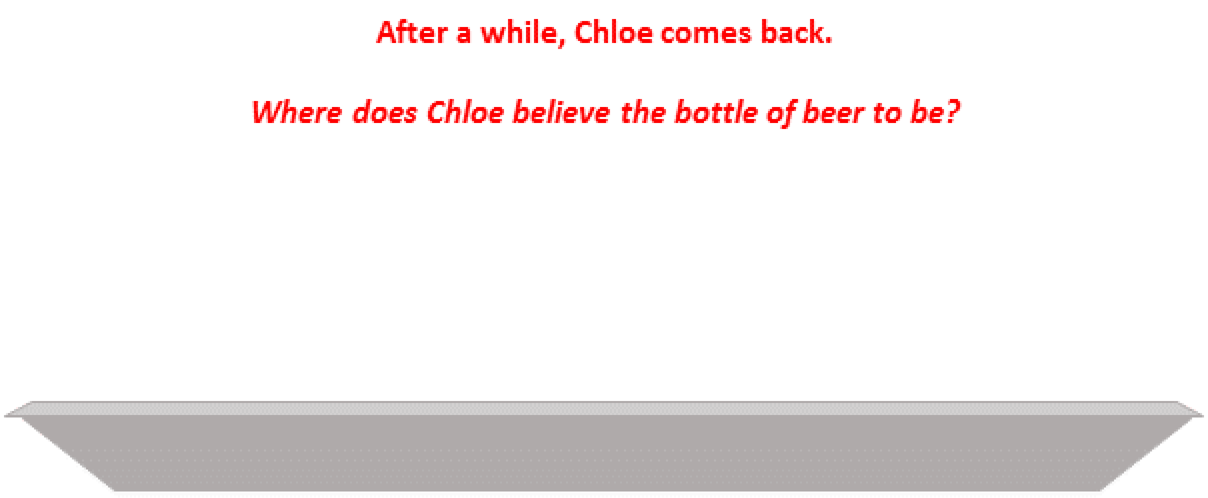

End of Block: Block7

Start of Block: Block8

Q46

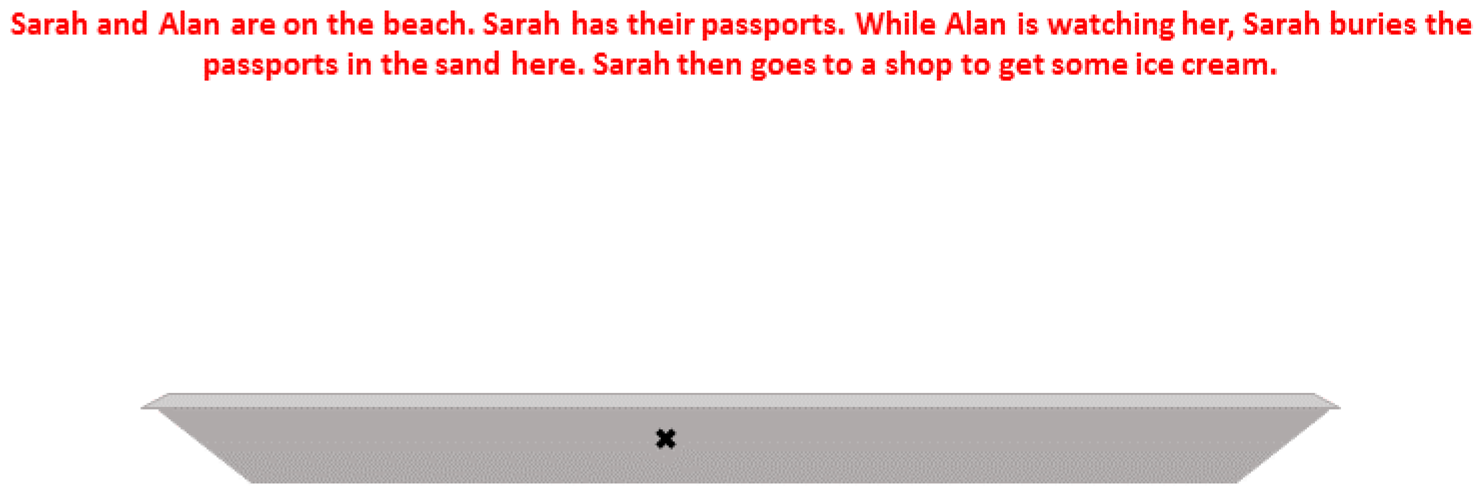

Page Break

Q47

While Sarah is away, Alan digs the passports out and hides them here. He smooths over the sand so it looks undisturbed.

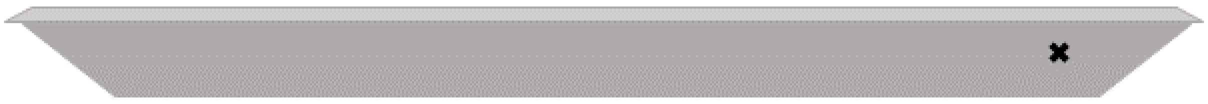

Page Break

Q48
Now try to find as many words as you can in the puzzle below and type them in the field at the bottom of the page. You have 20 seconds!

____

____

____

____

____

Q49 Timing
First Click (1)
Last Click (2)
Page Submit (3)
Click Count (4)

Page Break

Q50
Please click on the image to answer.

After a while, Sarah comes back.

Where does Sarah believe the passports to be?

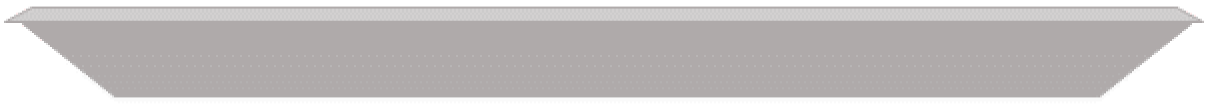

End of Block: Block8

Start of Block: Block9

Q51

John and Alice are in the garden with a planter. John has a flower bulb. While Alice is watching him, John buries the flower bulb in the planter here. John then goes inside to get some water.

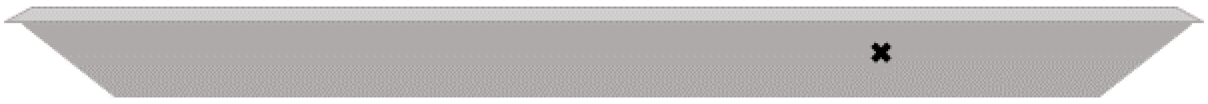

Page Break

Q52

While John is away, Alice digs the flower bulb out and hides it here. She smooths over the soil so it looks undisturbed.

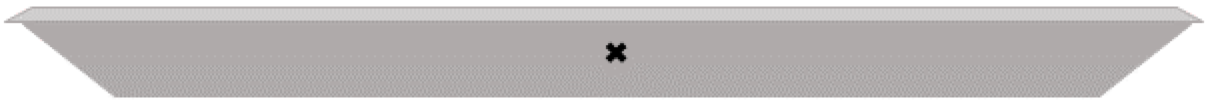

Page Break

Q53
Now try to find as many words as you can in the puzzle below and type them in the field at the bottom of the page. You have 20 seconds!

____

____

____

____

____

Q54 Timing
First Click (1)
Last Click (2)
Page Submit (3)
Click Count (4)

Page Break

Q55
Please click on the image to answer.

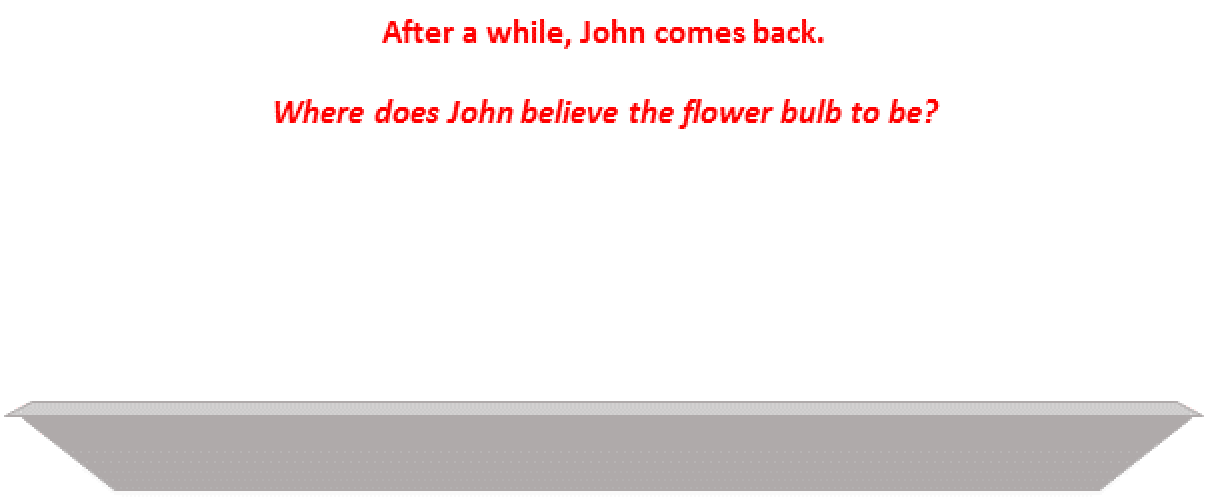

End of Block: Block9

Start of Block: Debrief

Q62 **Participant Debriefing Sheet:**

Thank you for taking part in our study.
What was the study about?
The experiment you took part in is part of a larger study designed to test how people use their own knowledge when having to take into account what others remember or believe. We know that adults tend to expect that other adults know what they themselves know. The critical component of this study was to see whether you were influenced by where you knew an object to be when you had to imagine where someone else remembered or believed it to be.

Page Break

Q62 ***Further Information*** *You are welcome to ask the researchers about the study.*

If you would like to know more about the study you can contact Dr. Steven Samuel (ss2391@cam.ac.uk) for further information.

Page Break

End of Block: Debrief

NEW sandbox: otherbelief-ownmem

Start of Block: Prolific Info

Q65 Before you start, please:

– maximize your browser window;
– switch off phone/e-mail/music & anything else distracting
- and please enter your Prolific ID [it can be found at the top of this webpage or when going to your account info]:\\\

[To continue to the next question at any point in the survey please use the arrow button at the bottom right of the page]

____

End of Block: Prolific Info

Start of Block: Participant Info

Q54 Before you decide to take part in this study it is important for you to understand why the research is being done and what it will involve. Please take time to read the following information carefully and discuss it with others if you wish. A member of the team can be contacted if there is anything that is not clear or if you would like more information. Take time to decide whether or not you wish to take part.

Page Break

Q55 This study investigates how adults reason about what others want, believe and act. The study fits in with a larger research theme of how children and adults reason about other individuals' mental states and how both children and adults attribute beliefs and desires to other people.

Page Break

Q56 During the study you will be asked to make judgments about what others can see, hear or do. You may also be asked to predict how another person would act based on what they have seen or heard. The experiment will take approximately 10 minutes to complete.

Page Break

Q57 Participation in the study is entirely voluntary and you can withdraw at any point. The results of the study may be written up or presented at conferences. Your participation will remain confidential and if individual data is presented there will be no means of identifying the individuals involved.

Page Break

Q58 If you would like to know more about the study or have any questions you can contact Steven Samuel (ss2391@cam.ac.uk) for further information.

The project has received ethical approval from the Psychology Ethics Committee of the University of Cambridge.

Page Break

End of Block: Participant Info

Start of Block: Consent Page 1/6

Q81 I confirm that I have read and understand the participant information.

○ Yes (1)

○ No (2)

Q93 I have had the opportunity to ask questions and had them answered.

○ Yes (1)

○ No (2)

Q105 I understand that all personal information will remain confidential and that all efforts will be made to ensure that I cannot be identified (except as might be required by law).

○ Yes (1)

○ No (2)

Q117 I agree that data gathered in this study may be stored anonymously and securely, and may be used for future research.

○ Yes (1)

○ No (2)

Q129 I understand that my participation is voluntary and that I am free to withdraw at any time without giving a reason.

○ Yes (1)

○ No (2)

Q141 I agree to take part in this study.

○ Yes (1)

○ No (2)

End of Block: Consent Page 1/6

Start of Block: Consent Page 2/6

Q66 There are two parts to the study.

First you will be asked to click on various locations on your screen (this helps us with calibration).

Second (the main part of the study) you will read some short stories about objects being hidden. Please pay careful attention to the story and the accompanying visual information. After each story you will have 20 seconds to find words in a wordsearch. After each wordsearch you will be asked a question about the story that you have just read. Total experiment time is approximately 10 minutes.

The calibration process is next...

End of Block: Consent Page 2/6

Start of Block: Consent Page 3/6

Start of Block: Consent Page 4/6

Start of Block: Consent Page 5/6

Start of Block: Consent Page 6/6

Start of Block: Start

Q17
Please click right in the centre of the left cross (first) and right in the centre of the right cross (second). Try to be as accurate as possible. If the mouse-controlled cross does not align perfectly with the crosses on the screen (for example it always has to be a fraction to the left or right), please do the same for each cross. Make sure a marker is left on each cross before moving on to the next page. You can try as many times as you like.

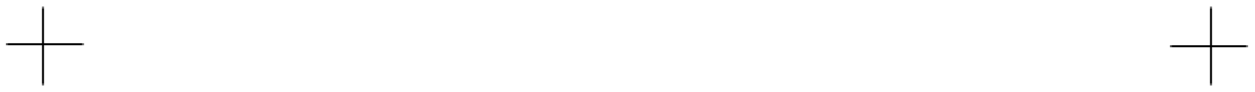

Page Break

Q48

Now please click right in the centre of this left cross and right in the centre of this right cross. Try to be as accurate as possible, and again if the mouse cannot fall exactly in the centre please ensure you do the same for each click. You can try as many times as you like.

Page Break

Q49
And finally, please do the same for the two crosses on this page.

Page Break

Q47 Browser Meta Info
Browser (1)
Version (2)
Operating System (3)
Screen Resolution (4)
Flash Version (5)
Java Support (6)
User Agent (7)

End of Block: Start

Start of Block: Block1

Q1

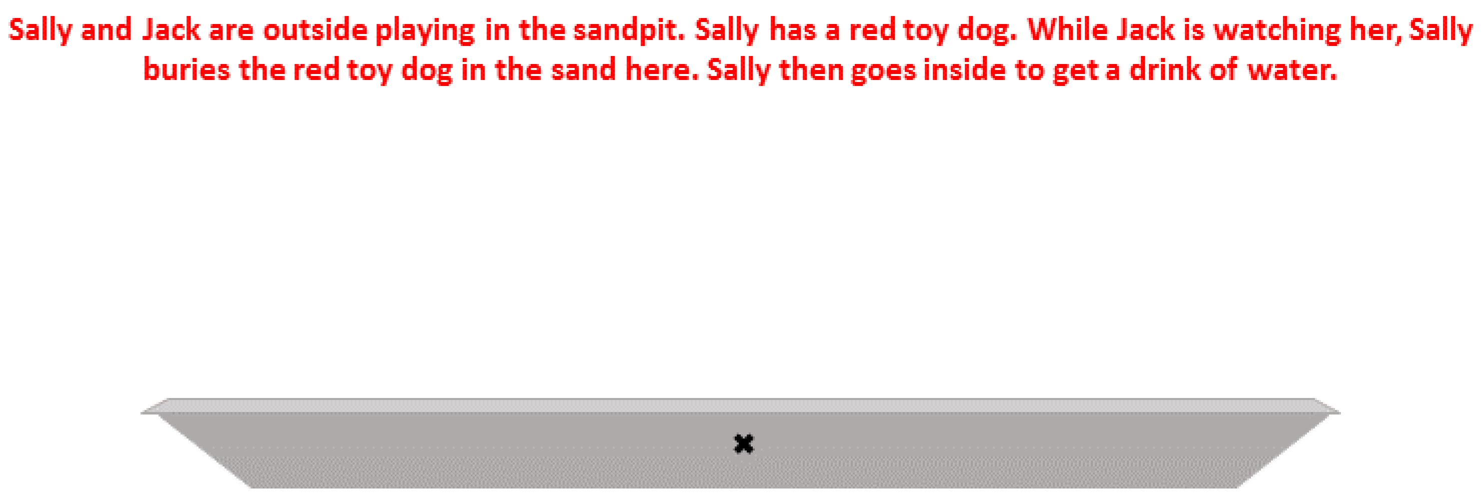

Page Break

Q2

While Sally is inside the house, Jack digs the red toy dog out and hides it here. He smooths over the sand so it looks undisturbed.

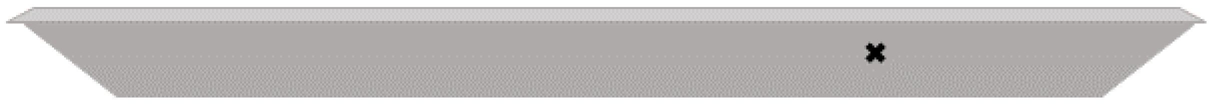

Page Break

Q3
Now try to find as many words as you can in the puzzle below and type them in the field at the bottom of the page. You have 20 seconds!

____

____

____

____

____

Q5 Timing
First Click (1)
Last Click (2)
Page Submit (3)
Click Count (4)

Page Break

Q4
Please click on the image to answer.

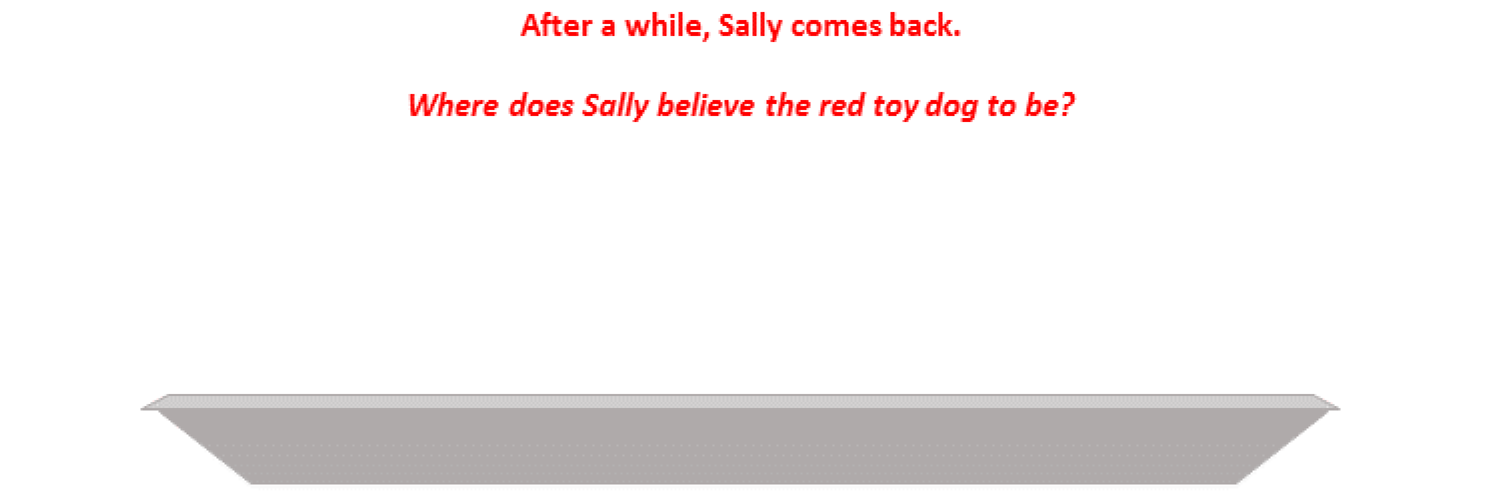

End of Block: Block1

Start of Block: Block2

Q12

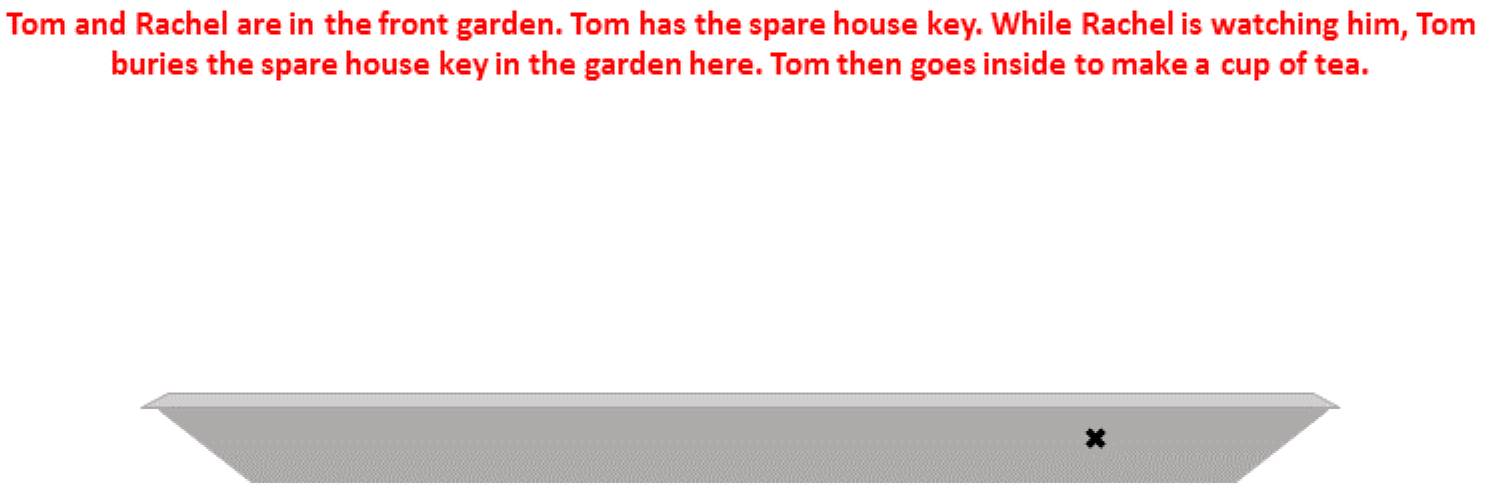

Page Break

Q13

While Tom is inside the house, Rachel digs the spare house key out and hides it here. She smooths over the earth so it looks undisturbed.

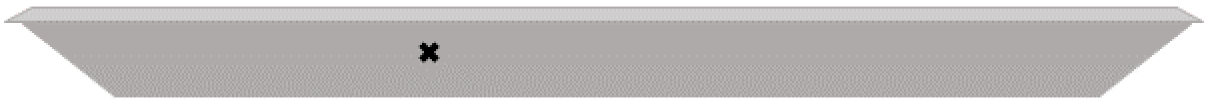

Page Break

Q14
Now try to find as many words as you can in the puzzle below and type them in the field at the bottom of the page. You have 20 seconds!

____

____

____

____

____

Q15 Timing
First Click (1)
Last Click (2)
Page Submit (3)
Click Count (4)

Page Break

Q16
Please click on the image to answer.

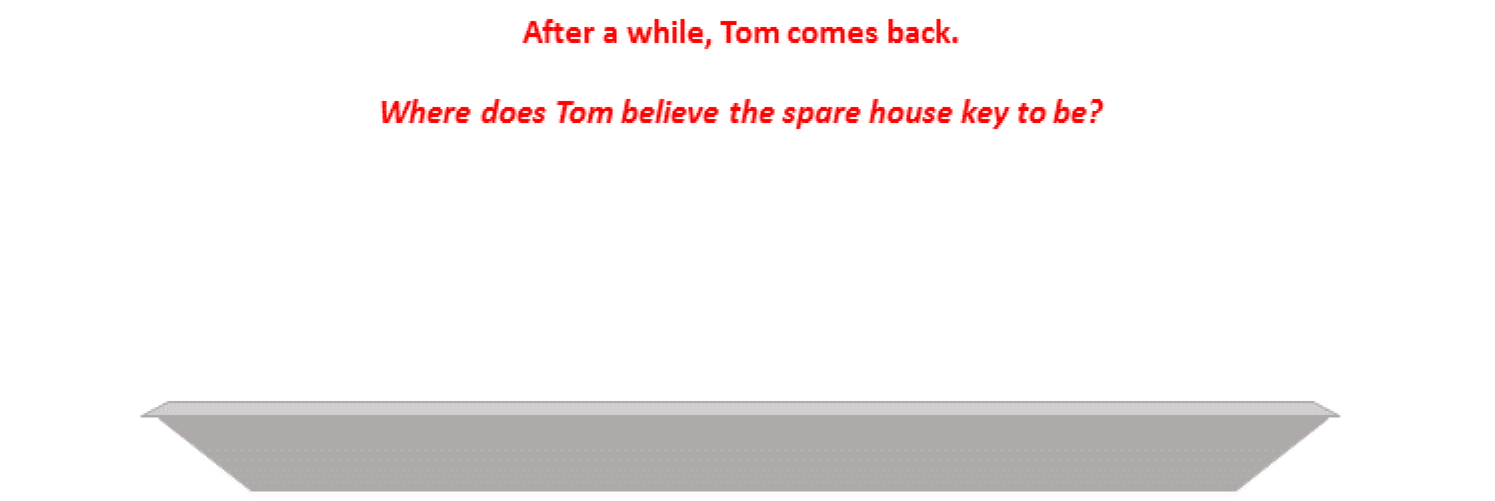

End of Block: Block2

Start of Block: Block3

Q17

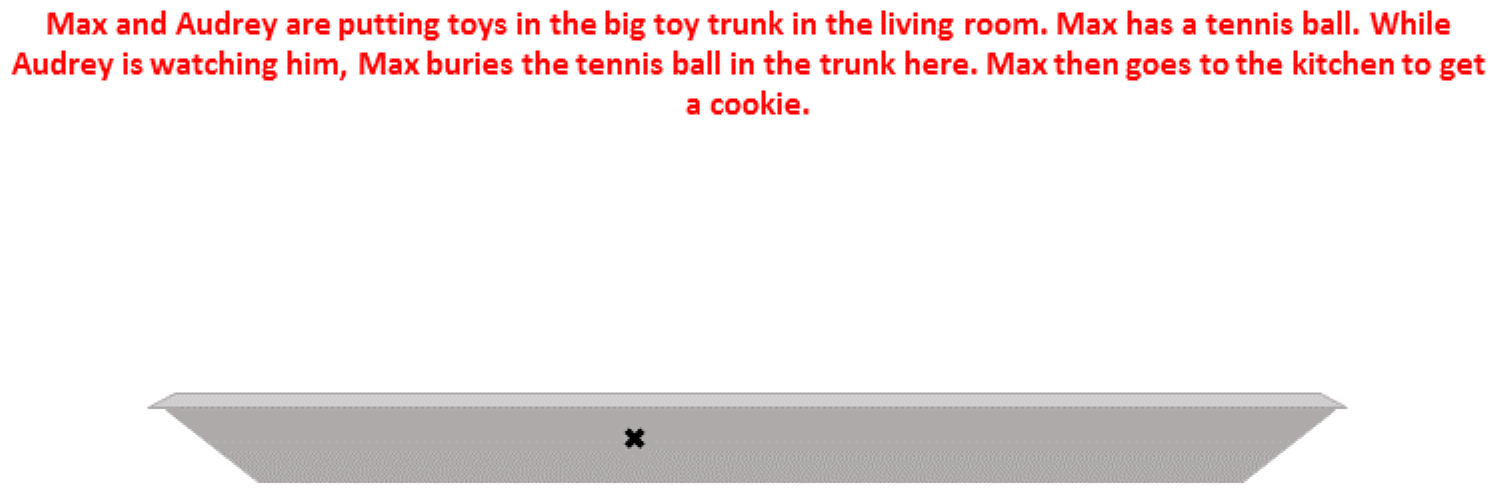

Page Break

Q18

While Max is in the kitchen, Audrey digs the tennis ball out and hides it here. She puts everything else in the trunk back where it was so it looks undisturbed.

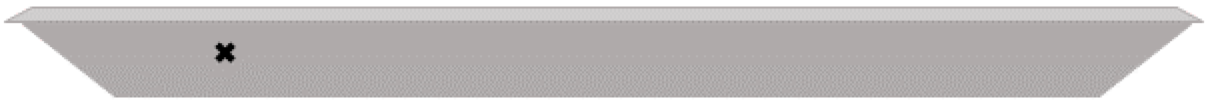

Page Break

Q19
Now try to find as many words as you can in the puzzle below and type them in the field at the bottom of the page. You have 20 seconds!

____

____

____

____

____

Q20 Timing
First Click (1)
Last Click (2)
Page Submit (3)
Click Count (4)

Page Break

Q21
Please click on the image to answer.

After a while, Max comes back.

Where does Max believe the tennis ball to be?

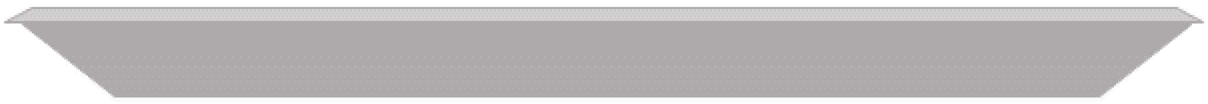

End of Block: Block3

Start of Block: Block4

Q26

Rebecca and Steve are in the restaurant kitchen. Rebecca has the tips jar. While Steve is watching her, Rebecca buries the tips jar in the freezer here. Rebecca then goes outside to smoke a cigarette.

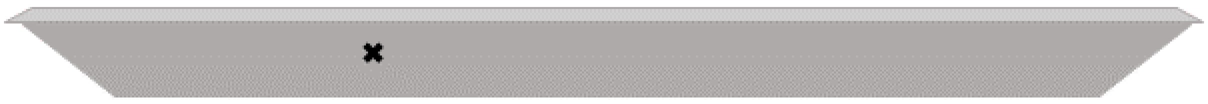

Page Break

Q27

While Rebecca is outside, Steve digs the tips jar out and hides it here. He puts everything else in the freezer back where it was so it looks undisturbed.

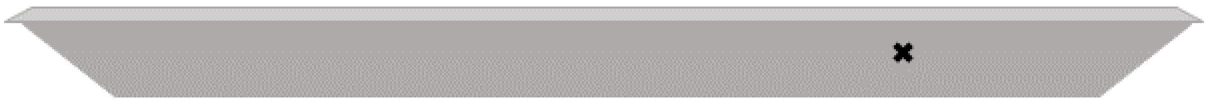

Page Break

Q28
Now try to find as many words as you can in the puzzle below and type them in the field at the bottom of the page. You have 20 seconds!

____

____

____

____

____

Q29 Timing
First Click (1)
Last Click (2)
Page Submit (3)
Click Count (4)

Page Break

Q30
Please click on the image to answer.

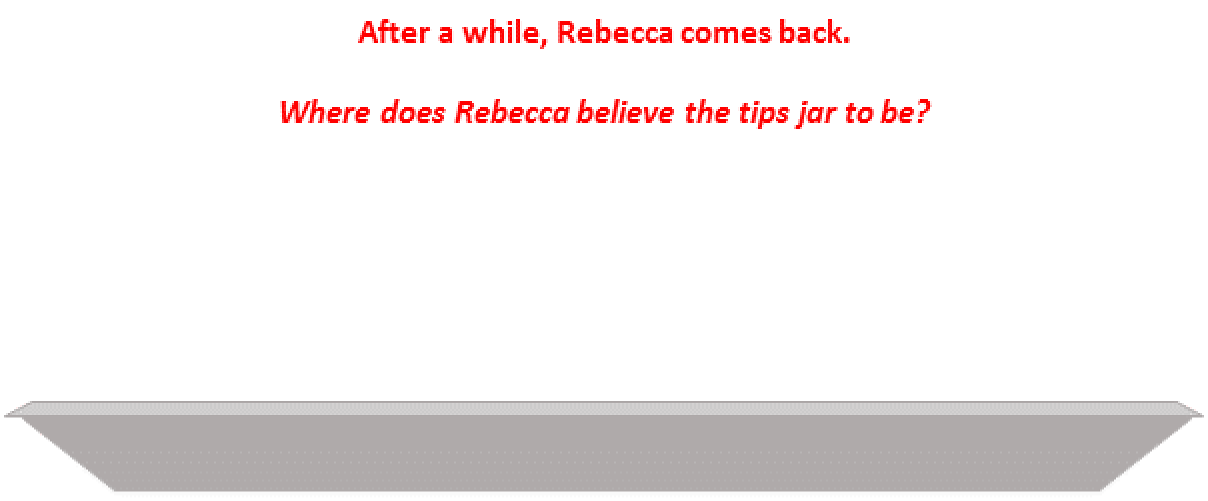

End of Block: Block4

Start of Block: Block5

Q31

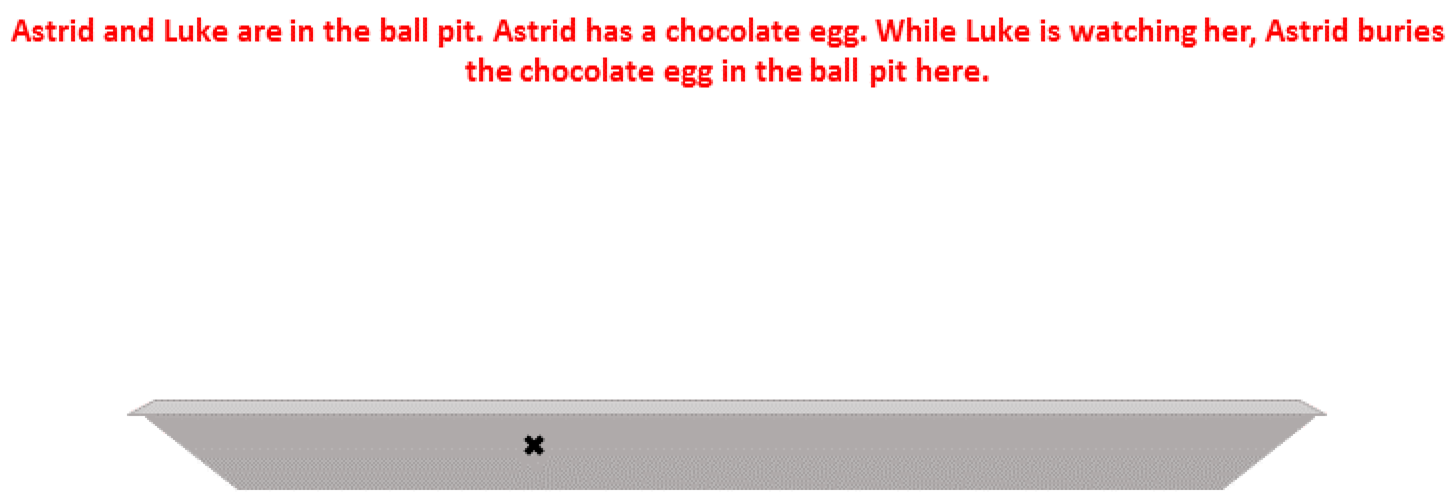

Page Break

Q32

While Luke is still watching her, Astrid digs the chocolate egg out and hides it here. She smooths over the balls so they look undisturbed. Astrid then goes inside to get a drink.

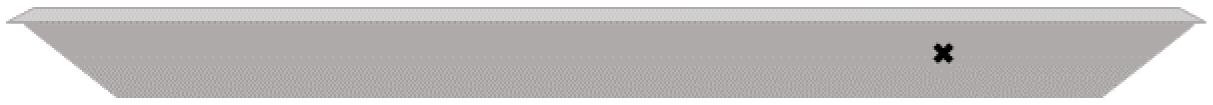

Page Break

Q33
Now try to find as many words as you can in the puzzle below and type them in the field at the bottom of the page. You have 20 seconds!

____

____

____

____

____

Q34 Timing
First Click (1)
Last Click (2)
Page Submit (3)
Click Count (4)

Page Break

Q35
Please click on the image to answer.

After a while, Astrid comes back.

Where is the chocolate egg?

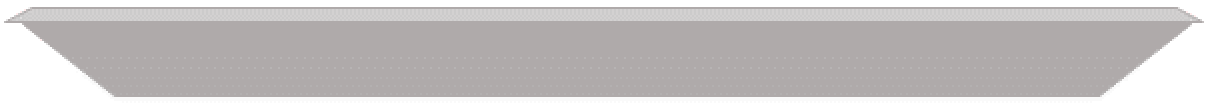

End of Block: Block5

Start of Block: Block6

Q36

Peter and Lisa are by the hotel garden pond. Peter has a bag of jewels. While Lisa is watching him, Peter buries the bag of jewels in the pond here. Peter then goes inside to make a call.

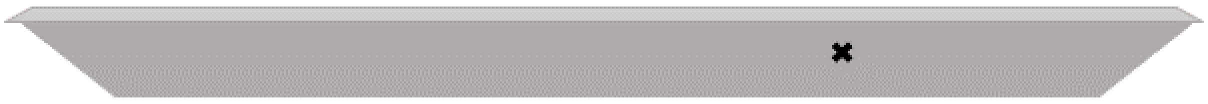

Page Break

Q37

While Peter is inside, Lisa digs the bag of jewels out and hides it here. She watches the surface of the pond until it looks undisturbed.

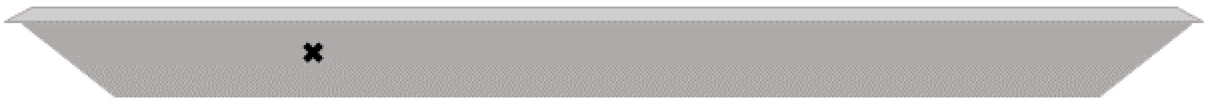

Page Break

Q38
Now try to find as many words as you can in the puzzle below and type them in the field at the bottom of the page. You have 20 seconds!

____

____

____

____

____

Q39 Timing
First Click (1)
Last Click (2)
Page Submit (3)
Click Count (4)

Page Break

Q40
Please click on the image to answer.

After a while, Peter comes back.

Where do you remember he buried the bag of jewels?

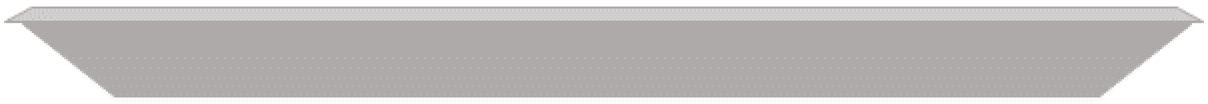

End of Block: Block6

Start of Block: Block7

Q41

Chloe and James are hiking in the snow. Chloe has a bottle of beer. While James is watching her, Chloe buries the bottle of beer in the snow here. Chloe then goes into their tent to check her emails.

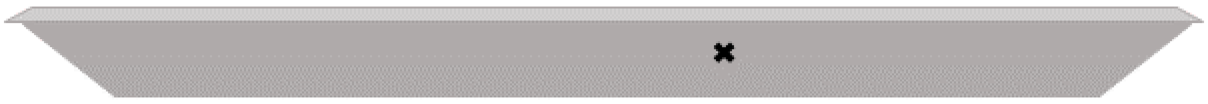

Page Break

Q42

While Chloe is away, James digs the bottle of beer out and hides it here. He smooths over the snow so it looks undisturbed.

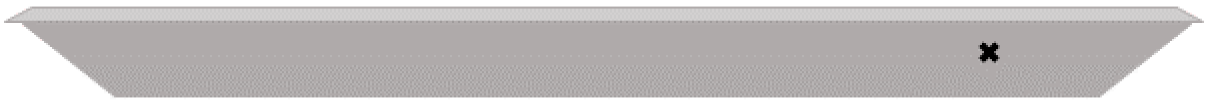

Page Break

Q43
Now try to find as many words as you can in the puzzle below and type them in the field at the bottom of the page. You have 20 seconds!

____

____

____

____

____

Q44 Timing
First Click (1)
Last Click (2)
Page Submit (3)
Click Count (4)

Page Break

Q45
Please click on the image to answer.

After a while, Chloe comes back.

Where do you remember she buried the bottle of beer?

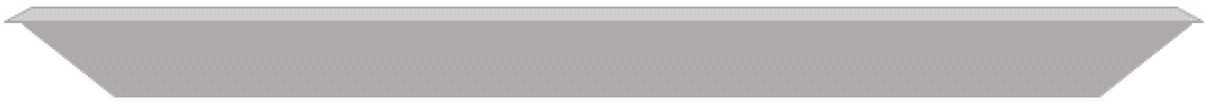

End of Block: Block7

Start of Block: Block8

Q46

Sarah and Alan are on the beach. Sarah has their passports. While Alan is watching her, Sarah buries the passports in the sand here. Sarah then goes to a shop to get some ice cream.

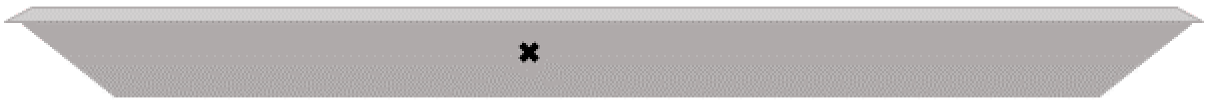

Page Break

Q47

While Sarah is away, Alan digs the passports out and hides them here. He smooths over the sand so it looks undisturbed.

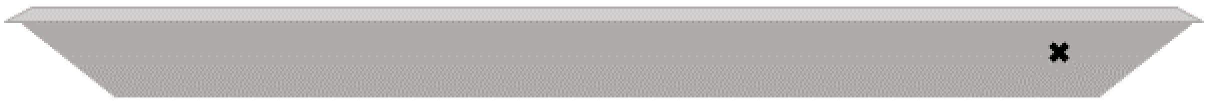

Page Break

Q48
Now try to find as many words as you can in the puzzle below and type them in the field at the bottom of the page. You have 20 seconds!

____

____

____

____

____

Q49 Timing
First Click (1)
Last Click (2)
Page Submit (3)
Click Count (4)

Page Break

Q50
Please click on the image to answer.

After a while, Sarah comes back.

Where do you remember she buried the passports?

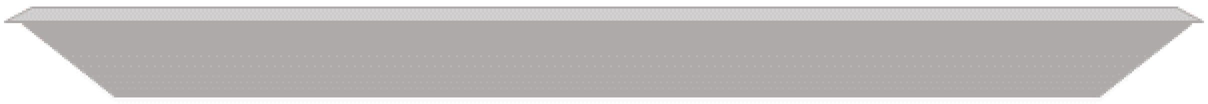

End of Block: Block8

Start of Block: Block9

Q51

John and Alice are in the garden with a planter. John has a flower bulb. While Alice is watching him, John buries the flower bulb in the planter here. John then goes inside to get some water.

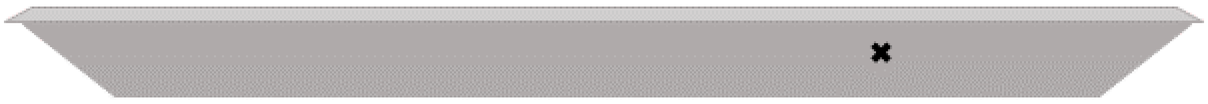

Page Break

Q52

While John is away, Alice digs the flower bulb out and hides it here. She smooths over the soil so it looks undisturbed.

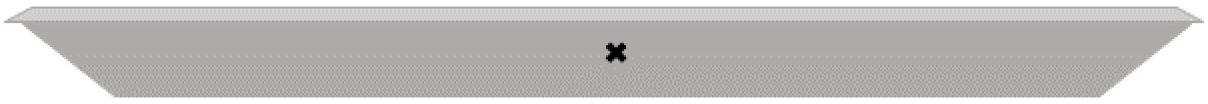

Page Break

Q53
Now try to find as many words as you can in the puzzle below and type them in the field at the bottom of the page. You have 20 seconds!

____

____

____

____

____

Q54 Timing
First Click (1)
Last Click (2)
Page Submit (3)
Click Count (4)

Page Break

Q55
Please click on the image to answer.

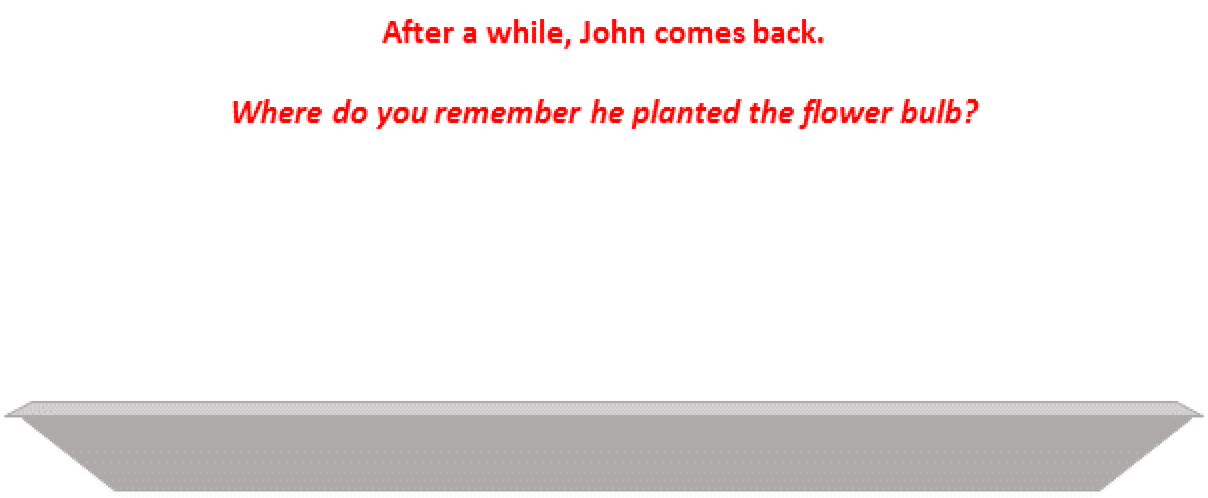

End of Block: Block9

Start of Block: Debrief

Q62 **Participant Debriefing Sheet:**

Thank you for taking part in our study.
What was the study about?
The experiment you took part in is part of a larger study designed to test how people use their own knowledge when having to take into account what others remember or believe. We know that adults tend to expect that other adults know what they themselves know. The critical component of this study was to see whether you were influenced by where you knew an object to be when you had to imagine where someone else remembered or believed it to be.

Page Break

Q62 ***Further Information*** *You are welcome to ask the researchers about the study.*

If you would like to know more about the study you can contact Dr. Steven Samuel (ss2391@cam.ac.uk) for further information.

Page Break

End of Block: Debrief

NEW sandbox: ownmem-otheraction

Start of Block: Prolific Info

Q65 Before you start, please:

– maximize your browser window;
– switch off phone/e-mail/music & anything else distracting
- and please enter your Prolific ID [it can be found at the top of this webpage or when going to your account info]:\\\

[To continue to the next question at any point in the survey please use the arrow button at the bottom right of the page]

____

End of Block: Prolific Info

Start of Block: Participant Info

Q54 Before you decide to take part in this study it is important for you to understand why the research is being done and what it will involve. Please take time to read the following information carefully and discuss it with others if you wish. A member of the team can be contacted if there is anything that is not clear or if you would like more information. Take time to decide whether or not you wish to take part.

Page Break

Q55 This study investigates how adults reason about what others want, believe and act. The study fits in with a larger research theme of how children and adults reason about other individuals' mental states and how both children and adults attribute beliefs and desires to other people.

Page Break

Q56 During the study you will be asked to make judgments about what others can see, hear or do. You may also be asked to predict how another person would act based on what they have seen or heard. The experiment will take approximately 10 minutes to complete.

Page Break

Q57 Participation in the study is entirely voluntary and you can withdraw at any point. The results of the study may be written up or presented at conferences. Your participation will remain confidential and if individual data is presented there will be no means of identifying the individuals involved.

Page Break

Q58 If you would like to know more about the study or have any questions you can contact Steven Samuel (ss2391@cam.ac.uk) for further information.

The project has received ethical approval from the Psychology Ethics Committee of the University of Cambridge.

Page Break

End of Block: Participant Info

Start of Block: Consent Page 1/6

Q81 I confirm that I have read and understand the participant information.

○ Yes (1)

○ No (2)

Q93 I have had the opportunity to ask questions and had them answered.

○ Yes (1)

○ No (2)

Q105 I understand that all personal information will remain confidential and that all efforts will be made to ensure that I cannot be identified (except as might be required by law).

○ Yes (1)

○ No (2)

Q117 I agree that data gathered in this study may be stored anonymously and securely, and may be used for future research.

○ Yes (1)

○ No (2)

Q129 I understand that my participation is voluntary and that I am free to withdraw at any time without giving a reason.

○ Yes (1)

○ No (2)

Q141 I agree to take part in this study.

○ Yes (1)

○ No (2)

End of Block: Consent Page 1/6

Start of Block: Consent Page 2/6

Q66 There are two parts to the study.

First you will be asked to click on various locations on your screen (this helps us with calibration).

Second (the main part of the study) you will read some short stories about objects being hidden. Please pay careful attention to the story and the accompanying visual information. After each story you will have 20 seconds to find words in a wordsearch. After each wordsearch you will be asked a question about the story that you have just read. Total experiment time is approximately 10 minutes.

The calibration process is next...

End of Block: Consent Page 2/6

Start of Block: Consent Page 3/6

Start of Block: Consent Page 4/6

Start of Block: Consent Page 5/6

Start of Block: Consent Page 6/6

Start of Block: Start

Q17
Please click right in the centre of the left cross (first) and right in the centre of the right cross (second). Try to be as accurate as possible. If the mouse-controlled cross does not align perfectly with the crosses on the screen (for example it always has to be a fraction to the left or right), please do the same for each cross. Make sure a marker is left on each cross before moving on to the next page. You can try as many times as you like.

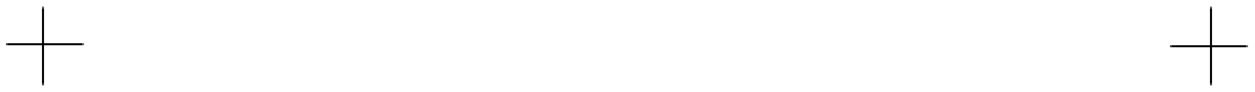

Page Break

Q48

Now please click right in the centre of this left cross and right in the centre of this right cross. Try to be as accurate as possible, and again if the mouse cannot fall exactly in the centre please ensure you do the same for each click. You can try as many times as you like.

Page Break

Q49
And finally, please do the same for the two crosses on this page.

Page Break

Q47 Browser Meta Info
Browser (1)
Version (2)
Operating System (3)
Screen Resolution (4)
Flash Version (5)
Java Support (6)
User Agent (7)

End of Block: Start

Start of Block: Block1

Q1

Sally and Jack are outside playing in the sandpit. Sally has a red toy dog. While Jack is watching her, Sally buries the red toy dog in the sand here. Sally then goes inside to get a drink of water.

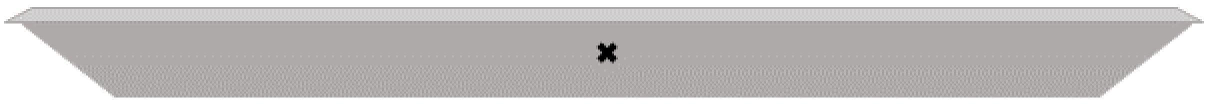

Page Break

Q2

While Sally is inside the house, Jack digs the red toy dog out and hides it here. He smooths over the sand so it looks undisturbed.

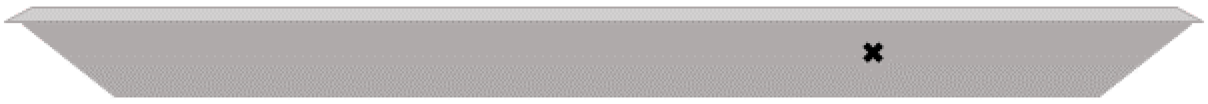

Page Break

Q3
Now try to find as many words as you can in the puzzle below and type them in the field at the bottom of the page. You have 20 seconds!

____

____

____

____

____

Q5 Timing
First Click (1)
Last Click (2)
Page Submit (3)
Click Count (4)

Page Break

Q4
Please click on the image to answer.

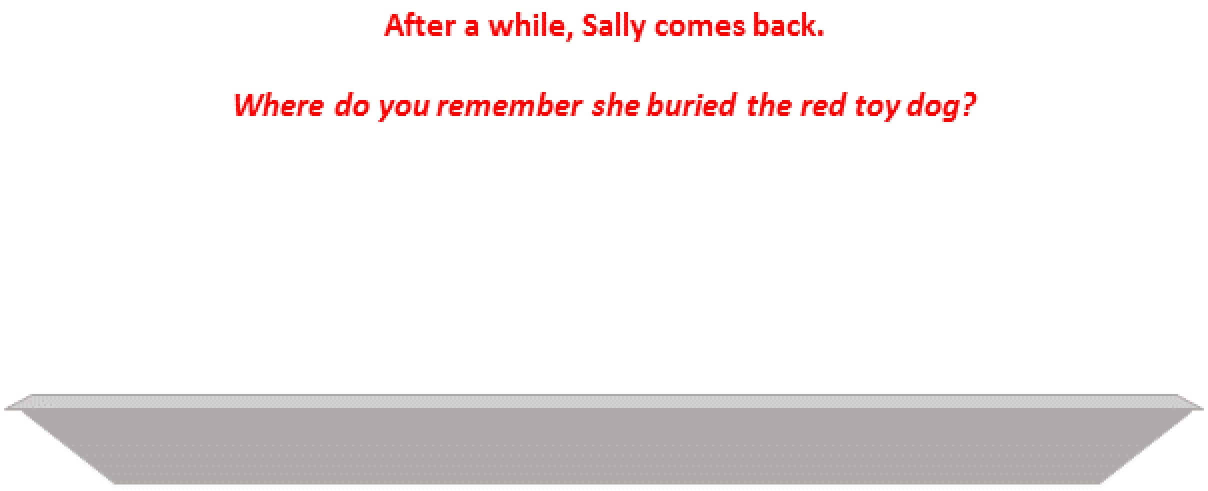

End of Block: Block1

Start of Block: Block2

Q12

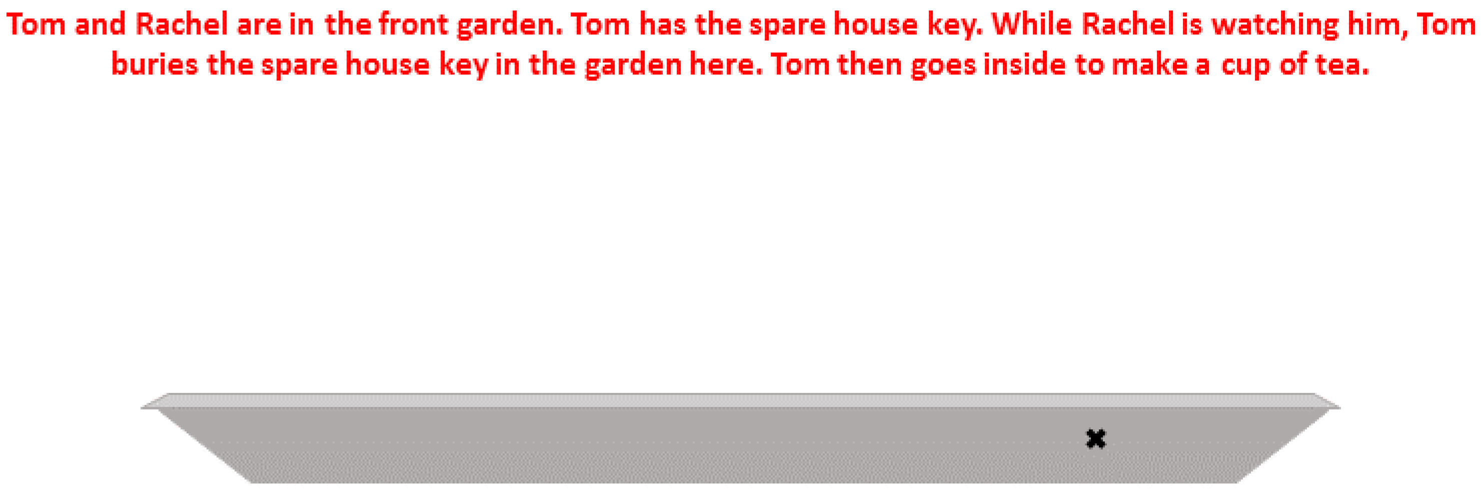

Page Break

Q13

While Tom is inside the house, Rachel digs the spare house key out and hides it here. She smooths over the earth so it looks undisturbed.

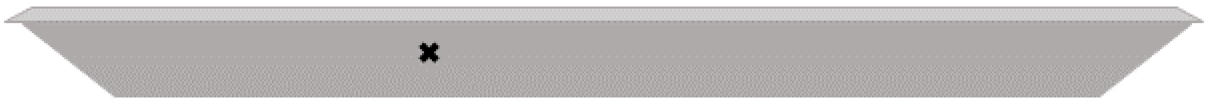

Page Break

Q14
Now try to find as many words as you can in the puzzle below and type them in the field at the bottom of the page. You have 20 seconds!

____

____

____

____

____

Q15 Timing
First Click (1)
Last Click (2)
Page Submit (3)
Click Count (4)

Page Break

Q16
Please click on the image to answer.

After a while, Tom comes back.

Where do you remember he buried the spare house key?

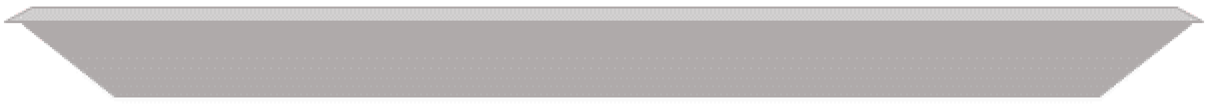

End of Block: Block2

Start of Block: Block3

Q17

Max and Audrey are putting toys in the big toy trunk in the living room. Max has a tennis ball. While Audrey is watching him, Max buries the tennis ball in the trunk here. Max then goes to the kitchen to get a cookie.

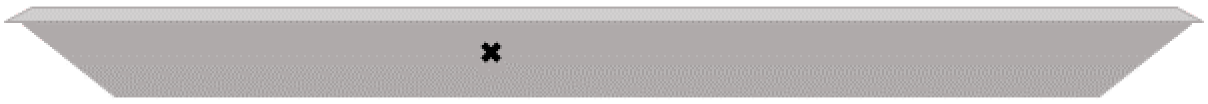

Page Break

Q18

While Max is in the kitchen, Audrey digs the tennis ball out and hides it here. She puts everything else in the trunk back where it was so it looks undisturbed.

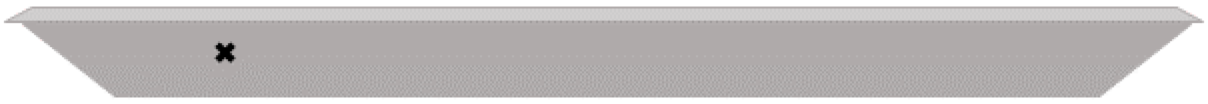

Page Break

Q19
Now try to find as many words as you can in the puzzle below and type them in the field at the bottom of the page. You have 20 seconds!

____

____

____

____

____

Q20 Timing
First Click (1)
Last Click (2)
Page Submit (3)
Click Count (4)

Page Break

Q21
Please click on the image to answer.

After a while, Max comes back.

Where do you remember he buried the tennis ball?

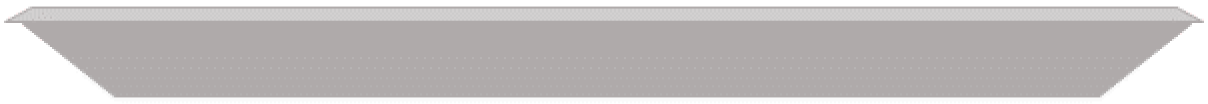

End of Block: Block3

Start of Block: Block4

Q26

Rebecca and Steve are in the restaurant kitchen. Rebecca has the tips jar. While Steve is watching her, Rebecca buries the tips jar in the freezer here. Rebecca then goes outside to smoke a cigarette.

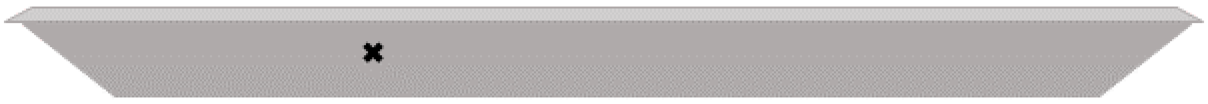

Page Break

Q27

While Rebecca is outside, Steve digs the tips jar out and hides it here. He puts everything else in the freezer back where it was so it looks undisturbed.

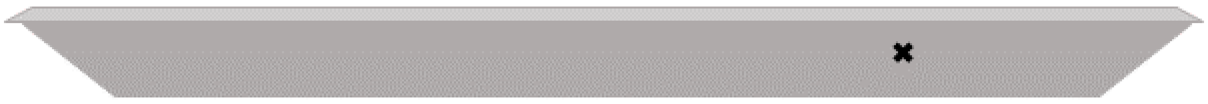

Page Break

Q28
Now try to find as many words as you can in the puzzle below and type them in the field at the bottom of the page. You have 20 seconds!

____

____

____

____

____

Q29 Timing
First Click (1)
Last Click (2)
Page Submit (3)
Click Count (4)

Page Break

Q30
Please click on the image to answer.

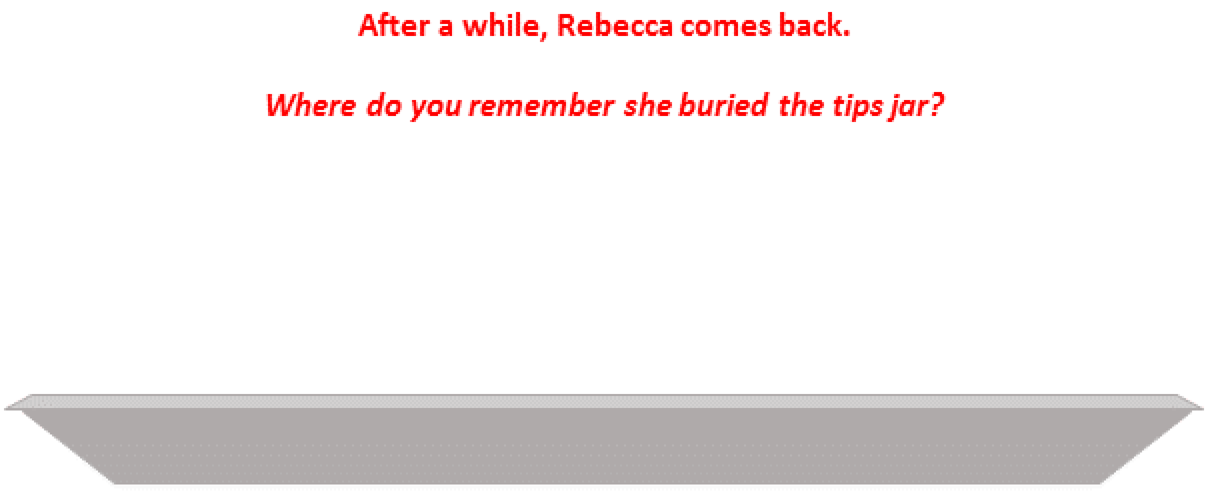

End of Block: Block4

Start of Block: Block5

Q31

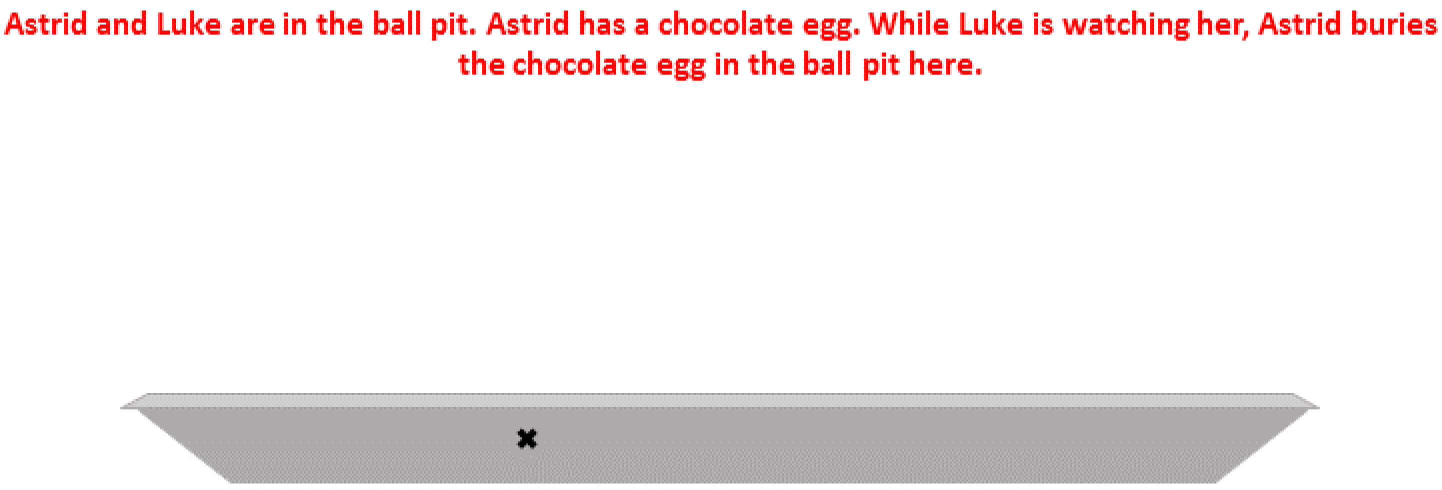

Page Break

Q32

While Luke is still watching her, Astrid digs the chocolate egg out and hides it here. She smooths over the balls so they look undisturbed. Astrid then goes inside to get a drink.

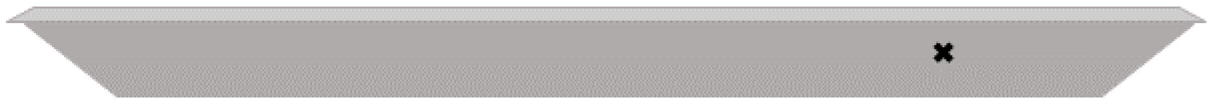

Page Break

Q33
Now try to find as many words as you can in the puzzle below and type them in the field at the bottom of the page. You have 20 seconds!

____

____

____

____

____

Q34 Timing
First Click (1)
Last Click (2)
Page Submit (3)
Click Count (4)

Page Break

Q35
Please click on the image to answer.

After a while, Astrid comes back.

Where is the chocolate egg?

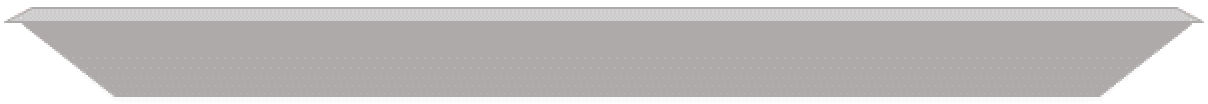

End of Block: Block5

Start of Block: Block6

Q36

Peter and Lisa are by the hotel garden pond. Peter has a bag of jewels. While Lisa is watching him, Peter buries the bag of jewels in the pond here. Peter then goes inside to make a call.

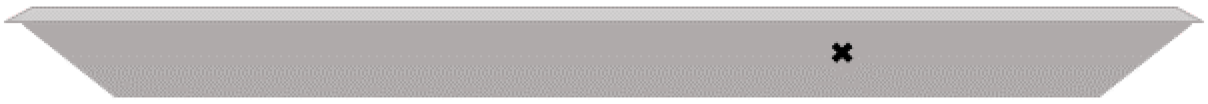

Page Break

Q37

While Peter is inside, Lisa digs the bag of jewels out and hides it here. She watches the surface of the pond until it looks undisturbed.

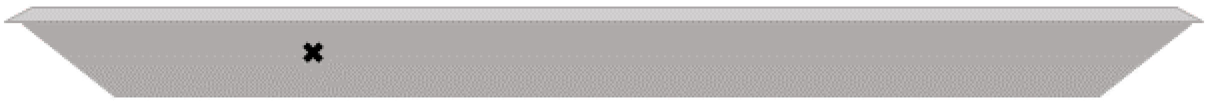

Page Break

Q38
Now try to find as many words as you can in the puzzle below and type them in the field at the bottom of the page. You have 20 seconds!

____

____

____

____

____

Q39 Timing
First Click (1)
Last Click (2)
Page Submit (3)
Click Count (4)

Page Break

Q40
Please click on the image to answer.

After a while, Peter comes back.

Where will Peter look for the bag of jewels?

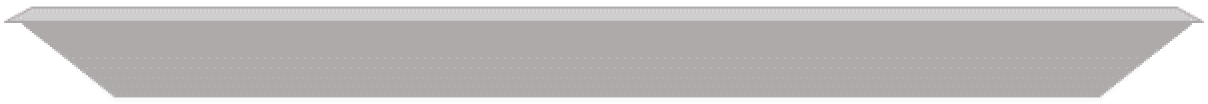

End of Block: Block6

Start of Block: Block7

Q41

Chloe and James are hiking in the snow. Chloe has a bottle of beer. While James is watching her, Chloe buries the bottle of beer in the snow here. Chloe then goes into their tent to check her emails.

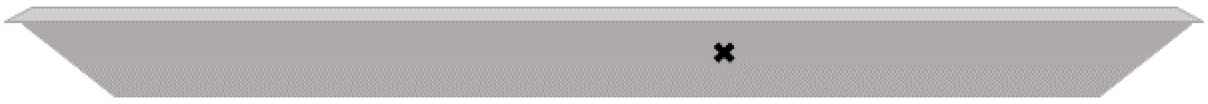

Page Break

Q42

While Chloe is away, James digs the bottle of beer out and hides it here. He smooths over the snow so it looks undisturbed.

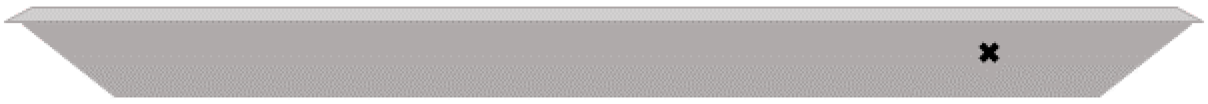

Page Break

Q43
Now try to find as many words as you can in the puzzle below and type them in the field at the bottom of the page. You have 20 seconds!

____

____

____

____

____

Q44 Timing
First Click (1)
Last Click (2)
Page Submit (3)
Click Count (4)

Page Break

Q45
Please click on the image to answer.

After a while, Chloe comes back.

Where will Chloe look for the bottle of beer?

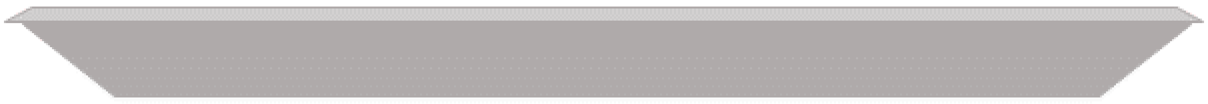

End of Block: Block7

Start of Block: Block8

Q46

Sarah and Alan are on the beach. Sarah has their passports. While Alan is watching her, Sarah buries the passports in the sand here. Sarah then goes to a shop to get some ice cream.

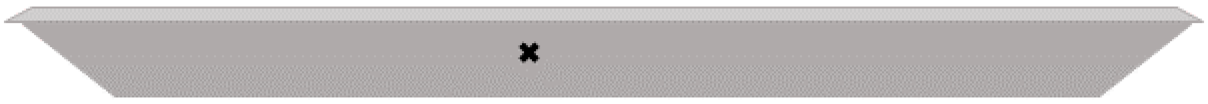

Page Break

Q47

While Sarah is away, Alan digs the passports out and hides them here. He smooths over the sand so it looks undisturbed.

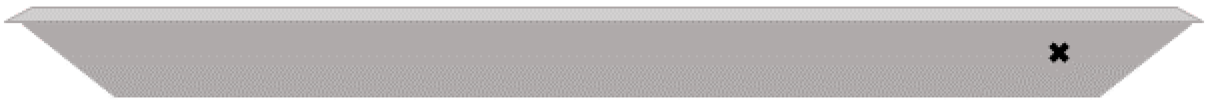

Page Break

Q48
Now try to find as many words as you can in the puzzle below and type them in the field at the bottom of the page. You have 20 seconds!

____

____

____

____

____

Q49 Timing
First Click (1)
Last Click (2)
Page Submit (3)
Click Count (4)

Page Break

Q50
Please click on the image to answer.

After a while, Sarah comes back.

Where will Sarah look for the passports?

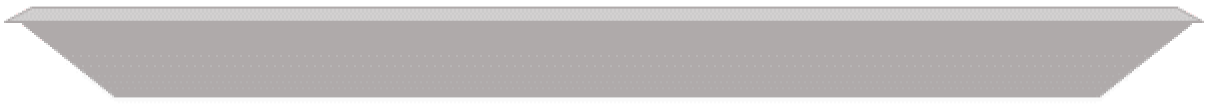

End of Block: Block8

Start of Block: Block9

Q51

John and Alice are in the garden with a planter. John has a flower bulb. While Alice is watching him, John buries the flower bulb in the planter here. John then goes inside to get some water.

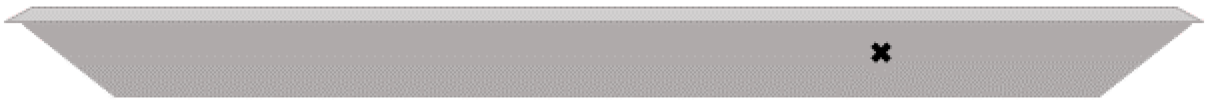

Page Break

Q52

While John is away, Alice digs the flower bulb out and hides it here. She smooths over the soil so it looks undisturbed.

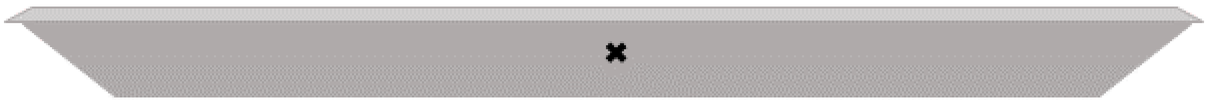

Page Break

Q53
Now try to find as many words as you can in the puzzle below and type them in the field at the bottom of the page. You have 20 seconds!

____

____

____

____

____

Q54 Timing
First Click (1)
Last Click (2)
Page Submit (3)
Click Count (4)

Page Break

Q55
Please click on the image to answer.

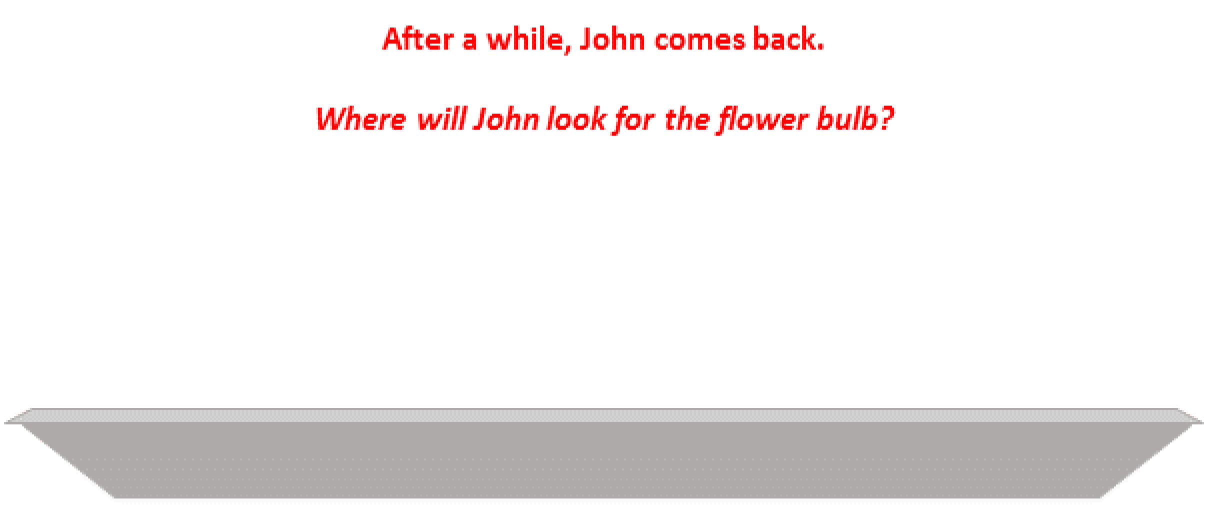

End of Block: Block9

Start of Block: Debrief

Q62 **Participant Debriefing Sheet:**

Thank you for taking part in our study.
What was the study about?
The experiment you took part in is part of a larger study designed to test how people use their own knowledge when having to take into account what others remember or believe. We know that adults tend to expect that other adults know what they themselves know. The critical component of this study was to see whether you were influenced by where you knew an object to be when you had to imagine where someone else remembered or believed it to be.

Page Break

Q62 ***Further Information*** *You are welcome to ask the researchers about the study.*

If you would like to know more about the study you can contact Dr. Steven Samuel (ss2391@cam.ac.uk) for further information.

Page Break

End of Block: Debrief

NEW sandbox: otheraction-ownmem

Start of Block: Prolific Info

Q65 Before you start, please:

– maximize your browser window;
– switch off phone/e-mail/music & anything else distracting
- and please enter your Prolific ID [it can be found at the top of this webpage or when going to your account info]:\\\

[To continue to the next question at any point in the survey please use the arrow button at the bottom right of the page]

____

End of Block: Prolific Info

Start of Block: Participant Info

Q54 Before you decide to take part in this study it is important for you to understand why the research is being done and what it will involve. Please take time to read the following information carefully and discuss it with others if you wish. A member of the team can be contacted if there is anything that is not clear or if you would like more information. Take time to decide whether or not you wish to take part.

Page Break

Q55 This study investigates how adults reason about what others want, believe and act. The study fits in with a larger research theme of how children and adults reason about other individuals' mental states and how both children and adults attribute beliefs and desires to other people.

Page Break

Q56 During the study you will be asked to make judgments about what others can see, hear or do. You may also be asked to predict how another person would act based on what they have seen or heard. The experiment will take approximately 10 minutes to complete.

Page Break

Q57 Participation in the study is entirely voluntary and you can withdraw at any point. The results of the study may be written up or presented at conferences. Your participation will remain confidential and if individual data is presented there will be no means of identifying the individuals involved.

Page Break

Q58 If you would like to know more about the study or have any questions you can contact Steven Samuel (ss2391@cam.ac.uk) for further information.

The project has received ethical approval from the Psychology Ethics Committee of the University of Cambridge.

Page Break

End of Block: Participant Info

Start of Block: Consent Page 1/6

Q81 I confirm that I have read and understand the participant information.

- Yes (1)
- No (2)

Q93 I have had the opportunity to ask questions and had them answered.

- Yes (1)
- No (2)

Q105 I understand that all personal information will remain confidential and that all efforts will be made to ensure that I cannot be identified (except as might be required by law).

- Yes (1)
- No (2)

Q117 I agree that data gathered in this study may be stored anonymously and securely, and may be used for future research.

- Yes (1)
- No (2)

Q129 I understand that my participation is voluntary and that I am free to withdraw at any time without giving a reason.

- Yes (1)
- No (2)

Q141 I agree to take part in this study.

- Yes (1)
- No (2)

End of Block: Consent Page 1/6

Start of Block: Consent Page 2/6

Q66 There are two parts to the study.

First you will be asked to click on various locations on your screen (this helps us with calibration).

Second (the main part of the study) you will read some short stories about objects being hidden. Please pay careful attention to the story and the accompanying visual information. After each story you will have 20 seconds to find words in a wordsearch. After each wordsearch you will be asked a question about the story that you have just read. Total experiment time is approximately 10 minutes.

The calibration process is next...

End of Block: Consent Page 2/6

Start of Block: Consent Page 3/6

Start of Block: Consent Page 4/6

Start of Block: Consent Page 5/6

Start of Block: Consent Page 6/6

Start of Block: Start

Q17
Please click right in the centre of the left cross (first) and right in the centre of the right cross (second). Try to be as accurate as possible. If the mouse-controlled cross does not align perfectly with the crosses on the screen (for example it always has to be a fraction to the left or right), please do the same for each cross. Make sure a marker is left on each cross before moving on to the next page. You can try as many times as you like.

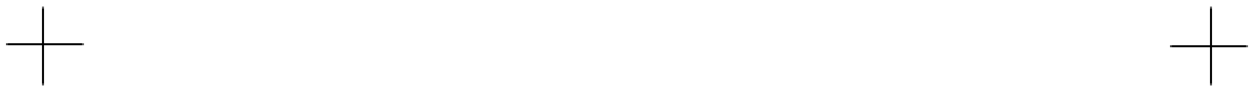

Page Break

Q48

Now please click right in the centre of this left cross and right in the centre of this right cross. Try to be as accurate as possible, and again if the mouse cannot fall exactly in the centre please ensure you do the same for each click. You can try as many times as you like.

Page Break

Q49
And finally, please do the same for the two crosses on this page.

Page Break

Q47 Browser Meta Info
Browser (1)
Version (2)
Operating System (3)
Screen Resolution (4)
Flash Version (5)
Java Support (6)
User Agent (7)

End of Block: Start

Start of Block: Block1

Q1

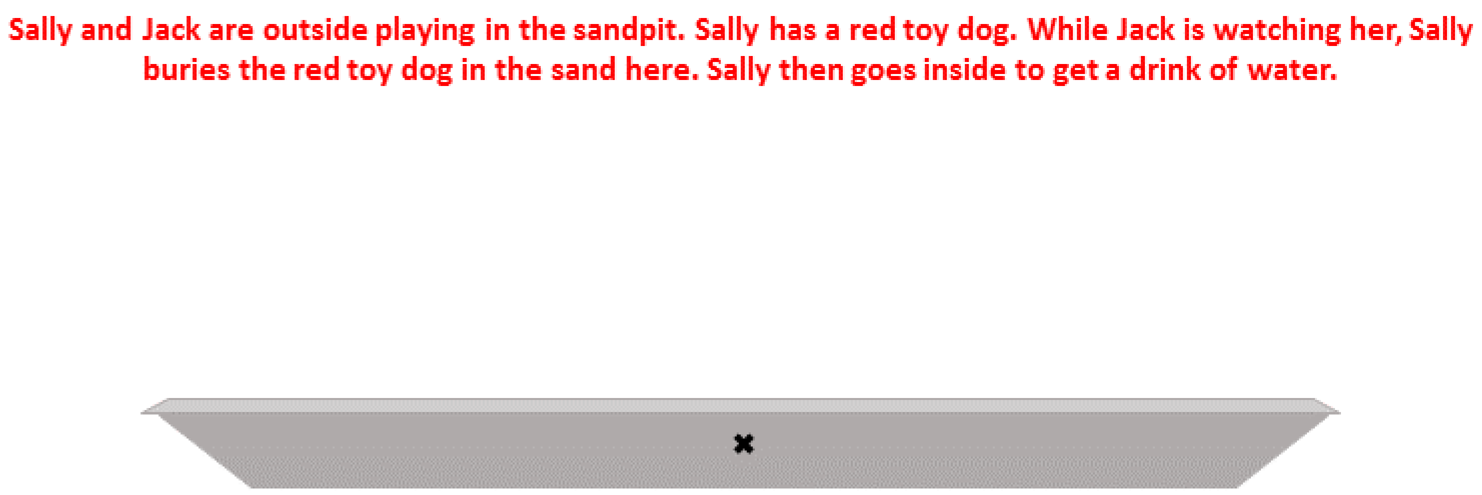

Page Break

Q2

While Sally is inside the house, Jack digs the red toy dog out and hides it here. He smooths over the sand so it looks undisturbed.

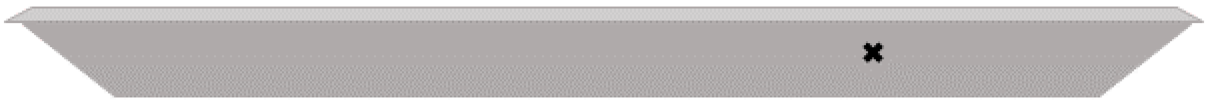

Page Break

Q3
Now try to find as many words as you can in the puzzle below and type them in the field at the bottom of the page. You have 20 seconds!

____

____

____

____

____

Q5 Timing
First Click (1)
Last Click (2)
Page Submit (3)
Click Count (4)

Page Break

Q4
Please click on the image to answer.

After a while, Sally comes back.

Where will Sally look for the red toy dog?

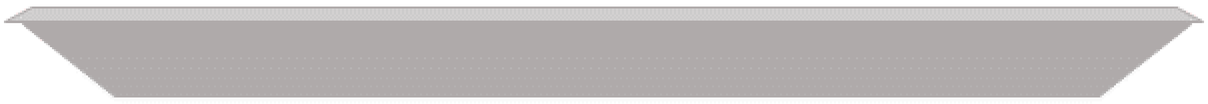

End of Block: Block1

Start of Block: Block2

Q12

Tom and Rachel are in the front garden. Tom has the spare house key. While Rachel is watching him, Tom buries the spare house key in the garden here. Tom then goes inside to make a cup of tea.

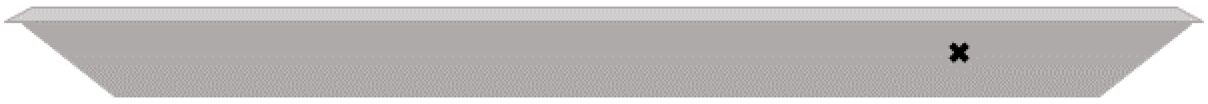

Page Break

Q13

While Tom is inside the house, Rachel digs the spare house key out and hides it here. She smooths over the earth so it looks undisturbed.

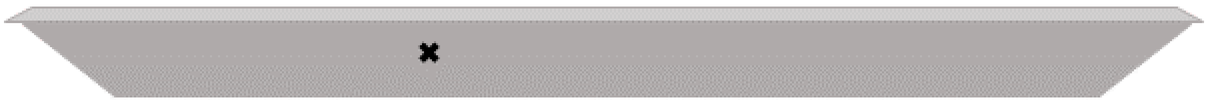

Page Break

Q14
Now try to find as many words as you can in the puzzle below and type them in the field at the bottom of the page. You have 20 seconds!

____

____

____

____

____

Q15 Timing
First Click (1)
Last Click (2)
Page Submit (3)
Click Count (4)

Page Break

Q16
Please click on the image to answer.

After a while, Tom comes back.

Where will Tom look for the spare house key?

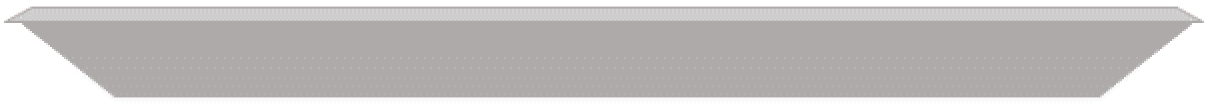

End of Block: Block2

Start of Block: Block3

Q17

Max and Audrey are putting toys in the big toy trunk in the living room. Max has a tennis ball. While Audrey is watching him, Max buries the tennis ball in the trunk here. Max then goes to the kitchen to get a cookie.

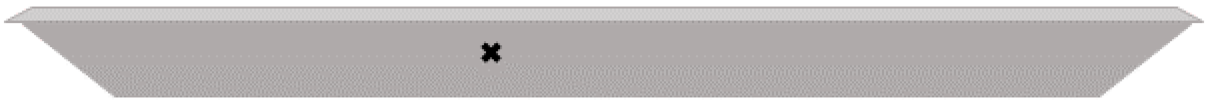

Page Break

Q18

While Max is in the kitchen, Audrey digs the tennis ball out and hides it here. She puts everything else in the trunk back where it was so it looks undisturbed.

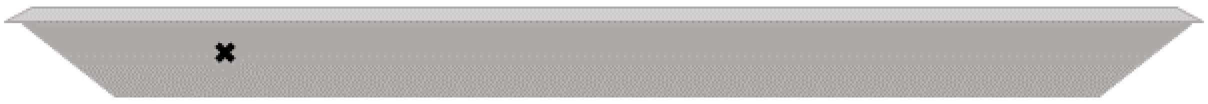

Page Break

Q19
Now try to find as many words as you can in the puzzle below and type them in the field at the bottom of the page. You have 20 seconds!

____

____

____

____

____

Q20 Timing
First Click (1)
Last Click (2)
Page Submit (3)
Click Count (4)

Page Break

Q21
Please click on the image to answer.

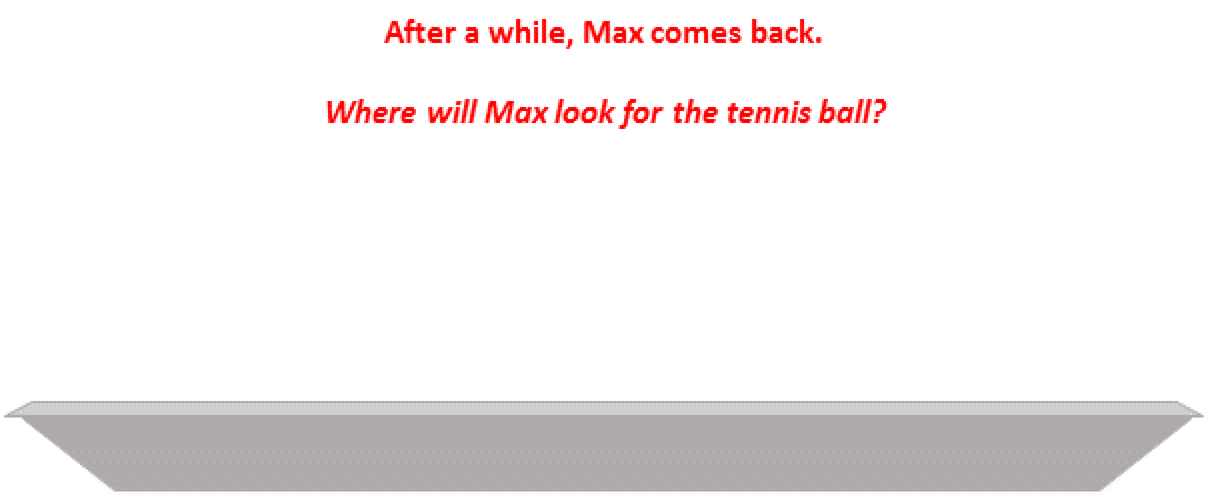

End of Block: Block3

Start of Block: Block4

Q26

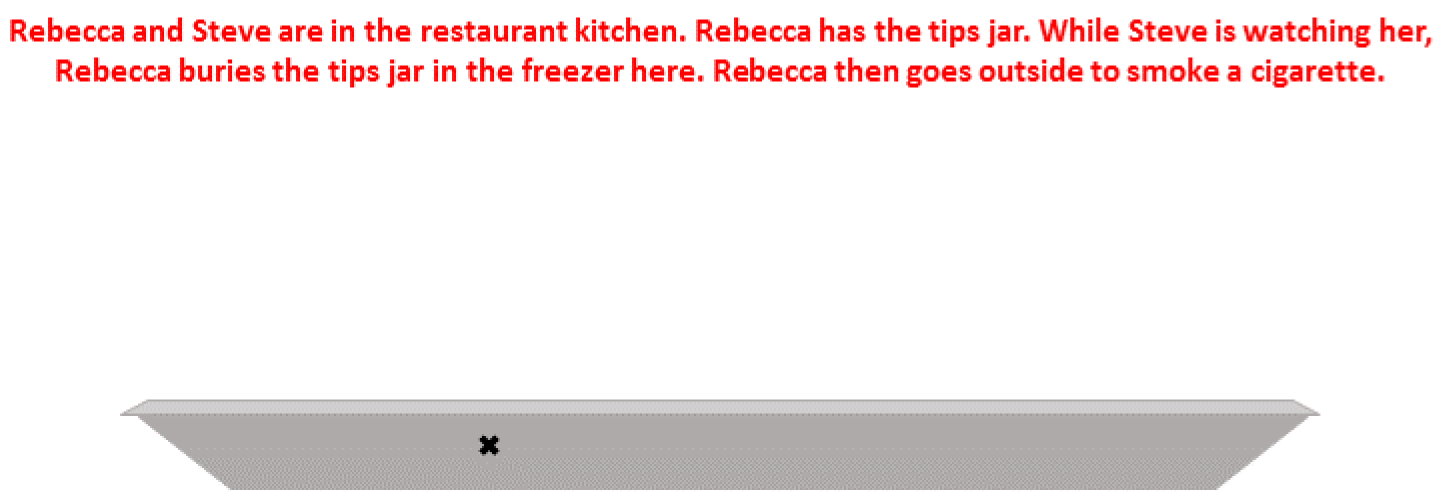

Page Break

Q27

While Rebecca is outside, Steve digs the tips jar out and hides it here. He puts everything else in the freezer back where it was so it looks undisturbed.

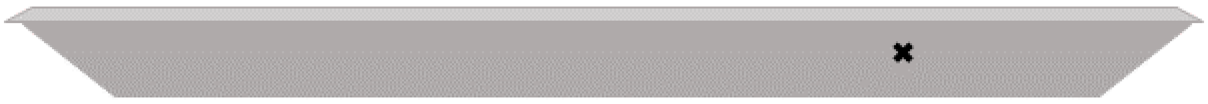

Page Break

Q28
Now try to find as many words as you can in the puzzle below and type them in the field at the bottom of the page. You have 20 seconds!

____

____

____

____

____

Q29 Timing
First Click (1)
Last Click (2)
Page Submit (3)
Click Count (4)

Page Break

Q30
Please click on the image to answer.

After a while, Rebecca comes back.

Where will Rebecca look for the tips jar?

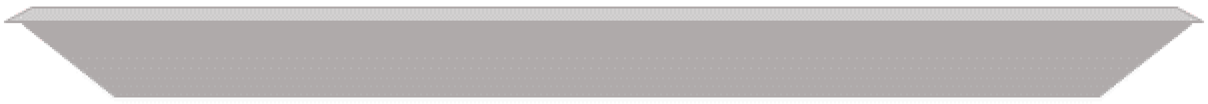

End of Block: Block4

Start of Block: Block5

Q31

Astrid and Luke are in the ball pit. Astrid has a chocolate egg. While Luke is watching her, Astrid buries the chocolate egg in the ball pit here.

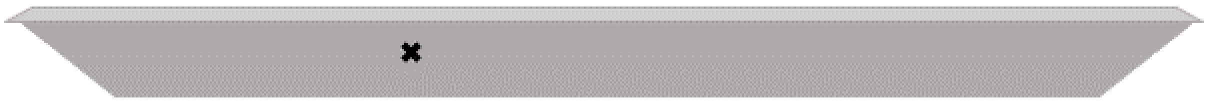

Page Break

Q32

While Luke is still watching her, Astrid digs the chocolate egg out and hides it here. She smooths over the balls so they look undisturbed. Astrid then goes inside to get a drink.

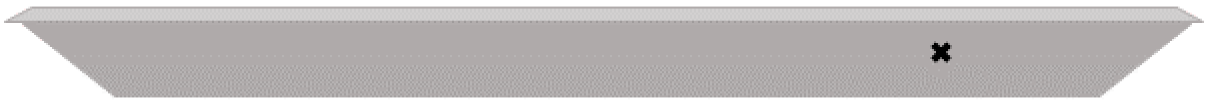

Page Break

Q33
Now try to find as many words as you can in the puzzle below and type them in the field at the bottom of the page. You have 20 seconds!

____

____

____

____

____

Q34 Timing
First Click (1)
Last Click (2)
Page Submit (3)
Click Count (4)

Page Break

Q35
Please click on the image to answer.

After a while, Astrid comes back.

Where is the chocolate egg?

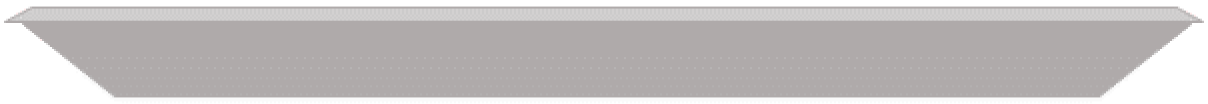

End of Block: Block5

Start of Block: Block6

Q36

Peter and Lisa are by the hotel garden pond. Peter has a bag of jewels. While Lisa is watching him, Peter buries the bag of jewels in the pond here. Peter then goes inside to make a call.

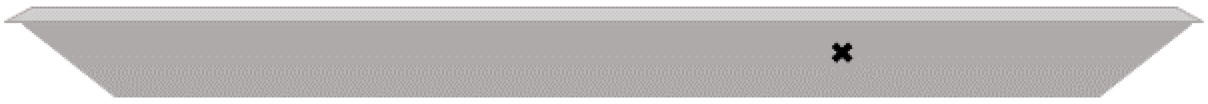

Page Break

Q37

While Peter is inside, Lisa digs the bag of jewels out and hides it here. She watches the surface of the pond until it looks undisturbed.

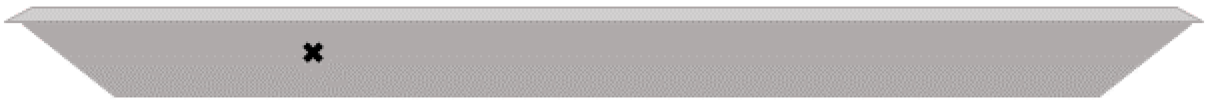

Page Break

Q38
Now try to find as many words as you can in the puzzle below and type them in the field at the bottom of the page. You have 20 seconds!

____

____

____

____

____

Q39 Timing
First Click (1)
Last Click (2)
Page Submit (3)
Click Count (4)

Page Break

Q40
Please click on the image to answer.

After a while, Peter comes back.

Where do you remember he buried the bag of jewels?

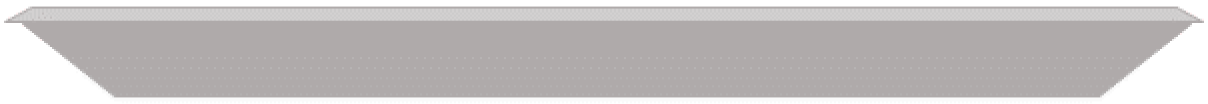

End of Block: Block6

Start of Block: Block7

Q41

Chloe and James are hiking in the snow. Chloe has a bottle of beer. While James is watching her, Chloe buries the bottle of beer in the snow here. Chloe then goes into their tent to check her emails.

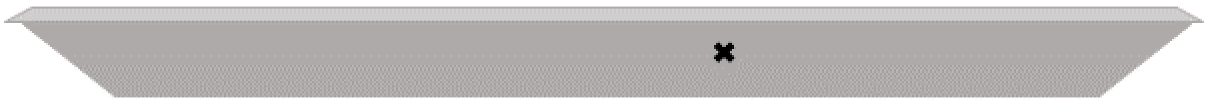

Page Break

Q42

While Chloe is away, James digs the bottle of beer out and hides it here. He smooths over the snow so it looks undisturbed.

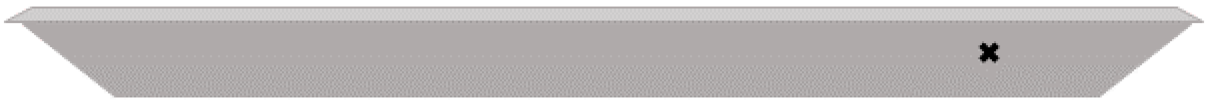

Page Break

Q43
Now try to find as many words as you can in the puzzle below and type them in the field at the bottom of the page. You have 20 seconds!

____

____

____

____

____

Q44 Timing
First Click (1)
Last Click (2)
Page Submit (3)
Click Count (4)

Page Break

Q45
Please click on the image to answer.

After a while, Chloe comes back.

Where do you remember she buried the bottle of beer?

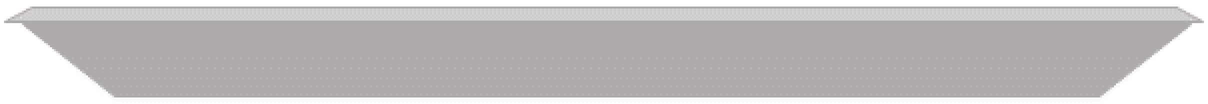

End of Block: Block7

Start of Block: Block8

Q46

Sarah and Alan are on the beach. Sarah has their passports. While Alan is watching her, Sarah buries the passports in the sand here. Sarah then goes to a shop to get some ice cream.

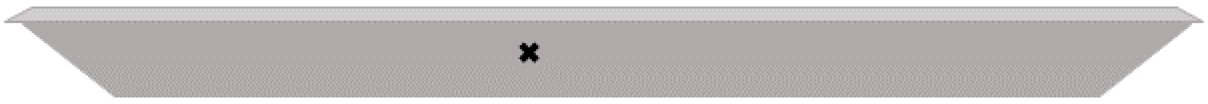

Page Break

Q47

While Sarah is away, Alan digs the passports out and hides them here. He smooths over the sand so it looks undisturbed.

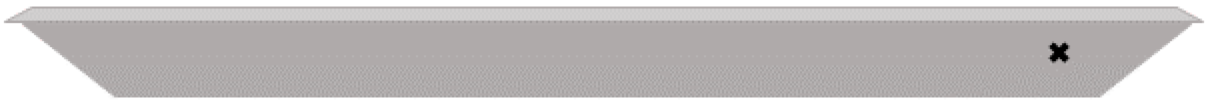

Page Break

Q48
Now try to find as many words as you can in the puzzle below and type them in the field at the bottom of the page. You have 20 seconds!

____

____

____

____

____

Q49 Timing
First Click (1)
Last Click (2)
Page Submit (3)
Click Count (4)

Page Break

Q50
Please click on the image to answer.

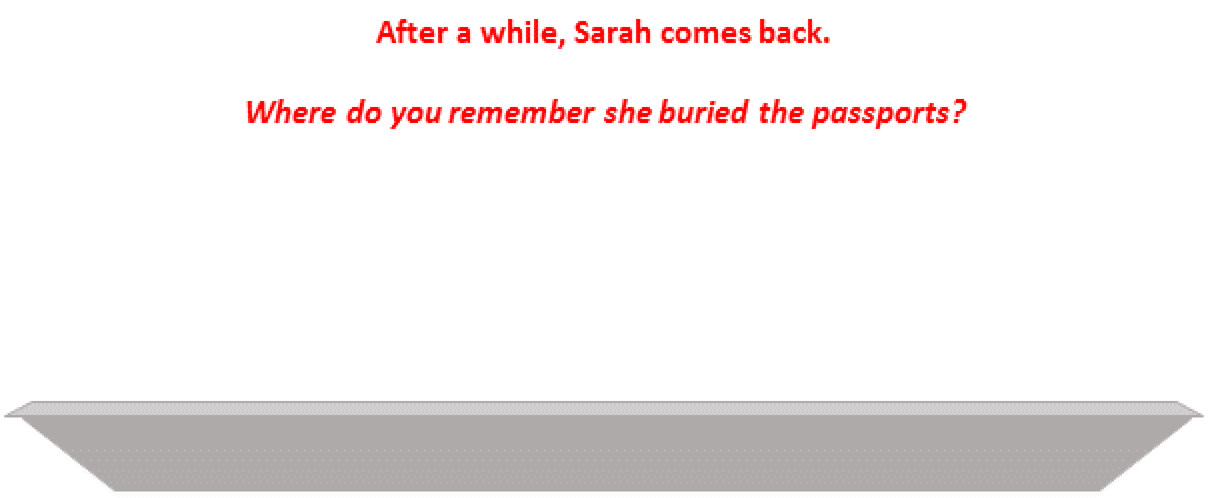

End of Block: Block8

Start of Block: Block9

Q51

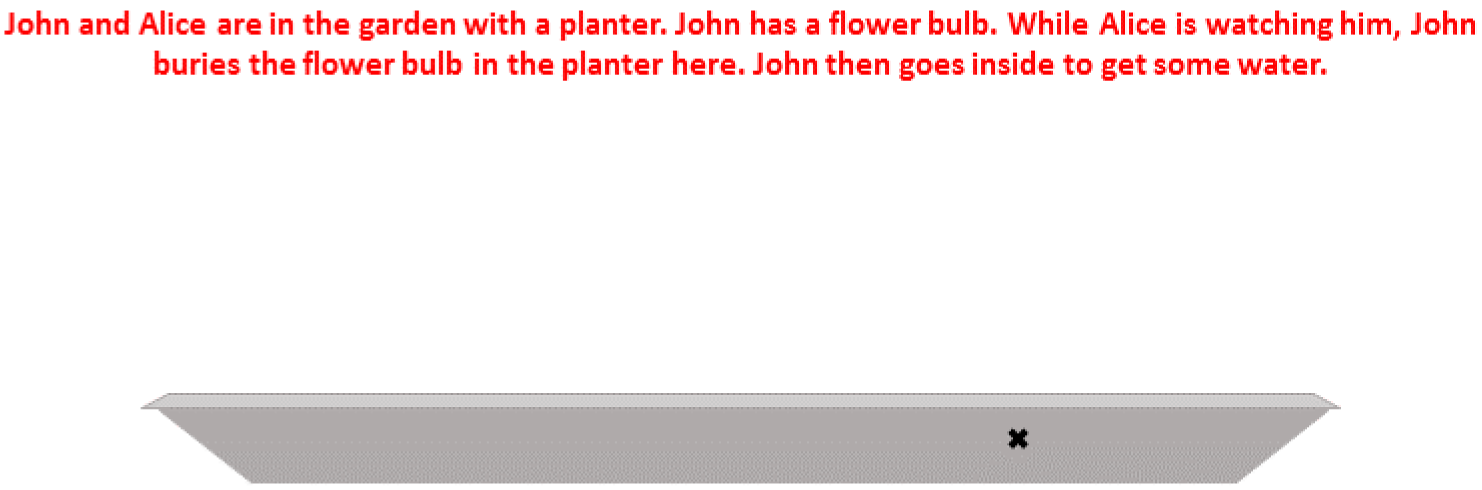

Page Break

Q52

While John is away, Alice digs the flower bulb out and hides it here. She smooths over the soil so it looks undisturbed.

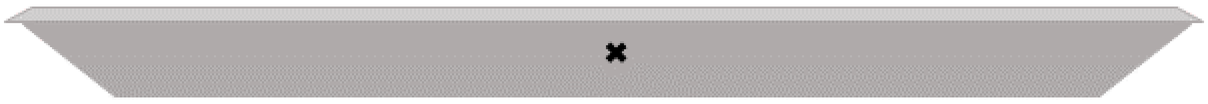

Page Break

Q53
Now try to find as many words as you can in the puzzle below and type them in the field at the bottom of the page. You have 20 seconds!

____

____

____

____

____

Q54 Timing
First Click (1)
Last Click (2)
Page Submit (3)
Click Count (4)

Page Break

Q55
Please click on the image to answer.

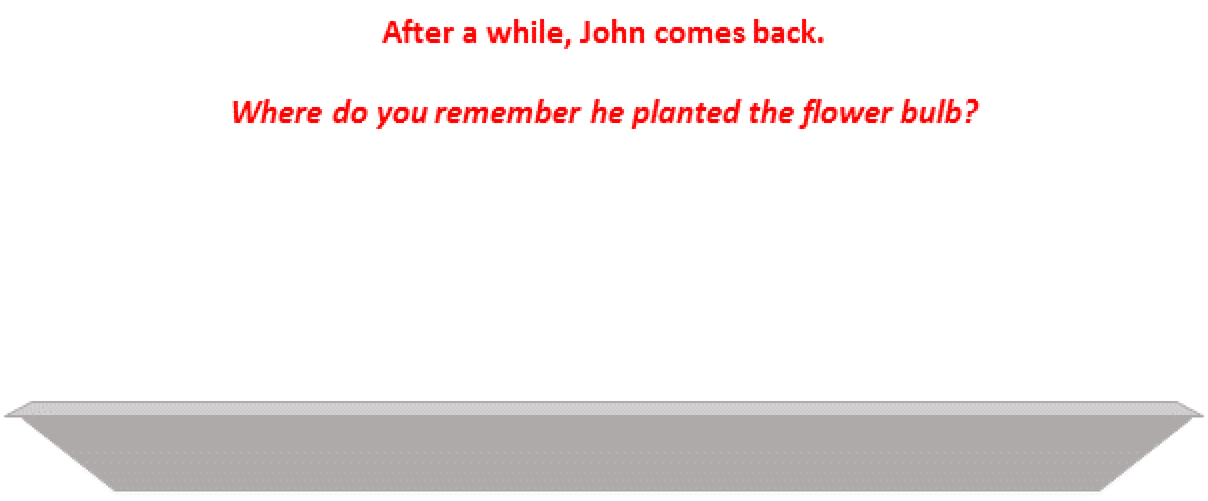

End of Block: Block9

Start of Block: Debrief

Q62 **Participant Debriefing Sheet:**

Thank you for taking part in our study.
What was the study about?
The experiment you took part in is part of a larger study designed to test how people use their own knowledge when having to take into account what others remember or believe. We know that adults tend to expect that other adults know what they themselves know. The critical component of this study was to see whether you were influenced by where you knew an object to be when you had to imagine where someone else remembered or believed it to be.

Page Break

Q62 ***Further Information*** *You are welcome to ask the researchers about the study.*

If you would like to know more about the study you can contact Dr. Steven Samuel (ss2391@cam.ac.uk) for further information.

Page Break

End of Block: Debrief

NEW sandbox: ownmem(you)-ownmem(did)

Start of Block: Prolific Info

Q65 Before you start, please:

– maximize your browser window;
– switch off phone/e-mail/music & anything else distracting
- and please enter your Prolific ID [it can be found at the top of this webpage or when going to your account info]:\\\

[To continue to the next question at any point in the survey please use the arrow button at the bottom right of the page]

____

End of Block: Prolific Info

Start of Block: Participant Info

Q54 Before you decide to take part in this study it is important for you to understand why the research is being done and what it will involve. Please take time to read the following information carefully and discuss it with others if you wish. A member of the team can be contacted if there is anything that is not clear or if you would like more information. Take time to decide whether or not you wish to take part.

Page Break

Q55 This study investigates how adults reason about what others want, believe and act. The study fits in with a larger research theme of how children and adults reason about other individuals' mental states and how both children and adults attribute beliefs and desires to other people.

Page Break

Q56 During the study you will be asked to make judgments about what others can see, hear or do. During the study you will be asked to make judgments based on stories you read. The experiment will take approximately 10 minutes to complete.

Page Break

Q57 Participation in the study is entirely voluntary and you can withdraw at any point. The results of the study may be written up or presented at conferences. Your participation will remain confidential and if individual data is presented there will be no means of identifying the individuals involved.

Page Break

Q58 If you would like to know more about the study or have any questions you can contact Steven Samuel (ss2391@cam.ac.uk) for further information.

The project has received ethical approval from the Psychology Ethics Committee of the University of Cambridge.

Page Break

End of Block: Participant Info

Start of Block: Consent Page 1/6

Q81 I confirm that I have read and understand the participant information.

○ Yes (1)

○ No (2)

Q93 I have had the opportunity to ask questions and had them answered.

○ Yes (1)

○ No (2)

Q105 I understand that all personal information will remain confidential and that all efforts will be made to ensure that I cannot be identified (except as might be required by law).

○ Yes (1)

○ No (2)

Q117 I agree that data gathered in this study may be stored anonymously and securely, and may be used for future research.

○ Yes (1)

○ No (2)

Q129 I understand that my participation is voluntary and that I am free to withdraw at any time without giving a reason.

○ Yes (1)

○ No (2)

Q141 I agree to take part in this study.

○ Yes (1)

○ No (2)

End of Block: Consent Page 1/6

Start of Block: Consent Page 2/6

Q66 There are two parts to the study.

First you will be asked to click on various locations on your screen (this helps us with calibration).

Second (the main part of the study) you will read some short stories about objects being hidden. Please pay careful attention to the story and the accompanying visual information. After each story you will have 20 seconds to find words in a wordsearch. After each wordsearch you will be asked a question about the story that you have just read. Total experiment time is approximately 10 minutes.

The calibration process is next...

End of Block: Consent Page 2/6

Start of Block: Consent Page 3/6

Start of Block: Consent Page 4/6

Start of Block: Consent Page 5/6

Start of Block: Consent Page 6/6

Start of Block: Start

Q17

Please click right in the centre of the left cross (first) and right in the centre of the right cross (second). Try to be as accurate as possible. If the mouse-controlled cross does not align perfectly with the crosses on the screen (for example it always has to be a fraction to the left or right), please do the same for each cross. Make sure a marker is left on each cross before moving on to the next page. You can try as many times as you like.

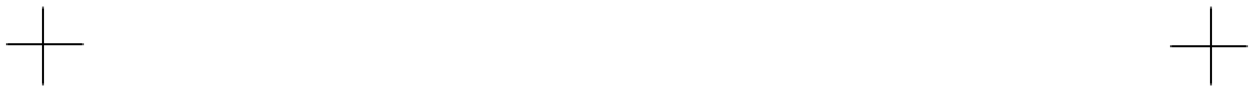

Page Break

Q48

Now please click right in the centre of this left cross and right in the centre of this right cross. Try to be as accurate as possible, and again if the mouse cannot fall exactly in the centre please ensure you do the same for each click. You can try as many times as you like.

Page Break

Q49
And finally, please do the same for the two crosses on this page.

Page Break

Q47 Browser Meta Info
Browser (1)
Version (2)
Operating System (3)
Screen Resolution (4)
Flash Version (5)
Java Support (6)
User Agent (7)

End of Block: Start

Start of Block: Block1

Q1

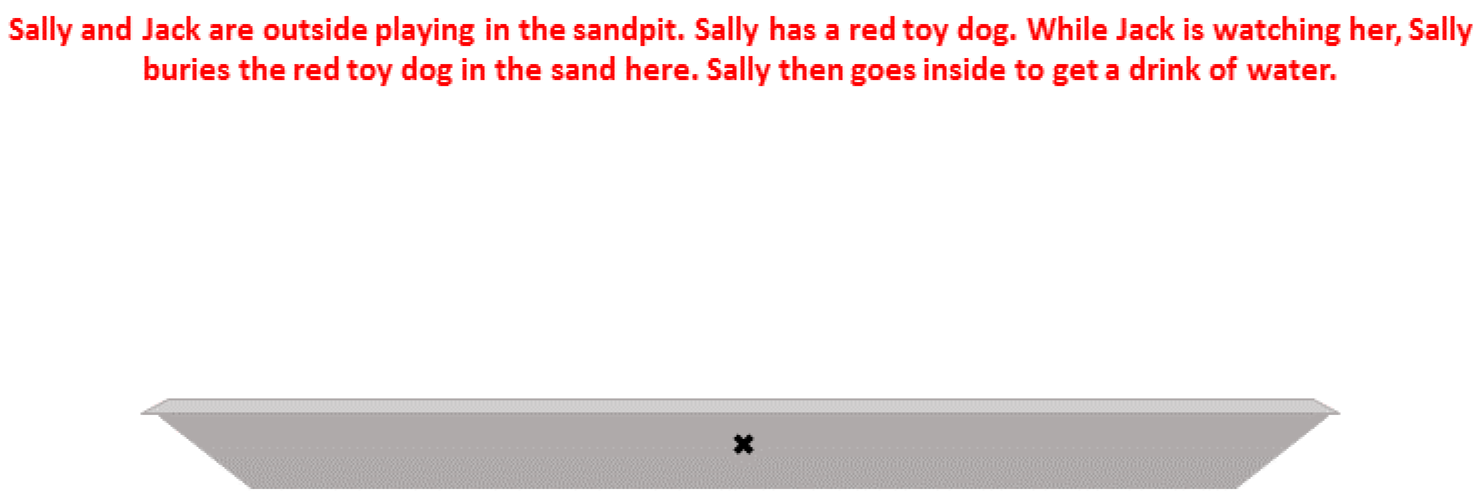

Page Break

Q2

While Sally is inside the house, Jack digs the red toy dog out and hides it here. He smooths over the sand so it looks undisturbed.

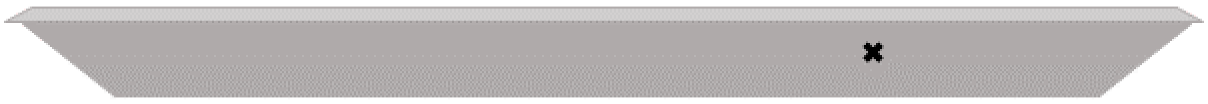

Page Break

Q3
Now try to find as many words as you can in the puzzle below and type them in the field at the bottom of the page. You have 20 seconds!

____

____

____

____

____

Q5 Timing
First Click (1)
Last Click (2)
Page Submit (3)
Click Count (4)

Page Break

Q4
Please click on the image to answer.

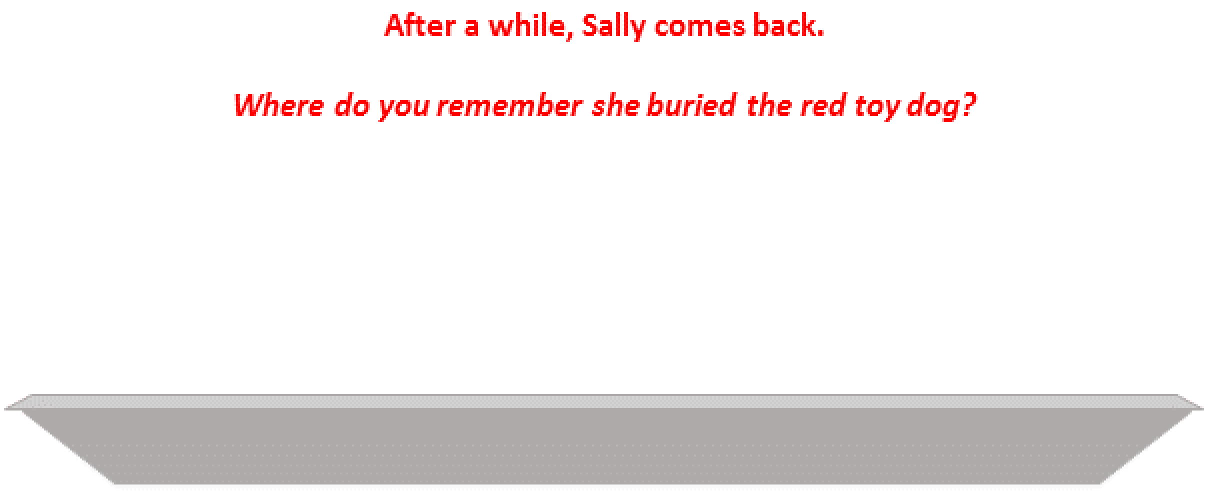

End of Block: Block1

Start of Block: Block2

Q12

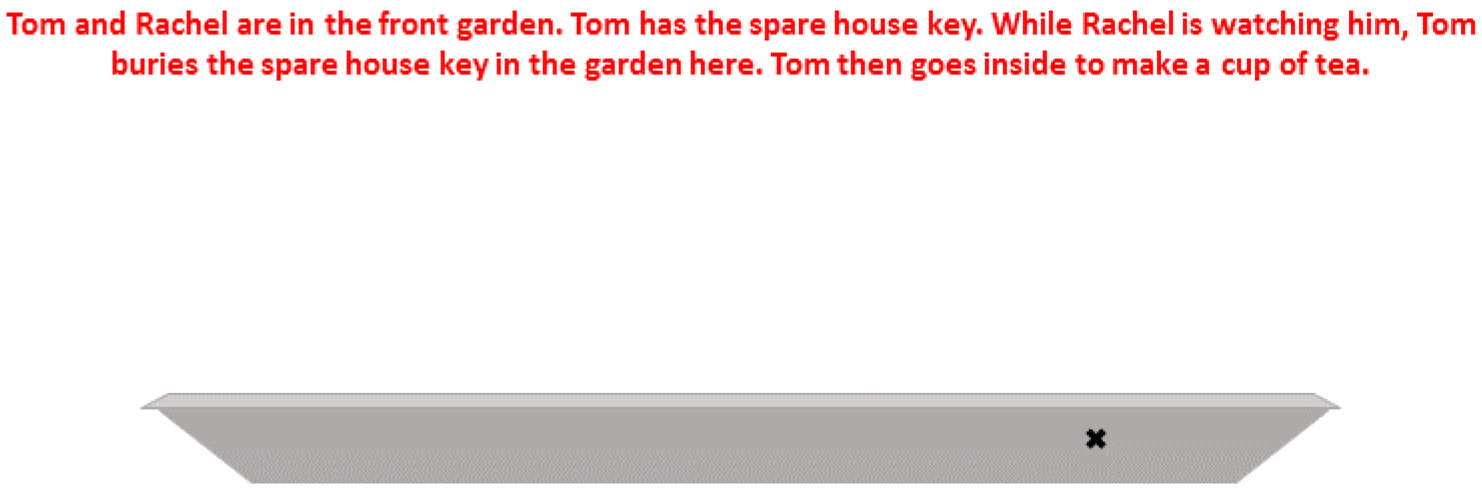

Page Break

Q13

While Tom is inside the house, Rachel digs the spare house key out and hides it here. She smooths over the earth so it looks undisturbed.

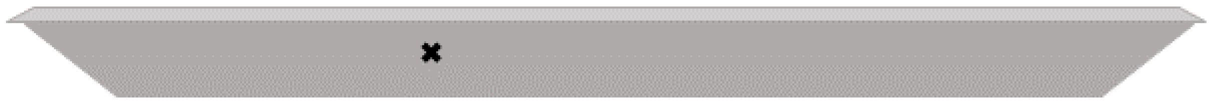

Page Break

Q14
Now try to find as many words as you can in the puzzle below and type them in the field at the bottom of the page. You have 20 seconds!

____

____

____

____

____

Q15 Timing
First Click (1)
Last Click (2)
Page Submit (3)
Click Count (4)

Page Break

Q16
Please click on the image to answer.

After a while, Tom comes back.

Where do you remember he buried the spare house key?

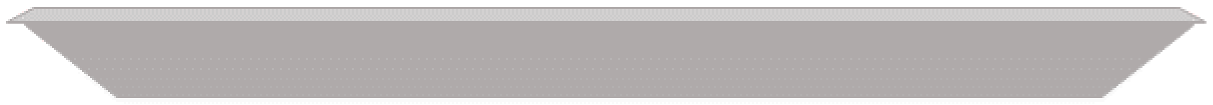

End of Block: Block2

Start of Block: Block3

Q17

Max and Audrey are putting toys in the big toy trunk in the living room. Max has a tennis ball. While Audrey is watching him, Max buries the tennis ball in the trunk here. Max then goes to the kitchen to get a cookie.

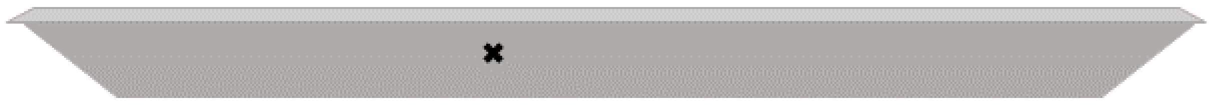

Page Break

Q18

While Max is in the kitchen, Audrey digs the tennis ball out and hides it here. She puts everything else in the trunk back where it was so it looks undisturbed.

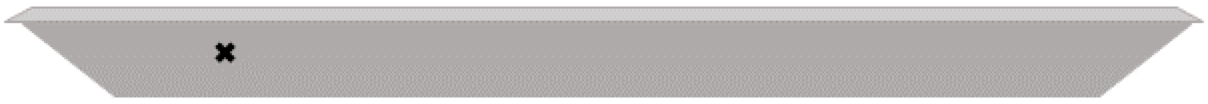

Page Break

Q19
Now try to find as many words as you can in the puzzle below and type them in the field at the bottom of the page. You have 20 seconds!

____

____

____

____

____

Q20 Timing
First Click (1)
Last Click (2)
Page Submit (3)
Click Count (4)

Page Break

Q21
Please click on the image to answer.

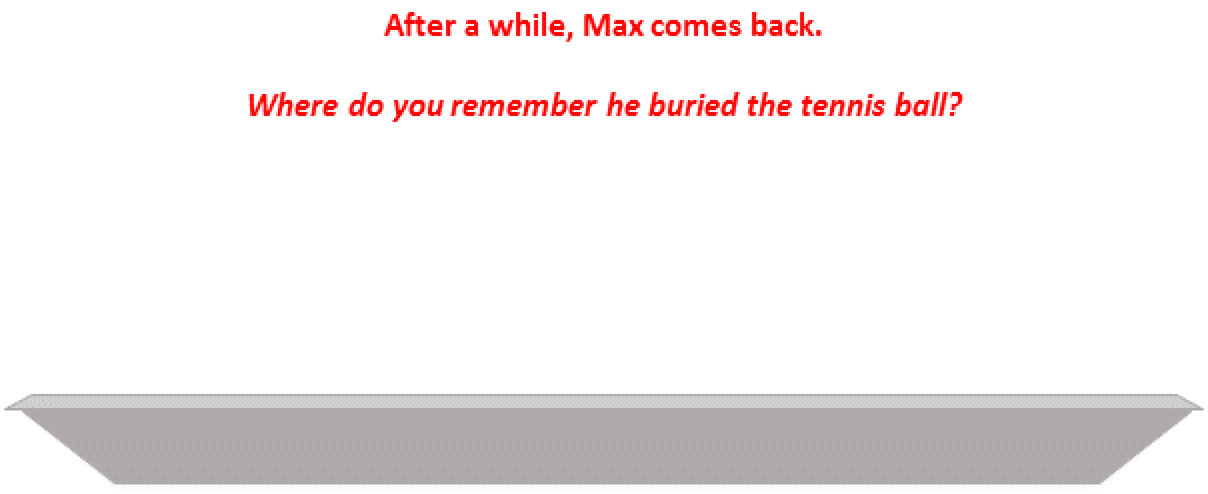

End of Block: Block3

Start of Block: Block4

Q26

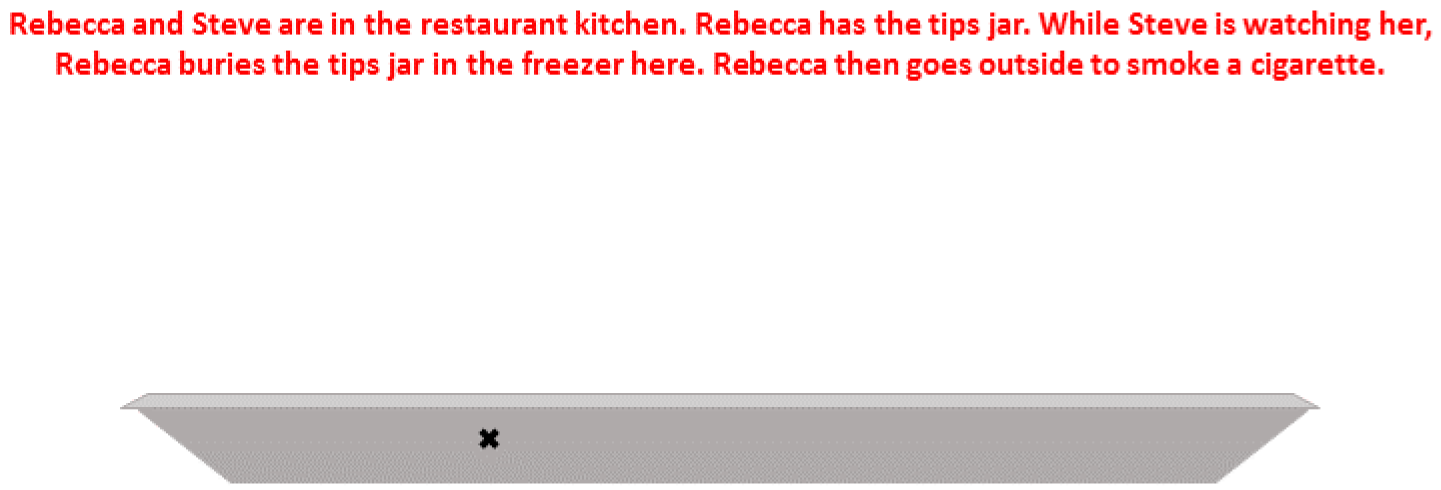

Page Break

Q27

While Rebecca is outside, Steve digs the tips jar out and hides it here. He puts everything else in the freezer back where it was so it looks undisturbed.

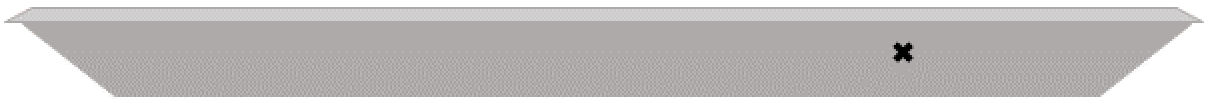

Page Break

Q28
Now try to find as many words as you can in the puzzle below and type them in the field at the bottom of the page. You have 20 seconds!

____

____

____

____

____

Q29 Timing
First Click (1)
Last Click (2)
Page Submit (3)
Click Count (4)

Page Break

Q30
Please click on the image to answer.

After a while, Rebecca comes back.

Where do you remember she buried the tips jar?

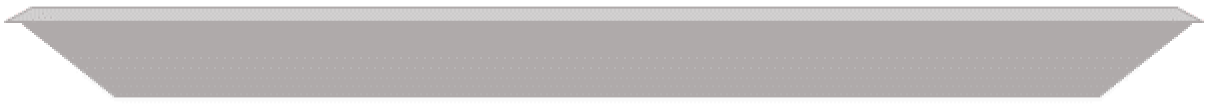

End of Block: Block4

Start of Block: Block5

Q31

Astrid and Luke are in the ball pit. Astrid has a chocolate egg. While Luke is watching her, Astrid buries the chocolate egg in the ball pit here.

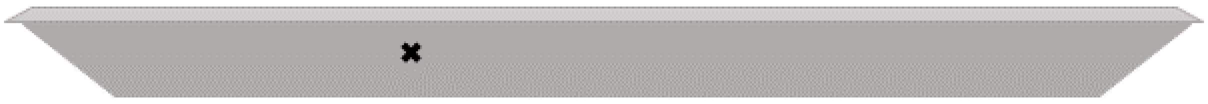

Page Break

Q32

While Luke is still watching her, Astrid digs the chocolate egg out and hides it here. She smooths over the balls so they look undisturbed. Astrid then goes inside to get a drink.

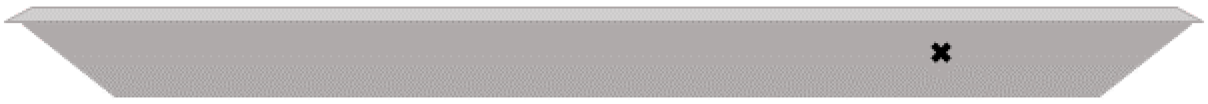

Page Break

Q33
Now try to find as many words as you can in the puzzle below and type them in the field at the bottom of the page. You have 20 seconds!

____

____

____

____

____

Q34 Timing
First Click (1)
Last Click (2)
Page Submit (3)
Click Count (4)

Page Break

Q35
Please click on the image to answer.

After a while, Astrid comes back.

Where is the chocolate egg?

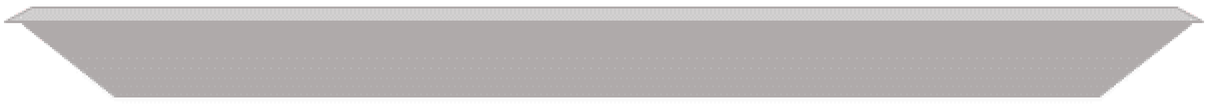

End of Block: Block5

Start of Block: Block6

Q36

Peter and Lisa are by the hotel garden pond. Peter has a bag of jewels. While Lisa is watching him, Peter buries the bag of jewels in the pond here. Peter then goes inside to make a call.

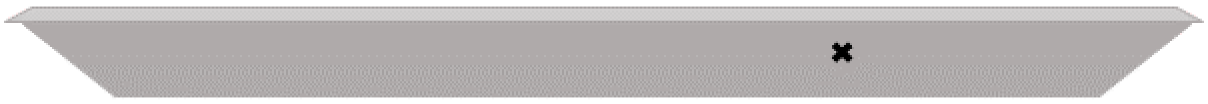

Page Break

Q37

While Peter is inside, Lisa digs the bag of jewels out and hides it here. She watches the surface of the pond until it looks undisturbed.

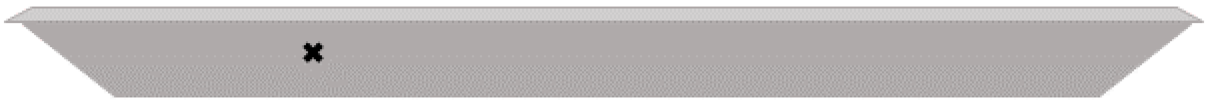

Page Break

Q38
Now try to find as many words as you can in the puzzle below and type them in the field at the bottom of the page. You have 20 seconds!

____

____

____

____

____

Q39 Timing
First Click (1)
Last Click (2)
Page Submit (3)
Click Count (4)

Page Break

Q40
Please click on the image to answer.

After a while, Peter comes back.

Where did he bury the bag of jewels?

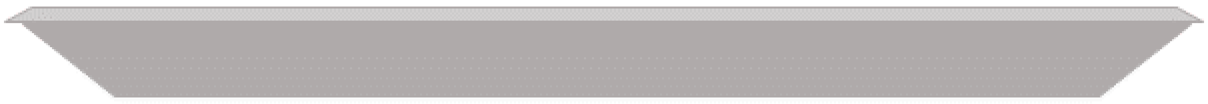

End of Block: Block6

Start of Block: Block7

Q41

Chloe and James are hiking in the snow. Chloe has a bottle of beer. While James is watching her, Chloe buries the bottle of beer in the snow here. Chloe then goes into their tent to check her emails.

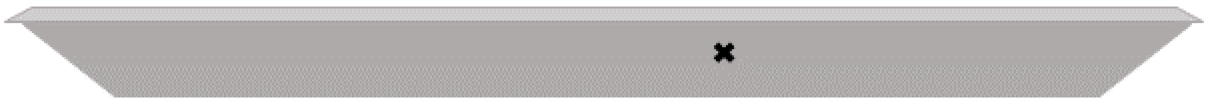

Page Break

Q42

While Chloe is away, James digs the bottle of beer out and hides it here. He smooths over the snow so it looks undisturbed.

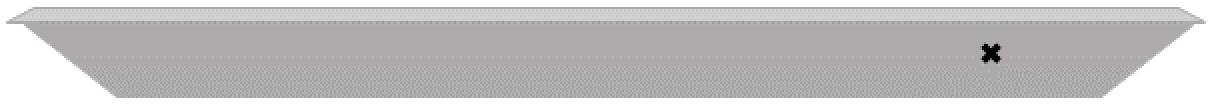

Page Break

Q43
Now try to find as many words as you can in the puzzle below and type them in the field at the bottom of the page. You have 20 seconds!

____

____

____

____

____

Q44 Timing
First Click (1)
Last Click (2)
Page Submit (3)
Click Count (4)

Page Break

Q45
Please click on the image to answer.

After a while, Chloe comes back.

Where did she bury the bottle of beer?

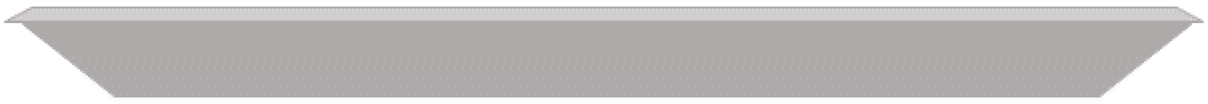

End of Block: Block7

Start of Block: Block8

Q46

Sarah and Alan are on the beach. Sarah has their passports. While Alan is watching her, Sarah buries the passports in the sand here. Sarah then goes to a shop to get some ice cream.

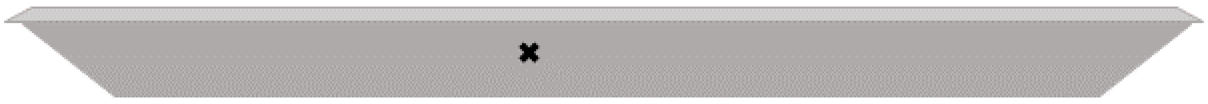

Page Break

Q47

While Sarah is away, Alan digs the passports out and hides them here. He smooths over the sand so it looks undisturbed.

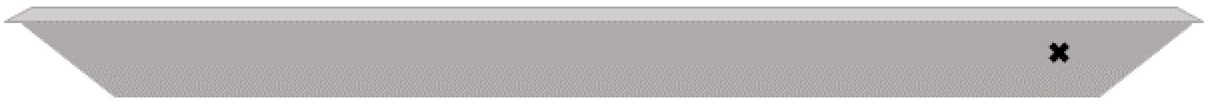

Page Break

Q48
Now try to find as many words as you can in the puzzle below and type them in the field at the bottom of the page. You have 20 seconds!

____

____

____

____

____

Q49 Timing
First Click (1)
Last Click (2)
Page Submit (3)
Click Count (4)

Page Break

Q50
Please click on the image to answer.

After a while, Sarah comes back.

Where did she bury the passports?

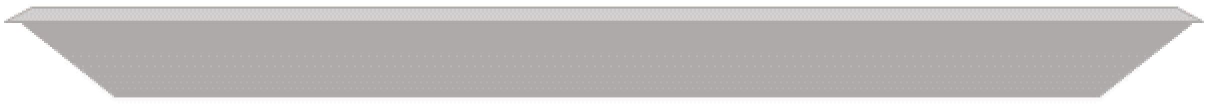

End of Block: Block8

Start of Block: Block9

Q51

John and Alice are in the garden with a planter. John has a flower bulb. While Alice is watching him, John buries the flower bulb in the planter here. John then goes inside to get some water.

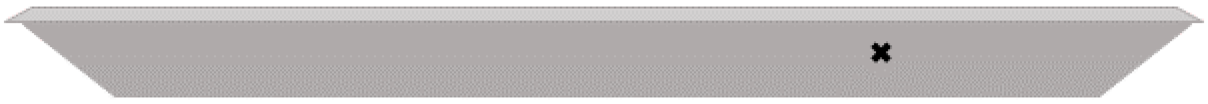

Page Break

Q52

While John is away, Alice digs the flower bulb out and hides it here. She smooths over the soil so it looks undisturbed.

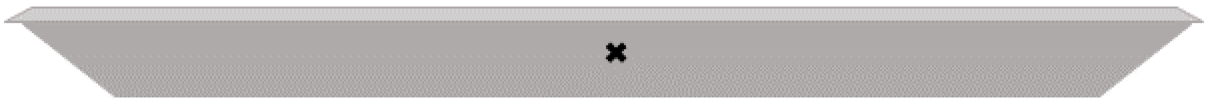

Page Break

Q53
Now try to find as many words as you can in the puzzle below and type them in the field at the bottom of the page. You have 20 seconds!

____

____

____

____

____

Q54 Timing
First Click (1)
Last Click (2)
Page Submit (3)
Click Count (4)

Page Break

Q55
Please click on the image to answer.

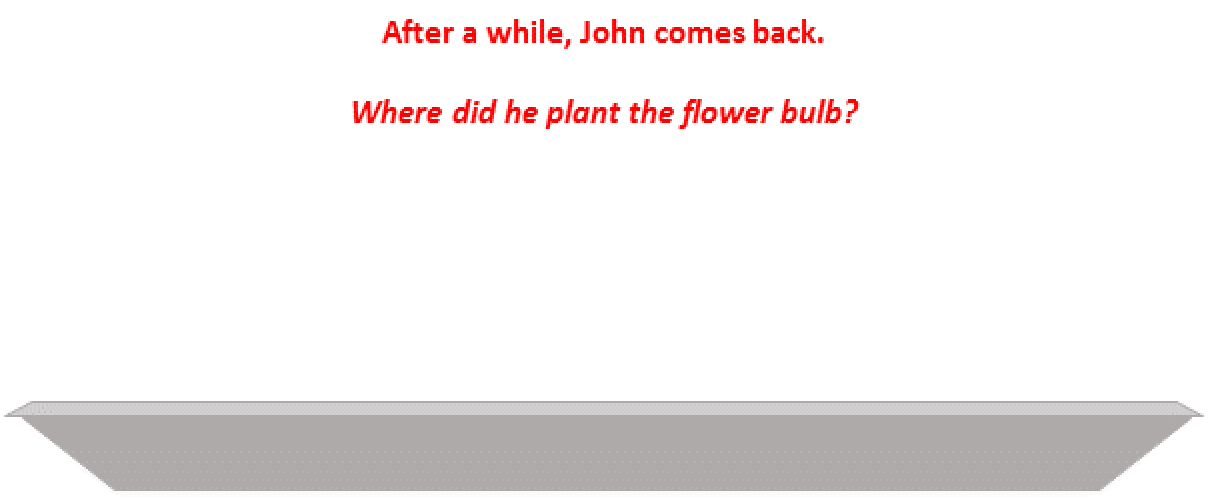

End of Block: Block9

Start of Block: Debrief

Q62 **Participant Debriefing Sheet:**

Thank you for taking part in our study.
What was the study about?
The experiment you took part in is part of a larger study designed to test how people use their own knowledge when having to take into account what others remember or believe. We know that adults tend to expect that other adults know what they themselves know. The critical component of this study was to see whether you were influenced by where you knew an object to be when you had to imagine where someone else remembered or believed it to be.

Page Break

Q62 ***Further Information*** *You are welcome to ask the researchers about the study.*

If you would like to know more about the study you can contact Dr. Steven Samuel (ss2391@cam.ac.uk) for further information. Click here to confirm you have completed the survey.

Page Break

End of Block: Debrief

NEW sandbox: ownmem(did)-ownmem(you)

Start of Block: Prolific Info

Q65 Before you start, please:

– maximize your browser window;
– switch off phone/e-mail/music & anything else distracting
- and please enter your Prolific ID [it can be found at the top of this webpage or when going to your account info]:\\\

[To continue to the next question at any point in the survey please use the arrow button at the bottom right of the page]

____

End of Block: Prolific Info

Start of Block: Participant Info

Q54 Before you decide to take part in this study it is important for you to understand why the research is being done and what it will involve. Please take time to read the following information carefully and discuss it with others if you wish. A member of the team can be contacted if there is anything that is not clear or if you would like more information. Take time to decide whether or not you wish to take part.

Page Break

Q55 This study investigates how adults reason about what others want, believe and act. The study fits in with a larger research theme of how children and adults reason about other individuals' mental states and how both children and adults attribute beliefs and desires to other people.

Page Break

Q56 During the study you will be asked to make judgments based on stories you read. The experiment will take approximately 10 minutes to complete.

Page Break

Q57 Participation in the study is entirely voluntary and you can withdraw at any point. The results of the study may be written up or presented at conferences. Your participation will remain confidential and if individual data is presented there will be no means of identifying the individuals involved.

Page Break

Q58 If you would like to know more about the study or have any questions you can contact Steven Samuel (ss2391@cam.ac.uk) for further information.

The project has received ethical approval from the Psychology Ethics Committee of the University of Cambridge.

Page Break

End of Block: Participant Info

Start of Block: Consent Page 1/6

Q81 I confirm that I have read and understand the participant information.

- Yes (1)
- No (2)

Q93 I have had the opportunity to ask questions and had them answered.

- Yes (1)
- No (2)

Q105 I understand that all personal information will remain confidential and that all efforts will be made to ensure that I cannot be identified (except as might be required by law).

- Yes (1)
- No (2)

Q117 I agree that data gathered in this study may be stored anonymously and securely, and may be used for future research.

- Yes (1)
- No (2)

Q129 I understand that my participation is voluntary and that I am free to withdraw at any time without giving a reason.

- Yes (1)
- No (2)

Q141 I agree to take part in this study.

- Yes (1)
- No (2)

End of Block: Consent Page 1/6

Start of Block: Consent Page 2/6

Q66 There are two parts to the study.

First you will be asked to click on various locations on your screen (this helps us with calibration).

Second (the main part of the study) you will read some short stories about objects being hidden. Please pay careful attention to the story and the accompanying visual information. After each story you will have 20 seconds to find words in a wordsearch. After each wordsearch you will be asked a question about the story that you have just read. Total experiment time is approximately 10 minutes.

The calibration process is next...

End of Block: Consent Page 2/6

Start of Block: Consent Page 3/6

Start of Block: Consent Page 4/6

Start of Block: Consent Page 5/6

Start of Block: Consent Page 6/6

Start of Block: Start

Q17
Please click right in the centre of the left cross (first) and right in the centre of the right cross (second). Try to be as accurate as possible. If the mouse-controlled cross does not align perfectly with the crosses on the screen (for example it always has to be a fraction to the left or right), please do the same for each cross. Make sure a marker is left on each cross before moving on to the next page. You can try as many times as you like.

Page Break

Q48
Now please click right in the centre of this left cross and right in the centre of this right cross. Try to be as accurate as possible, and again if the mouse cannot fall exactly in the centre please ensure you do the same for each click. You can try as many times as you like.

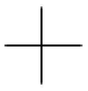

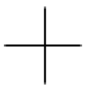

Page Break

Q49
And finally, please do the same for the two crosses on this page.

Page Break

Q47 Browser Meta Info
Browser (1)
Version (2)
Operating System (3)
Screen Resolution (4)
Flash Version (5)
Java Support (6)
User Agent (7)

End of Block: Start

Start of Block: Block1

Q1

Sally and Jack are outside playing in the sandpit. Sally has a red toy dog. While Jack is watching her, Sally buries the red toy dog in the sand here. Sally then goes inside to get a drink of water.

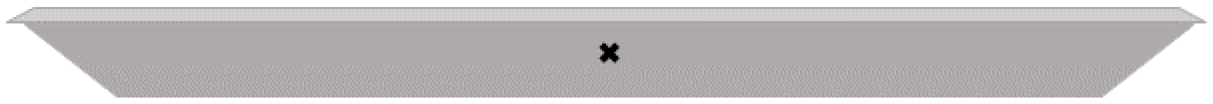

Page Break

Q2

While Sally is inside the house, Jack digs the red toy dog out and hides it here. He smooths over the sand so it looks undisturbed.

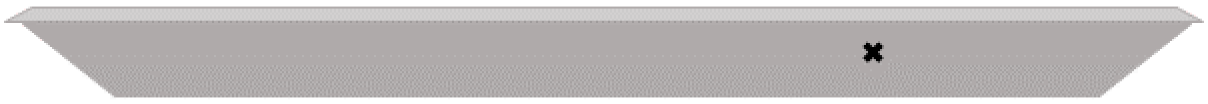

Page Break

Q3
Now try to find as many words as you can in the puzzle below and type them in the field at the bottom of the page. You have 20 seconds!

____

____

____

____

____

Q5 Timing
First Click (1)
Last Click (2)
Page Submit (3)
Click Count (4)

Page Break

Q4
Please click on the image to answer.

After a while, Sally comes back.

Where did she bury the red toy dog?

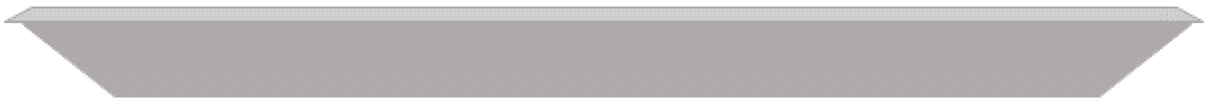

End of Block: Block1

Start of Block: Block2

Q12

Tom and Rachel are in the front garden. Tom has the spare house key. While Rachel is watching him, Tom buries the spare house key in the garden here. Tom then goes inside to make a cup of tea.

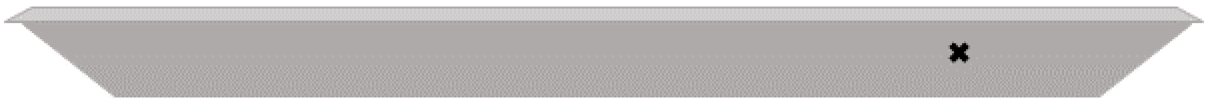

Page Break

Q13

While Tom is inside the house, Rachel digs the spare house key out and hides it here. She smooths over the earth so it looks undisturbed.

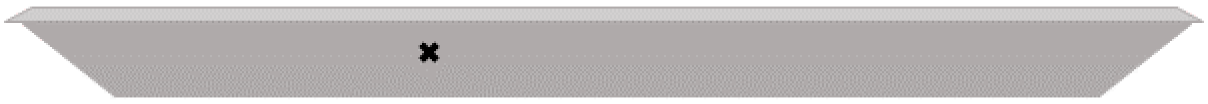

Page Break

Q14
Now try to find as many words as you can in the puzzle below and type them in the field at the bottom of the page. You have 20 seconds!

____

____

____

____

____

Q15 Timing
First Click (1)
Last Click (2)
Page Submit (3)
Click Count (4)

Page Break

Q16
Please click on the image to answer.

After a while, Tom comes back.

Where did he bury the spare house key?

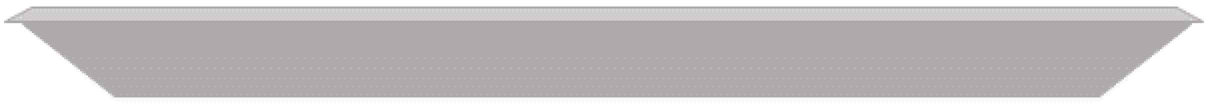

End of Block: Block2

Start of Block: Block3

Q17

Max and Audrey are putting toys in the big toy trunk in the living room. Max has a tennis ball. While Audrey is watching him, Max buries the tennis ball in the trunk here. Max then goes to the kitchen to get a cookie.

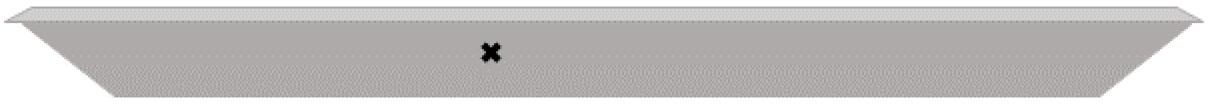

Page Break

Q18

While Max is in the kitchen, Audrey digs the tennis ball out and hides it here. She puts everything else in the trunk back where it was so it looks undisturbed.

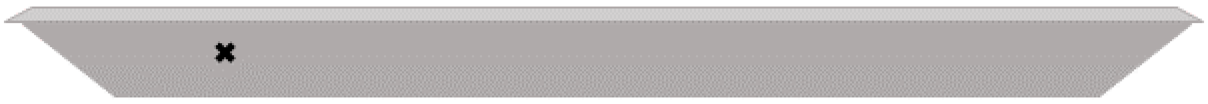

Page Break

Q19
Now try to find as many words as you can in the puzzle below and type them in the field at the bottom of the page. You have 20 seconds!

____

____

____

____

____

Q20 Timing
First Click (1)
Last Click (2)
Page Submit (3)
Click Count (4)

Page Break

Q21
Please click on the image to answer.

After a while, Max comes back.

Where did he bury the tennis ball?

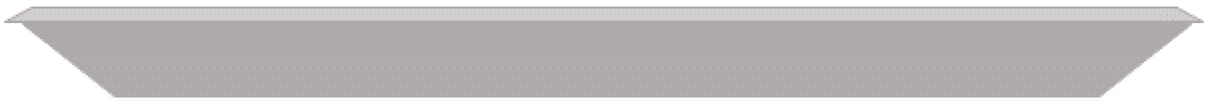

End of Block: Block3

Start of Block: Block4

Q26

Rebecca and Steve are in the restaurant kitchen. Rebecca has the tips jar. While Steve is watching her, Rebecca buries the tips jar in the freezer here. Rebecca then goes outside to smoke a cigarette.

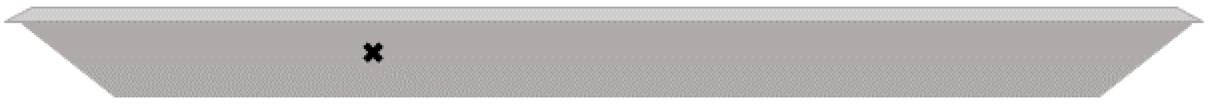

Page Break

Q27

While Rebecca is outside, Steve digs the tips jar out and hides it here. He puts everything else in the freezer back where it was so it looks undisturbed.

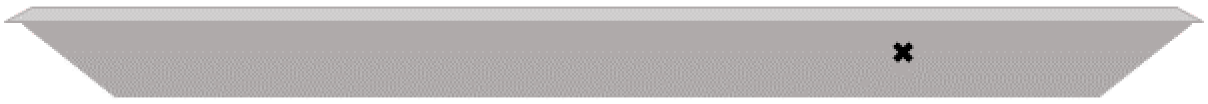

Page Break

Q28
Now try to find as many words as you can in the puzzle below and type them in the field at the bottom of the page. You have 20 seconds!

____

____

____

____

____

Q29 Timing
First Click (1)
Last Click (2)
Page Submit (3)
Click Count (4)

Page Break

Q30
Please click on the image to answer.

After a while, Rebecca comes back.

Where did she bury the tips jar?

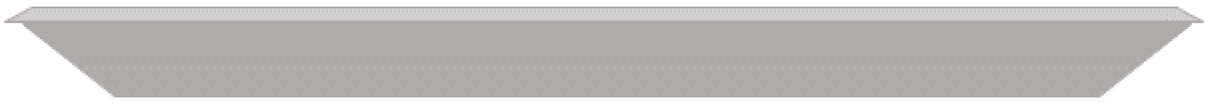

End of Block: Block4

Start of Block: Block5

Q31

Astrid and Luke are in the ball pit. Astrid has a chocolate egg. While Luke is watching her, Astrid buries the chocolate egg in the ball pit here.

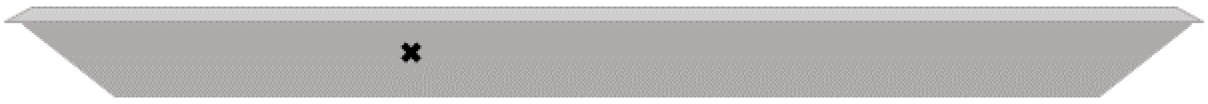

Page Break

Q32

While Luke is still watching her, Astrid digs the chocolate egg out and hides it here. She smooths over the balls so they look undisturbed. Astrid then goes inside to get a drink.

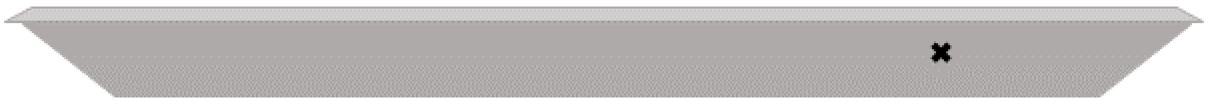

Page Break

Q33
Now try to find as many words as you can in the puzzle below and type them in the field at the bottom of the page. You have 20 seconds!

____

____

____

____

____

Q34 Timing
First Click (1)
Last Click (2)
Page Submit (3)
Click Count (4)

Page Break

Q35
Please click on the image to answer.

After a while, Astrid comes back.

Where is the chocolate egg?

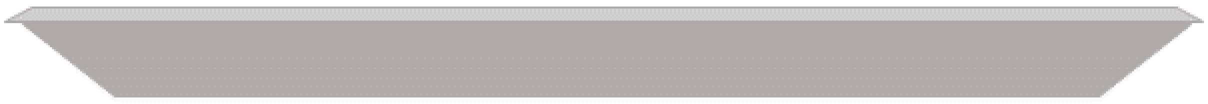

End of Block: Block5

Start of Block: Block6

Q36

Peter and Lisa are by the hotel garden pond. Peter has a bag of jewels. While Lisa is watching him, Peter buries the bag of jewels in the pond here. Peter then goes inside to make a call.

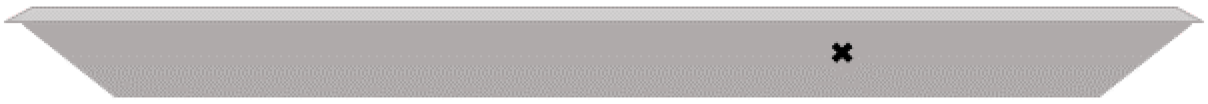

Page Break

Q37

While Peter is inside, Lisa digs the bag of jewels out and hides it here. She watches the surface of the pond until it looks undisturbed.

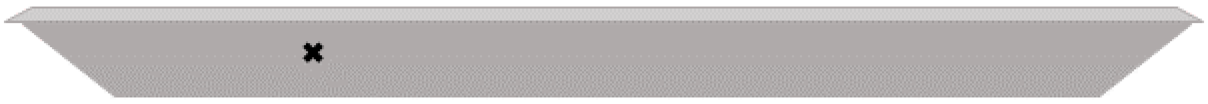

Page Break

Q38
Now try to find as many words as you can in the puzzle below and type them in the field at the bottom of the page. You have 20 seconds!

____

____

____

____

____

Q39 Timing
First Click (1)
Last Click (2)
Page Submit (3)
Click Count (4)

Page Break

Q40
Please click on the image to answer.

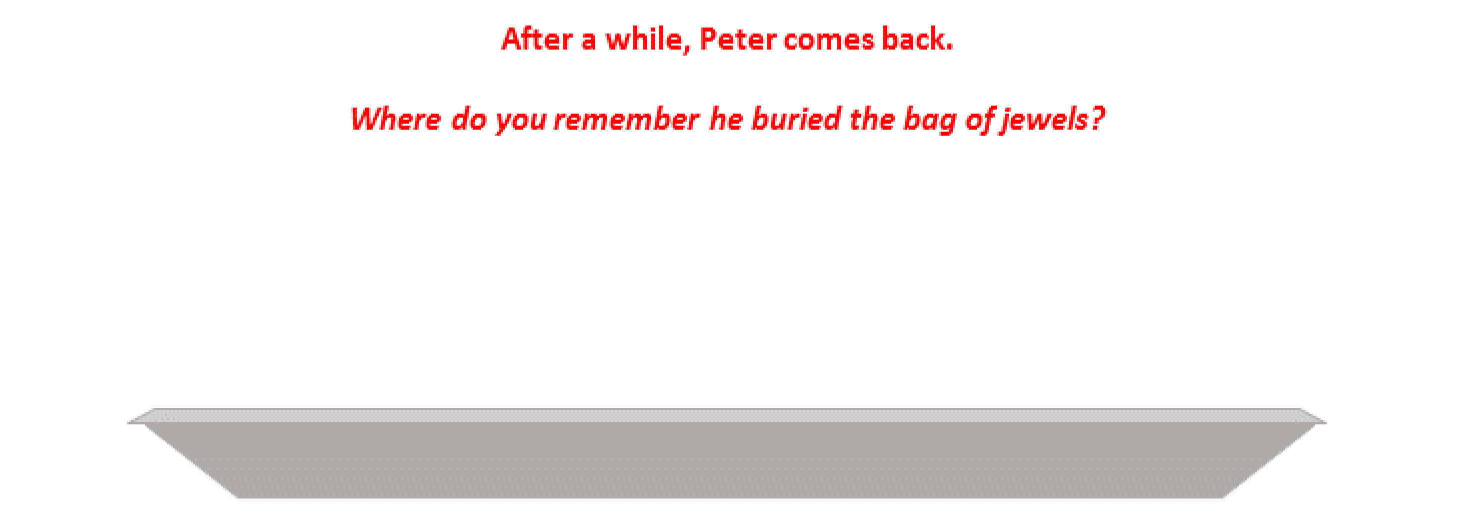

End of Block: Block6

Start of Block: Block7

Q41

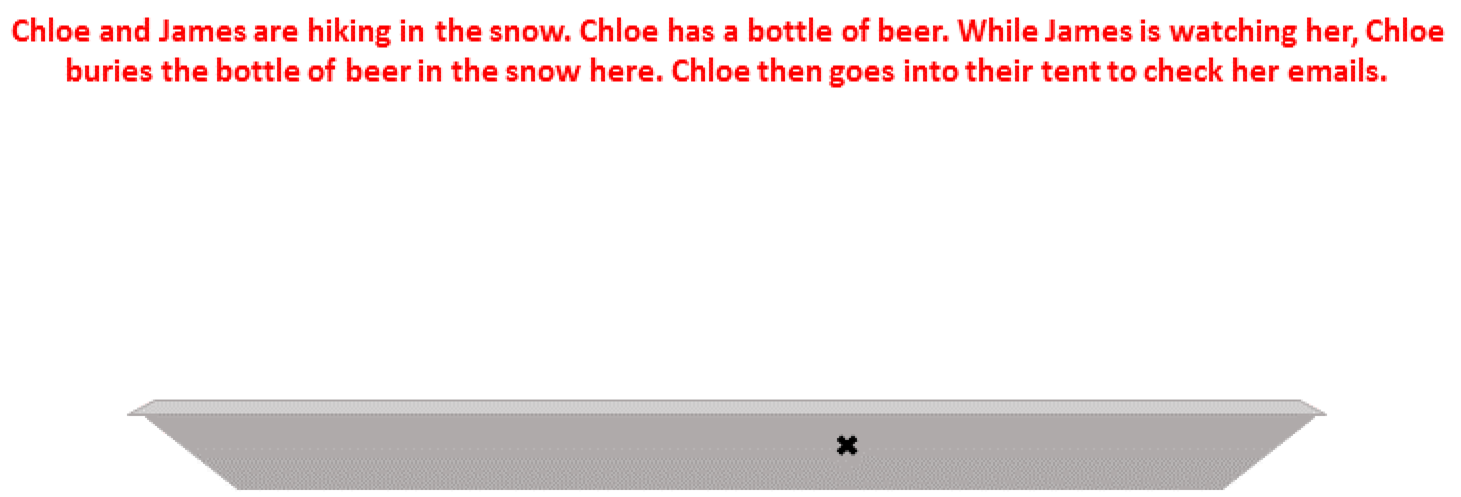

Page Break

Q42

While Chloe is away, James digs the bottle of beer out and hides it here. He smooths over the snow so it looks undisturbed.

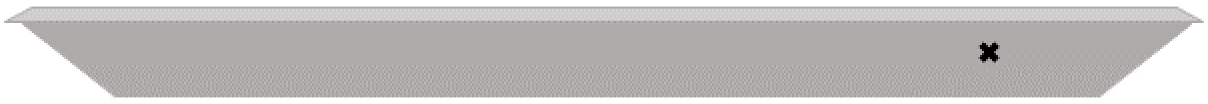

Page Break

Q43
Now try to find as many words as you can in the puzzle below and type them in the field at the bottom of the page. You have 20 seconds!

____

____

____

____

____

Q44 Timing
First Click (1)
Last Click (2)
Page Submit (3)
Click Count (4)

Page Break

Q45
Please click on the image to answer.

After a while, Chloe comes back.

Where do you remember she buried the bottle of beer?

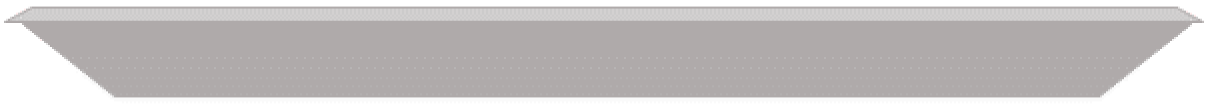

End of Block: Block7

Start of Block: Block8

Q46

Sarah and Alan are on the beach. Sarah has their passports. While Alan is watching her, Sarah buries the passports in the sand here. Sarah then goes to a shop to get some ice cream.

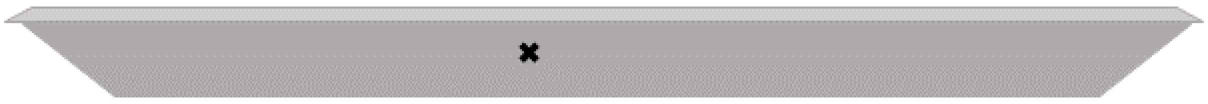

Page Break

Q47

While Sarah is away, Alan digs the passports out and hides them here. He smooths over the sand so it looks undisturbed.

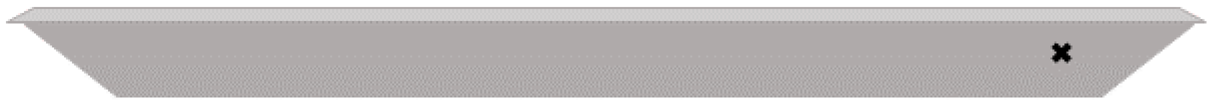

Page Break

Q48
Now try to find as many words as you can in the puzzle below and type them in the field at the bottom of the page. You have 20 seconds!

____

____

____

____

____

Q49 Timing
First Click (1)
Last Click (2)
Page Submit (3)
Click Count (4)

Page Break

Q50
Please click on the image to answer.

After a while, Sarah comes back.

Where do you remember she buried the passports?

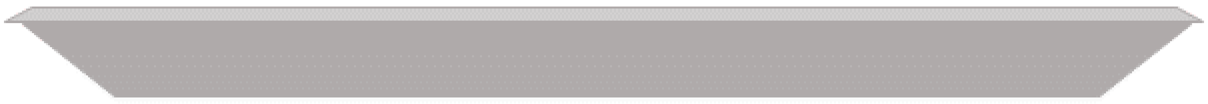

End of Block: Block8

Start of Block: Block9

Q51

John and Alice are in the garden with a planter. John has a flower bulb. While Alice is watching him, John buries the flower bulb in the planter here. John then goes inside to get some water.

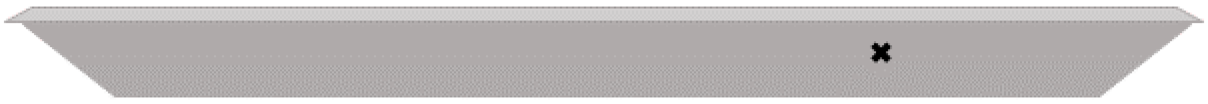

Page Break

Q52

While John is away, Alice digs the flower bulb out and hides it here. She smooths over the soil so it looks undisturbed.

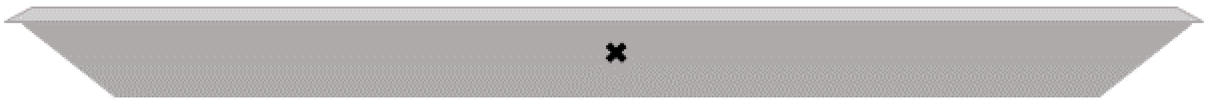

Page Break

Q53
Now try to find as many words as you can in the puzzle below and type them in the field at the bottom of the page. You have 20 seconds!

____

____

____

____

____

Q54 Timing
First Click (1)
Last Click (2)
Page Submit (3)
Click Count (4)

Page Break

Q55
Please click on the image to answer.

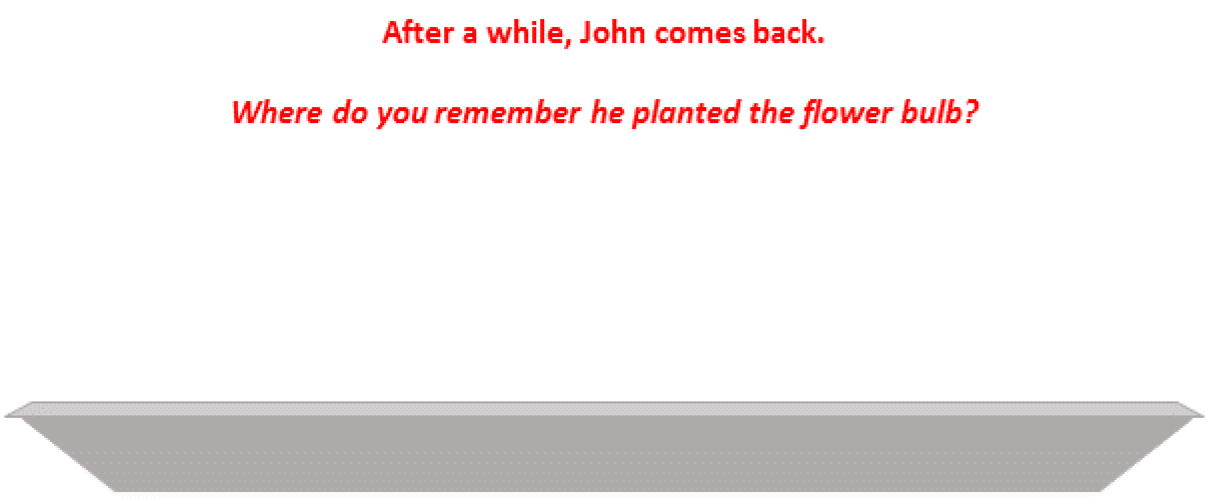

End of Block: Block9

Start of Block: Debrief

Q62 **Participant Debriefing Sheet:**

Thank you for taking part in our study.
What was the study about?
The experiment you took part in is part of a larger study designed to test how people use their own knowledge when having to take into account what others remember or believe. We know that adults tend to expect that other adults know what they themselves know. The critical component of this study was to see whether you were influenced by where you knew an object to be when you had to imagine where someone else remembered or believed it to be.

Page Break

Q62 ***Further Information*** *You are welcome to ask the researchers about the study.*

If you would like to know more about the study you can contact Dr. Steven Samuel (ss2391@cam.ac.uk) for further information.

Page Break

End of Block: Debrief

S	H	U	P	U	F	C
K	U	K	D	R	L	H
K	C	D	D	I	I	A
B	T	O	D	U	R	L
P	S	P	H	E	T	K
T	W	I	N	S	N	I
W	E	F	R	U	C	D

Sally & Jack

D	R	O	L	R	W	L
B	K	E	Y	A	H	R
M	L	A	H	T	F	I
Z	H	G	B	C	E	O
K	K	L	M	E	U	T
K	T	E	G	N	L	N
Y	V	A	E	H	B	S

Tom & Rachel

Y	Z	P	U	B	T	H
R	L	B	U	N	F	I
A	E	L	E	O	X	N
E	C	S	I	Q	S	G
R	B	M	P	H	Z	E
A	Z	O	O	J	C	K
W	H	A	L	E	Y	T

Max & Audrey

C	D	B	I	I	E	S
R	O	Z	S	L	Y	D
O	G	A	K	S	K	R
W	N	C	T	W	E	A
N	U	E	G	J	J	W
S	M	V	Y	I	N	A
S	U	T	O	T	A	L

Rebecca * Steve

Y	C	E	I	Z	V	R
U	L	P	G	E	N	U
I	O	I	U	B	W	K
B	U	N	A	R	I	Y
N	D	K	N	A	G	F
X	D	F	A	P	R	Z
S	D	N	A	H	B	U

Luke & Astrid

F	V	W	Q	Y	B	B
T	E	J	W	C	O	U
T	F	P	T	B	L	R
H	A	I	R	Y	D	N
W	O	R	L	D	L	T
H	C	N	U	P	Y	T
B	L	Y	H	Z	B	B

Peter & Lisa

J	E	E	B	C	A	Z
U	R	N	R	S	S	U
I	T	O	A	B	R	P
C	S	Y	N	T	E	J
E	U	N	D	V	U	K
X	L	A	H	I	V	E
M	O	R	B	I	D	V

Chloe & James

L	A	E	V	J	M	Q
L	E	Z	O	O	I	X
F	F	E	N	N	C	O
M	W	E	Z	O	O	V
G	Y	G	R	A	I	N
D	E	A	T	H	D	D
S	E	I	R	E	S	R

Sarah & Alan

Y	F	H	H	U	H	Y
T	T	R	O	V	X	B
F	O	I	E	O	A	N
W	A	T	V	B	K	H
W	A	R	B	A	G	W
F	Z	L	M	Z	C	P
X	E	L	I	G	H	T

John & Alice
